# Prevalence trends of anemia impairment in adolescents and young adults with HIV/AIDS

**DOI:** 10.1186/s12889-024-18730-4

**Published:** 2024-05-13

**Authors:** Xinqi Li, Nan Zhang, Linlu Ma, Qian Wang, Yuxing Liang, Xiaoyan Liu, Fuling Zhou

**Affiliations:** 1 Department of Hematology, Zhongnan Hospital of Wuhan University, No.169 Donghu Road, Wuhan, 430072 China; 2 School of Nursing, Wuhan University, Wuhan, Hubei China

**Keywords:** Adolescents, Young adults, HIV, Anemia, Prevalence, Global Burden of Diseases

## Abstract

**Background:**

Anemia is a common complication of HIV/AIDS, particularly in adolescents and young adults across various countries and regions. However, little is known about the changing prevalence trends of anemia impairment in this population over time.

**Methods:**

Data on anemia in adolescents and young adults with HIV/AIDS from 1990 to 2019 were collected from the Global Burden of Disease. Prevalence was calculated by gender, region, and country for individuals aged 10–24, and trends were measured using estimating annual percentage changes (EAPC).

**Results:**

Globally, the prevalence of adolescents and young adults with HIV/AIDS increased from 103.95 per 100,000 population in 1990 to 203.78 in 2019. However, anemia impairment has decreased over the past three decades, with a global percentage decreasing from 70.6% in 1990 to 34.7% in 2019, mainly presenting as mild to moderate anemia and significantly higher in females than males. The largest decreases were observed in Central Sub-Saharan Africa, North America, and Eastern Sub-Saharan Africa, with EAPCs of -2.8, -2.34, and -2.17, respectively. Tajikistan (78.76%) and Madagascar (74.65%) had the highest anemia impairment percentage in 2019, while China (16.61%) and Iceland (13.73%) had the lowest. Anemia impairment was closely related to sociodemographic index (SDI) levels, with a high proportion of impairment in low SDI regions but a stable decreasing trend (EAPC = -0.37).

**Conclusion:**

Continued anemia monitoring and management are crucial for patients with HIV, especially in high-prevalence regions and among females. Public health policies and interventions can improve the quality of life and reduce morbidity and mortality.

**Supplementary Information:**

The online version contains supplementary material available at 10.1186/s12889-024-18730-4.

## Background

Acquired immune deficiency syndrome (AIDS) caused by the human immunodeficiency virus (HIV) has been a major public health issue, affecting millions of people worldwide, with a growing proportion of cases in adolescents and young adults [1]. Despite the widespread availability of antiretroviral therapy (ART) over the past few decades, HIV-infected individuals continue to face numerous challenges, including changes in hematological parameters that may impact their health and survival [2, 3]. Anemia has been identified as a prognostic marker of disease progression in HIV infection and is associated with reduced survival rates [4, 5].

Anemia in patients with HIV involves multiple mechanisms, including the direct impact of the virus on bone marrow, where it infects and kills red blood cell-producing cells. The virus also stimulates cytokine production that inhibits erythropoiesis [6]. Chronic inflammation, commonly seen in patients with HIV, can increase hepcidin, which reduces iron availability crucial for red blood cell production [7, 8]. ART can also cause anemia, with drugs like zidovudine damaging bone marrow and inhibiting red blood cell production [9, 10]. Furthermore, some patients may experience drug-induced hemolysis, leading to faster destruction of red blood cells [11]. The risk of anemia increases as the lifespan of patients with HIV extends due to improved treatment and control [12]. Adolescents and young adults with HIV have a higher proportion of anemia, although this can vary across regions and gender [13–15].

Adolescence is a transitional phase of life that encompasses biological growth, as well as social and behavioral changes. The WHO defines adolescence as ranging from 10–19 years of age [16]. For this study, adolescents were defined as individuals between the ages of 10–19 years and young adults as individuals between the ages of 20–24 years. Age subgroups were utilized to further elucidate the nuances of adolescent growth, distinguishing between younger adolescents (10–14 years), older adolescents (15–19 years), and young adults (20–24 years) to provide a more detailed description of this life phase as well as its popular understanding [17].

The Global Burden of Disease (GBD) project has significantly transformed our understanding of global health by providing comprehensive and standardized estimates of diseases, injuries, and risk factors, thanks to outstanding cooperation among governments worldwide [18]. One key benefit of this resource is that it offers insights into the epidemiological trends of HIV/AIDS and the anemia damage caused by the disease. We evaluated the anemia burden among adolescents and young adults with HIV/AIDS by analyzing global, regional, and national trends from 1990 to 2019. Our goal was to understand the changing prevalence and severity of anemia in this population over the past three decades.

## Methods

### Data sources and data collection

For this study, we analyzed the Global Health Data Exchange (GHDx), which provided cross-sectional data on the GBD 2019 of 369 diseases and injuries in 204 countries and territories from 1990 to 2019, including HIV/AIDS [18]. The primary diagnosis of HIV/AIDS was determined using the primary procedures and diagnostic codes developed by the World Health Organization (WHO) [18]. Anemia was defined as a decrease in hemoglobin (Hb) concentration in the blood, regardless of the underlying cause, red blood cell morphology, or red blood cell function [19]. The severity of anemia was classified as mild, moderate, or severe based on the thresholds for hemoglobin in g/L as defined by the WHO (https://www.who.int/news-room/fact-sheets/detail/anaemia) [20]. Further details on the definitions of anemia severity in this study can be found in Table 1.

**Table 1:** Definitions of mild, moderate, and severe anaemia, based on blood haemoglobin concentration

Characteristics	Age group			
	**5 to 14 years**	**15 to 24 years**		
**Sex**	Both	Male	Female, non-pregnant	Female, pregnant
**Mild (g/L)**	110 to 114	110 to 129	110 to 119	100 to 109
**Moderate (g/L)**	80 to 109	80 to 109	80 to 109	70 to 99
**Severe (g/L)**	< 80	< 80	< 80	< 70

Data were collected on HIV/AIDS and anemia severity levels from individuals of both sexes, categorized by three distinct age groups (10–14 years, 15–19 years, and 20–24 years), and across 21 regional groupings of countries that are geographically close and epidemiologically similar, as defined by the GBD project [18]. The SDI is a measure of countries’ overall development, determined by factors such as educational attainment, average income per person, and total fertility rate. It ranges from 0 to 1, with lower values indicating lower educational attainment, lower income per capita, and higher fertility rates across all GBD regions. Additionally, the SDI is categorized into low, low-middle, middle, high-middle, and high quintiles [21, 22].

### Statistical analysis

To measure the prevalence trends of HIV/AIDS and anemia in different regions, we utilized prevalence rates, percentages, and estimated annual percentage changes (EAPC) [23–25]. The EAPCs, along with their corresponding 95% confidence intervals (CIs), provide a comprehensive assessment of the trends in prevalence over a specified period, where a lower bound above 0 indicates an upward trend, while an upper bound below 0 indicates a downward trend. We use a log-linear regression to calculate (where y = ln(rates), and x = calendar year), as follows:

$$y=\alpha+\beta x+\varepsilon$$

$$EAPC=100\%\times\left(e^\beta-1\right)$$

Additionally, we conducted Pearson correlation analyses to explore the relationships between the EAPC and the percentage of anemia in 1990, the EAPC and the sociodemographic index (SDI) in 2019, as well as the percentage of anemia and SDI for all HIV/AIDS cases. The resulting ρ indices and *p*-values were used to evaluate the strength and significance of the correlations. We also created global maps of HIV/AIDS prevalence and anemia percentage by country, depicting prevalence rates in 2019, the percentage change in cases, and EAPC trends from 1990 to 2019. All statistical analyses and data visualizations were performed using R software (version 3.5.2) and GraphPad Prism (version 8.02).

## Results

### Global prevalence estimates of HIV/AIDS

**Fig. 1:**
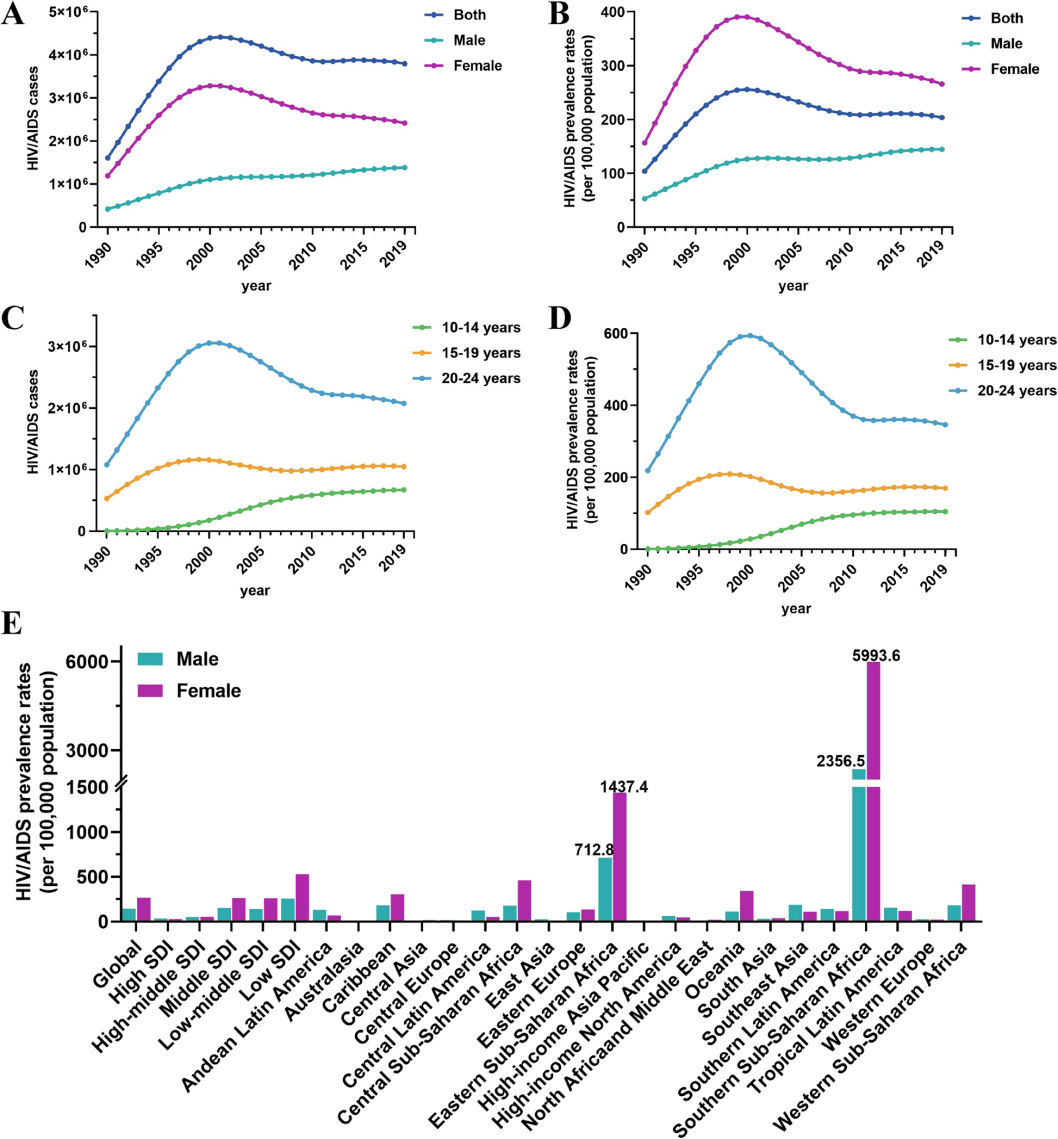
Prevalence trends of HIV/AIDS in adolescents and young adults from 1990 to 2019. **A** Number of cases of HIV/AIDS by sex. **B** Prevalence rates of HIV/AIDS by sex. **C** Number of cases of HIV/AIDS by age group. **D** Prevalence rates of HIV/AIDS by age group. **E** The prevalence rates of HIV/AIDS in males and females globally, in territories with low to high SDIs and in 21 GBD regions in 2019

Globally, the prevalence and change trends with HIV/AIDS in adolescents and young adults from 1990 and 2019 are presented in Fig. 1. The number of cases increased from 1,610,147 in 1990 to 3,794,156 in 2019, as shown in Fig. 1A. Although the prevalence was significantly higher in women than in men, the upward trend was more pronounced in men, with an EAPC of 2.45 in men and 0.17 in women. In Fig. 1B, it can be observed that the global prevalence of HIV/AIDS has shown a downward trend in recent years, reaching 203.78 per 100,000 in 2019. Since then, the prevalence has been on a declining trend, reflecting the WHO’s goal and determination to eliminate HIV/AIDS as a major global health challenge by 2030 [26]. For different age groups, although the prevalence of HIV/AIDS is higher among slightly older individuals, the 15–19 age group (EAPC = 0.18) and 20–24 age group (EAPC = -0.34) have remained relatively stable over the past 30 years (Fig. 1C-D). The prevalence among the 10–14 age group has sharply increased (EAPC = 17.27), indicating a trend of HIV infections becoming more prevalent among younger individuals. Especially, the prevalence rates varied greatly among different SDI levels, ranging from 30–400 per 100,000 in 2019 (Supplementary Fig. S1A). The highest prevalence rates were observed in Southern Sub-Saharan Africa and Eastern Sub-Saharan Africa, with rates reaching 2356.5 and 712.8 per 100,000 for males and 5993.6 and 1437.4 per 100,000 for females, respectively (Fig. 1E). Over the past 30 years, the number of cases has increased in almost all countries, some African countries have high prevalence rates, such as Eswatini, Lesotho, and Mozambique with rates reaching 6413, 5640.74, and 4334.39 per 100,000 in 2019, respectively. Additional detailed visual data for each country can be found in the Supplementary Fig. S1B-D.

### Global proportion of anemia impairment in HIV/AIDS

**Fig. 2:**
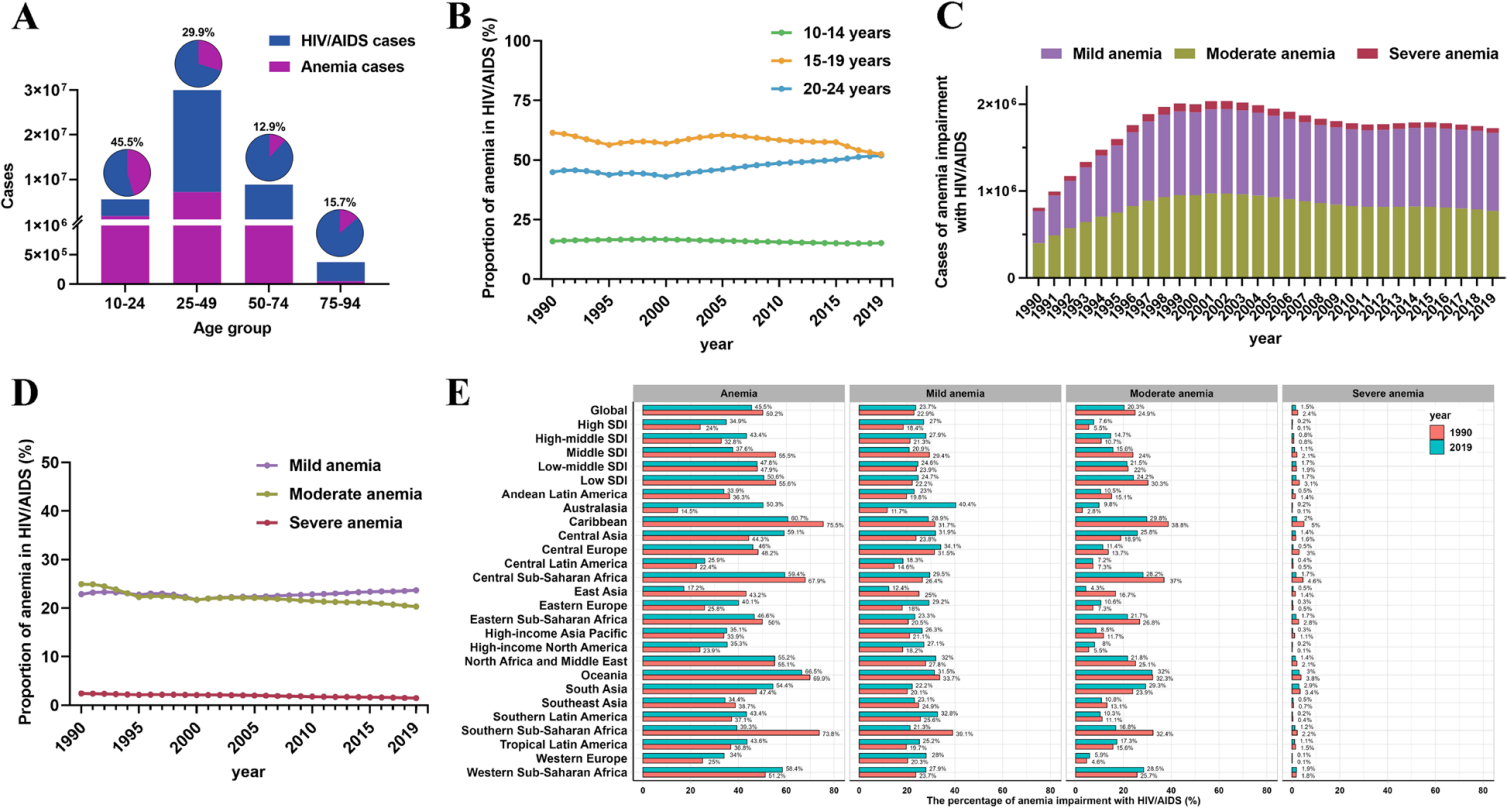
The proportion of anemia impairment with HIV/AIDS. **A** Number of cases of HIV/AIDS and anemia cases in different age groups. **B** The percentage of anemia impairment in adolescents and young adults with HIV/AIDS by age group from 1990–2019. **C** Number of cases of anemia impairment in adolescents and young adults with HIV/AIDS by severity from 1990–2019. **D** The percentage of anemia impairment in adolescents and young adults with HIV/AIDS by severity from 1990–2019. **E** The percentage of anemia impairment in adolescents and young adults with HIV/AIDS by severity between 1990 and 2019, in territories with low to high SDIs and in 21 GBD regions

Anemia caused by hematological abnormalities is the most common complication of HIV/AIDS, leading to changes in the overall disease burden [5]. Therefore, we further analyzed the percentage of anemia in HIV/AIDS (Table 2). We divided global HIV/AIDS cases into four age groups, and although the largest number of cases were in Young and middle-aged (25–49 years), adolescents and young adults (10–24 years) had the highest percentage of anemia, which was 2–3 times higher than other age groups, reaching 45.5% in 2019 (Fig. 2A). From 1990 to 2019, the percentage of anemia among different age groups varied. The lowest percentage was found in the 10–14 age group, which remained stable at around 15% (EAPC = -0.36), while the highest percentage was in the 15–19 age group, which showed a declining trend until 2019 when it became similar to the 20–24 age group, at around 52% (Fig. 2B). When categorized by the severity of anemia, the majority of cases with anemia belonged to the mild or moderate categories, and the variation trend of different severity levels of anemia was consistent with the total number of cases (Fig. 2C). Fortunately, the prevalence of severe anemia was the lowest, accounting for less than 3%, and the trend was still declining (EAPC = -1.58). The hotspot of moderate anemia has gradually shifted to mild anemia (Fig. 2D). For the 21 geographical regions, most regions have shown improvement in the percentage of anemia, such as Central Sub-Saharan Africa (EAPC = -2.87), High income North America (EAPC = -2.34), and Eastern Sub-Saharan Africa (EAPC = -2.17). However, as of 2019, the percentage of anemia in regions Australasia, the Caribbean, Central Asia, Central Sub-Saharan Africa, North Africa and Middle East, Oceania, South Asia, and Western Sub-Saharan Africa still exceeded 50% (Fig. 2E), showing different percentage levels of anemia in different regions.

**Table 2:** The global prevalence percentage and their change trends of anemia impairment in adolescents and young adults with HIV/AIDS from 1990 to 2019

Characteristics	1990		2019		1990–2019
	**Cases, n**	**Percent, %**	**Cases, n**	**Percent, %**	**EAPC**
**Global**	807944 (708833 to 912542)	50.22 (46.53 to 53.73)	1725043 (1597619 to 1856783)	45.47 (42.63 to 48.21)	-0.28 (-0.35 to -0.2)
**Sex**					
Male	46898 (39505 to 55342)	11.26 (9.77 to 12.78)	175316 (161983 to 191225)	12.72 (11.65 to 13.73)	0.23 (0.11 to 0.35)
Female	761,046 (662760 to 863906)	63.91 (58.48 to 68.73)	1549727 (1429052 to 1671728)	64.21 (59.89 to 68.58)	0.21 (0.07 to 0.35)
**Age group**					
10–14 years	399 (174 to 887)	15.84 (13.6 to 18.43)	101142 (91245 to 111500)	15.09 (14.2 to 16.07)	-0.36 (-0.44 to -0.28)
15–19 years	324812 (276934 to 375759)	61.4 (55.78 to 66.39)	549200 (494571 to 607921)	52.38 (48.16 to 56.25)	-0.24 (-0.36 to -0.11)
20–24 years	482733 (420956 to 547341)	44.82 (41.23 to 48.9)	1074701 (982299 to 1172799)	51.82 (47.61 to 56.06)	0.57 (0.46 to 0.68)
**Severity**					
Mild anemia	368219 (318040 to 419853)	22.88 (21.39 to 24.4)	898128 (826978 to 971968)	23.67 (22.19 to 25.11)	0.08 (-0.01 to 0.17)
Moderate anemia	400906 (351110 to 452748)	24.93 (22.62 to 27.16)	771151 (703270 to 840303)	20.33 (18.83 to 21.94)	-0.53 (-0.62 to -0.44)
Severe anemia	38819 (32018 to 46550)	2.42 (2.02 to 2.86)	55763 (48609 to 64448)	1.47 (1.28 to 1.69)	-1.58 (-1.69 to -1.48)
**SDI**					
High SDI	27477 (14279 to 46136)	24.01 (20.25 to 26.98)	19547 (12475 to 29152)	34.9 (27.38 to 40.41)	0.55 (0.25 to 0.85)
High-middle SDI	26018 (19929 to 33141)	32.83 (29.91 to 35.97)	57670 (45061 to 72654)	43.36 (38.1 to 47.39)	0.4 (0.13 to 0.68)
Middle SDI	68540 (58108 to 80464)	55.49 (50.18 to 60.85)	424992 (357027 to 504765)	37.58 (32.37 to 43.22)	-1.3 (-1.47 to -1.14)
Low-middle SDI	195,592 (149039 to 241514)	47.86 (39.91 to 53.32)	484,317 (440579 to 533921)	47.81 (44.85 to 50.65)	0.58 (0.35 to 0.81)
Low SDI	489386 (412114 to 570665)	55.62 (51.17 to 60.41)	736963 (656951 to 819564)	50.63 (46.6 to 54.19)	-0.37 (-0.47 to -0.27)
**Region**					
Andean Latin America	1624 (1062 to 2804)	36.27 (32.34 to 41.7)	5761 (3340 to 9438)	33.95 (28.69 to 41.27)	-0.16 (-0.22 to -0.09)
Australasia	133 (73 to 219)	14.49 (10.52 to 20.31)	303 (179 to 485)	50.32 (43.08 to 57.22)	3.79 (3.18 to 4.4)
Caribbean	17231 (10065 to 26391)	75.47 (66.39 to 82.73)	16911 (12788 to 22378)	60.65 (56.22 to 65.97)	-1 (-1.11 to -0.9)
Central Asia	374 (215 to 600)	44.26 (39.96 to 48.47)	1882 (1095 to 2727)	59.13 (56.65 to 61.32)	1.21 (1.11 to 1.32)
Central Europe	354 (205 to 565)	48.17 (43.84 to 51.44)	1308 (892 to 1787)	46.01 (37.7 to 50.72)	-0.22 (-0.34 to -0.1)
Central Latin America	2881 (1842 to 4259)	22.39 (20.99 to 23.85)	15071 (8595 to 22722)	25.88 (22.74 to 28.45)	0.5 (0.14 to 0.87)
Central Sub-Saharan Africa	67960 (48895 to 92539)	67.91 (60.29 to 76.37)	81595 (62251 to 105908)	59.35 (51.39 to 66.49)	-0.49 (-0.65 to -0.32)
East Asia	4845 (3051 to 6634)	43.21 (38.12 to 46.48)	7468 (4004 to 12,944)	17.18 (13.73 to 20.79)	-3.19 (-3.38 to -3)
Eastern Europe	1690 (987 to 2809)	25.83 (18.79 to 32.37)	15454 (8378 to 23968)	40.13 (24.95 to 49.87)	1.73 (1.58 to 1.87)
Eastern Sub-Saharan Africa	445570 (384733 to 506383)	50.03 (45.82 to 54.2)	700443 (623612 to 776355)	46.64 (42.8 to 50.16)	-0.09 (-0.2 to 0.02)
High-income Asia Pacific	311 (113 to 544)	33.89 (31.41 to 36.56)	1035 (695 to 1508)	35.1 (32.16 to 38.51)	-0.03 (-0.14 to 0.08)
High-income North America	22609 (11122 to 39260)	23.85 (19.57 to 27.26)	13949 (7891 to 22073)	35.34 (24.98 to 42.87)	0.46 (0.12 to 0.8)
North Africa and Middle East	1699 (760 to 4121)	55.09 (44.37 to 69.75)	14503 (5692 to 34336)	55.24 (43.14 to 69.23)	-0.13 (-0.22 to -0.04)
Oceania	63 (28 to 187)	69.85 (56.58 to 80.07)	5992 (294 to 19071)	66.49 (52.71 to 76.81)	-0.51 (-0.83 to -0.2)
South Asia	4968 (2494 to 10,427)	47.41 (43.22 to 51.18)	98546 (75133 to 141082)	54.44 (48.95 to 58.61)	0.35 (0.21 to 0.5)
Southeast Asia	10750 (7302 to 15168)	38.71 (35.01 to 42.53)	87125 (64190 to 117942)	34.38 (30.37 to 38.98)	-0.78 (-1.09 to -0.48)
Southern Latin America	4349 (2485 to 6663)	37.08 (31.98 to 42.35)	8488 (4477 to 14035)	43.41 (37.23 to 49.61)	-0.02 (-0.25 to 0.21)
Southern Sub-Saharan Africa	104363 (64522 to 146855)	73.76 (46.48 to 84.88)	348570 (287713 to 423632)	39.28 (32.6 to 46.13)	-1.37 (-1.84 to -0.89)
Tropical Latin America	16659 (10356 to 24856)	36.78 (32.93 to 41.11)	31039 (19877 to 42729)	43.62 (41 to 46.74)	0.07 (-0.23 to 0.38)
Western Europe	10497 (7318 to 14107)	25.02 (21.19 to 28.96)	5753 (4311 to 7823)	34 (28.81 to 38.71)	0.98 (0.79 to 1.19)
Western Sub-Saharan Africa	89016 (61045 to 124935)	51.2 (44.97 to 57.09)	263,848 (231581 to 298680)	58.35 (54.52 to 61.72)	0.55 (0.47 to 0.64)

Data in parentheses are 95% uncertainty intervals for cases and prevalence, and 95% confidence intervals for EAPC

*Abbreviations: EAPC* Estimated annual percentage change, *SDI* Social development index

### Regional differences of anemia impairment in HIV/AIDS

**Fig. 3:**
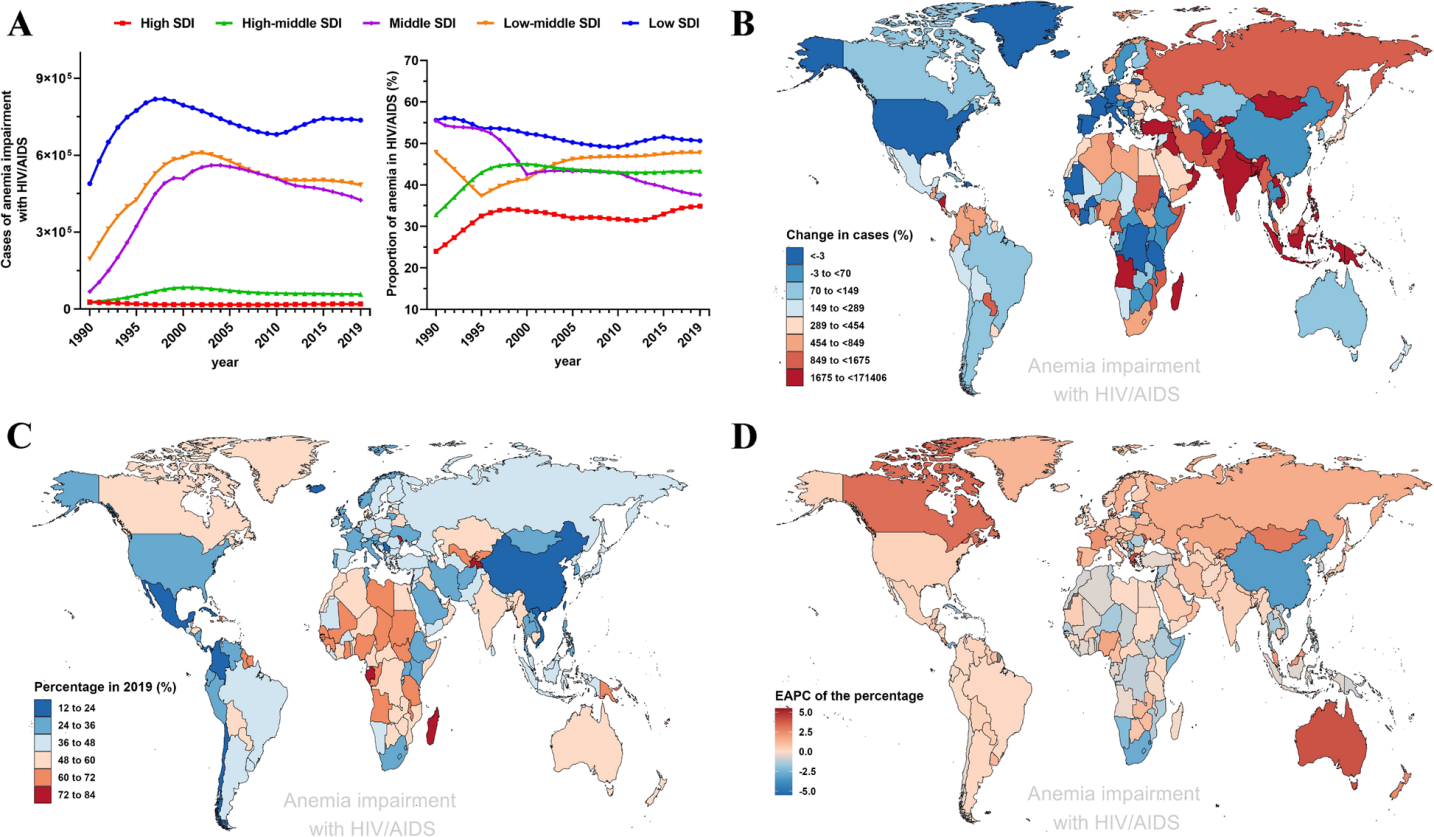
Prevalence of anemia impairment in adolescents and young adults with HIV/AIDS by countries and territories combined for both sexes. **A** Number of cases and prevalence by SDI regions. **B** The percentage change in cases between 1990 and 2019. **C** Proportion in 2019. **D** The EAPC of the percentage from 1990 to 2019. EAPC, estimated annual percentage change

On a global scale, the proportion and absolute number of cases in each group vary greatly due to the level of socioeconomic development and geographical location [4]. In general, regions with high SDI have low numbers of cases and anemia percentages, while regions with low SDI have high numbers of cases and anemia percentages. However, there is a slight upward trend in percentage of anemia in the high SDI (EAPC = 0.55) and middle-high SDI (EAPC = 0.4) regions (Fig. 3A). For each country, almost all countries have experienced an increase in percentage of anemia compared to 1990, which is related to changes in the world’s population density (Fig. 3B and Supplementary Table S1). Furthermore, Tajikistan and Madagascar have high anemia percentages among HIV/AIDS-infected individuals, at 78.76% and 74.65%, respectively, while China and Iceland have low anemia percentages at 16.61% and 13.73%, respectively (Fig. 3C and Supplementary Table S1). Regarding the trend of anemia percentage over the past 30 years, Cyprus, Greece, and Australia had the greatest increase in the trend, from 16.35%, 17.25%, and 14% in 1990 to 44.72%, 43.6%, and 50.82% in 2019, respectively (Fig. 3D and Supplementary Table S1). In contrast, Qatar, Eswatini, and Lithuania have the most significant downward trend, decreasing from 46.3%, 84.04%, and 59.69% in 1990 to 18.18%, 26.55%, and 28.97% in 2019, respectively (Fig. 3D and Supplementary Table S1).

In 2019, the countries with the highest proportion of mild anemia were Denmark (52.11%), the Republic of Moldova (46.56%), and Barbados (46.42%). The countries with the highest proportion of moderate anemia were Gambia (39.27%), Gabon (37.86%), and Mali (36.29%). The countries with the highest proportion of severe anemia were Tajikistan (3.62%), Mali (3.33%), and Papua New Guinea (3.13%) (Supplementary Table S1). Moreover, the countries with the most significant increase in the proportion of mild anemia in the last 30 years were Cyprus (EAPC = 5.26), Australia (EAPC = 3.99), and Greece (EAPC = 3.93), while the countries with the most significant decrease were Lithuania (EAPC = -3.32), Qatar (EAPC = -3.26), and Eswatini (EAPC = -3.09). The countries with the most significant increase in the proportion of moderate anemia were Cyprus (EAPC = 5.91), Greece (EAPC = 4.99), and Australia (EAPC = 4.12), while the countries with the most significant decrease were Eswatini (EAPC = -5.26), Qatar (EAPC = -5.23), and Lebanon (EAPC = -5.09). The countries with the most significant increase in the proportion of severe anemia were Australia (EAPC = 4.96), Czechia (EAPC = 4.56), and New Zealand (EAPC = 4.27), while the countries with the most significant decrease were Equatorial Guinea (EAPC = -7.92), Serbia (EAPC = -7.57), and Vietnam (EAPC = -7.45) (Supplementary Table S1). These findings indicate that there is a large variation in anemia prevalence combined with HIV/AIDS across countries and different time trends have been observed over the past 30 years.

### Gender differences of anemia impairment in HIV/AIDS

**Fig. 4:**
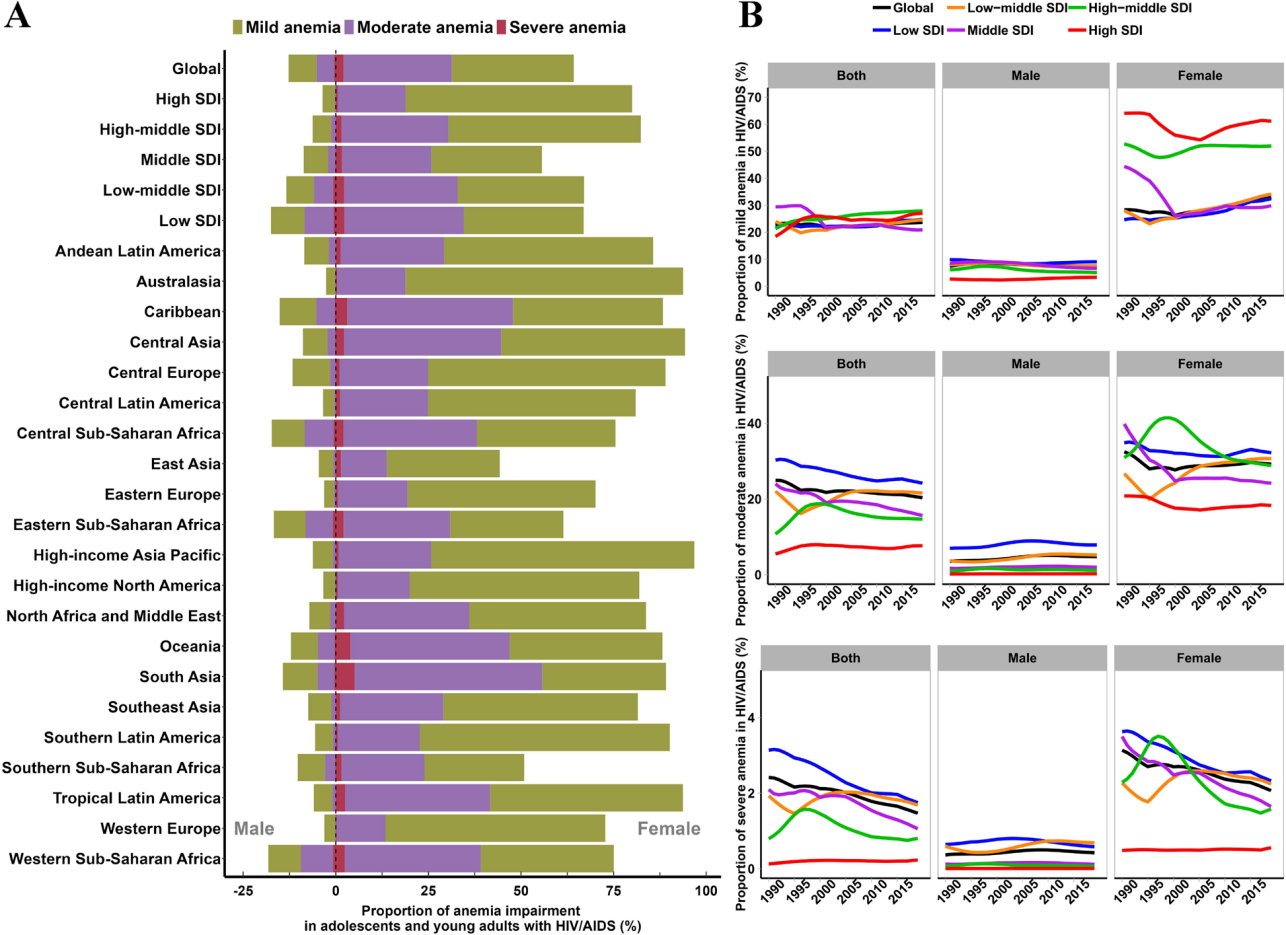
The global burden of anemia impairment in adolescents and young adults with HIV/AIDS by sex. **A** The percentage of anemia impairment in HIV/AIDS by severity between 1990 and 2019, in territories with low to high SDIs and in 21 GBD regions. **B** Proportion of anemia impairment in HIV/AIDS by sex and severity from 1990 to 2019

Although the severity of anemia in different genders has strict criteria for assessment, there is still a significant gender difference in the percentage of anemia damage caused by HIV/AIDS among adolescents and young adults, with females consistently bearing a greater burden of anemia, which is related to female hormones, menstruation, genetics, and other factors [27, 28]. In 2019, the global proportions of mild, moderate, and severe anemia in males were 7.5%, 4.8%, and 0.4%, respectively, while in females, the proportions were 32.9%, 29.2%, and 2.1%, respectively (Fig. 4A). The proportion of anemia among males was closely associated with the SDI regions, with a higher proportion of anemia in regions with lower SDI. However, there was no significant correlation between the proportion of anemia among females and the SDI regions, with higher SDI regions having a higher proportion of anemia. For example, the proportion of anemia impairment in high SDI regions is 79.9%, in middle-high SDI regions it is 82.3%, and in middle SDI regions it is the lowest, at 55.6% (Fig. 4A). In the high SDI regions, males consistently exhibited the lowest prevalence of anemia impairment from 1990 to 2019, across mild, moderate, and severe categories. Conversely, while females in high SDI regions also had low prevalence of moderate and severe anemia impairment, their prevalence of mild anemia impairment was higher in high SDI regions (Fig. 4B). There are also significant gender differences in different regions, with the highest proportion of male impairment occurring in Western Sub-Saharan Africa, Central Sub-Saharan Africa, and Eastern Sub-Saharan Africa, and the highest proportion of female impairment occurring in Asia Pacific, Central Asia, and Australasia (Fig. 4A).

### Correlations of EAPC, prevalence, and SDI

After analyzing the prevalence trends of anemia impairment among adolescents and young adults with HIV/AIDS, we further investigated related factors such as EAPC values and SDI levels. EAPC showed a strong negative correlation with the initial anemia percentage (*ρ* = -0.604, *p* < 0.001), and a weak correlation with the latest SDI (*ρ* = 0.32, *p* < 0.001), indicating that some countries with initially high anemia burden have shown improvement, which is related to their level of economic development (Supplementary Fig. S2A-B). As for SDI levels, we noticed that the anemia percentage in lower SDI regions is still on the rise, but overall, SDI levels are significantly negatively correlated with anemia percentage (*ρ* = -0.524, *p* < 0.001) (Supplementary Fig. S2C). When aggregated at the national level, the fitted curve shows that the negative correlation between SDI levels and anemia percentage still exists (*ρ* = -0.373, *p* < 0.001) (Supplementary Fig. S2D), indicating that the anemia burden is still heavy in countries with low SDI levels.

## Discussion

This study provides new insights into the changing prevalence trends of anemia impairment in this population [29]. The results show that the prevalence of anemia impairment has been decreasing, suggesting that the current screening and management strategies are effective. However, further research is needed to identify the specific factors contributing to this decline and to develop more targeted interventions to address anemia in this vulnerable population [30]. These findings emphasize the importance of continuous monitoring and management of anemia impairment in adolescents and young adults with HIV/AIDS to improve their quality of life and reduce morbidity and mortality.

Previous research has reported on the trends in HIV and other sexually transmitted infection (STI) burden among 10–24-year-old adolescents and young adults globally, regionally, and nationally from 1990 to 2019 [16]. The research indicates that HIV incidence among adolescents and young adults has decreased from 34.5 per 100,000 in 1990 to 22.7 per 100,000 in 2019, which is consistent with the implementation of ART and pre-exposure prophylaxis. On the other hand, the incidence of other STIs has increased from 6986.3 per 100,000 in 1990 to 7088.7 per 100,000 in 2019. The global decline rate of other STIs incidence from 2009 to 2019 was approximately one-fifth of the decline rate of global HIV incidence during the same period [16]. In our study, we also investigated the burden of HIV/AIDS, but our focus was on estimating the global prevalence of HIV/AIDS and the proportion of anemia impairment in HIV/AIDS globally. Our findings show that the global HIV/AIDS prevalence decreased from 255.75 per 100,000 in 2000 to 203.78 per 100,000 in 2019, and the proportion of anemia impairment in HIV/AIDS decreased from 50.22% in 1990 to 45.47% in 2019. These results are consistent with previous research and indicate a decline in HIV/AIDS burden among adolescents and young adults globally [4].

In our study, the region with the highest burden of HIV/AIDS and anemia damage in 2019 was Oceania, reaching 66.5%. While the proportion of anemia damage in sub-Saharan Africa was initially high at 73.8%, through persistent control efforts, it decreased to only 39.3% in 2019 with an EAPC of -1.37. The distribution of anemia damage of different degrees varies among regions. Oceania had a higher proportion of moderate anemia impairment, while Australasia had a higher proportion of mild anemia impairment. These variations are related to the global distribution of anemia prevalence [31]. Additionally, low SDI areas have a higher incidence of anemia damage, while high SDI areas have a higher absolute number of anemia damage cases. These findings suggest that measures and strategies tailored to local conditions may be needed to address anemia [32].

Gender has a significant effect on the prevalence of anemia, with women being more likely to suffer from anemia than men due to a variety of factors such as menstrual blood loss, pregnancy, lactation, and nutritional deficiencies [28]. Women may also face limited access to healthcare services, including routine check-ups, and may be more likely to have poor diets due to lower socioeconomic status [33]. All of these factors can contribute to a higher prevalence of anemia among women. The effect of gender on the impairment of HIV/AIDS anemia is also worth noting [34]. In particular, the prevalence of HIV/AIDS is higher in women, and HIV/AIDS infection can exacerbate the development and progression of anemia. HIV/AIDS can directly cause anemia through the destruction of red blood cells or by interfering with the production of new red blood cells [3, 6]. Additionally, opportunistic infections and medications used to treat HIV/AIDS can also contribute to anemia in people living with HIV/AIDS [10]. Overall, understanding the gender differences in the prevalence and impairment of anemia in the context of HIV/AIDS is crucial for developing effective interventions and treatment strategies that address the unique needs of women and men living with HIV/AIDS. In addition, we also analyzed the correlation between EAPC, prevalence, and SDI. Our study found a negative correlation coefficient, indicating a decrease in the prevalence of anemia impairment in HIV/AIDS over time, which may be due to the success of current screening and management strategies. In low SDI regions, the heavy burden of HIV-associated anemia persists, exacerbated by limited healthcare resources, poor nutrition, stigma, economic constraints, and concurrent health challenges such as malaria [35]. However, the need for strengthening anemia management in low SDI areas persists, highlighting the ongoing disparities in healthcare access and resources.

Improving the quality of life for individuals with HIV requires a comprehensive approach. Accessible healthcare services, including testing and treatment, are crucial, as are psychological support and stigma reduction efforts. Promoting sexual education and awareness is vital for prevention. By integrating these measures, we can enhance the well-being of those living with HIV and reduce the burden of the disease on society. A study in Vietnam recommends screening for depression and intervening in the lives of individuals who start antiretroviral therapy late. In addition, Kirabo is a tool focused on reducing HIV infection risks among Ugandan youth [36]. It provides local HIV clinic and PrEP information, conducts behavioral health screenings, and facilitates necessary referrals. By increasing youth access to prevention strategies and reducing susceptibility factors, Kirabo contributes to global efforts to mitigate HIV prevalence [37].

## Limitations

While providing a comprehensive overview of changing prevalence trends of anemia impairment in adolescents and young adults with HIV/AIDS, the current study has some limitations. Similar to issues with many diseases from the GBD study, the accuracy of prevalence trends depends on the quality and quantity of input data, which have been detailed in previously published GBD studies [16, 31]. Data collected from different regions and countries may vary considerably in terms of quality, comparability, accuracy, and degree of missing data, leading to some deviation in estimates, even if data is adjusted using multiple statistical methods. Moreover, underreporting and failure to diagnose in less developed countries with limited clinical can be sources of bias in the registration of HIV/AIDS and anemia severity, leading to underestimation of certain disease data. Despite these limitations, the study provides valuable insights into the prevalence trends of anemia impairment in adolescents and young adults with HIV/AIDS and can help guide future research and interventions in this area.

## Conclusions

In conclusion, our study emphasizes the importance of ongoing monitoring and management of anemia impairment in adolescents and young adults with HIV/AIDS, particularly in regions with higher anemia impairment rates, lower levels of socioeconomic development, and among female populations. In contrast, males in high SDI regions exhibit a significantly lower prevalence of anemia impairment. These findings can inform public health policies and interventions aimed at improving the quality of life and reducing morbidity and mortality in this vulnerable population. Overall, our study highlights the critical need for increased awareness, resources, and efforts to address anemia and its underlying causes in the context of HIV/AIDS.

### Supplementary Information

Supplementary Material 1.

## Data Availability

The data are available from the Global Burden of Disease Results Tool of the Global Health Data Exchange (http://ghdx.healthdata.org/). All results generated in this study can be found in the supplementary material.

## Abbreviations

ART: Antiretroviral Therapy
AIDS: Acquired Immune Deficiency Syndrome
CIs: Confidence Intervals
EAPC: Estimated Annual Percentage Changes
GBD: Global Burden of Disease
GHDx: Global Health Data Exchange
HIV: Human Immunodeficiency Virus
SDI: Socio-Demographic Index
STI: Sexually Transmitted Infection
WHO: World Health Organization
