# Supplementary material for: Prevalence trends of anemia impairment in adolescents and young adults with HIV/AIDS
Source: BMC Public Health. 2024 May 13;24:1301. doi: 10.1186/s12889-024-18730-4 (PMC11092044; doi:10.1186/s12889-024-18730-4)
Supplement: Supplementary file 1 — Supplementary Material 1. [file 12889_2024_18730_MOESM1_ESM.pdf]

Supplementary material

Prevalence Trends of Anemia Impairment in Adolescents and Young Adults with HIV/AIDS

Supplementary material 1

Supplementary Figure S1. Prevalence of HIV/AIDS in adolescents and young adults by countries and territories combined for both sexes. 2

Supplementary Figure S2. Correlation of EAPC, prevalence, and SDI of anemia impairment in adolescents and young adults with HIV/AIDS. 3

Supplementary Table S1. The prevalence percentage and their change trends of anemia impairment in adolescents and young adults with HIV/AIDS for all genders at national level from 1990 to 2019. 4

Supplementary Figure S1. Prevalence of HIV/AIDS in adolescents and young adults by countries and territories combined for both sexes.

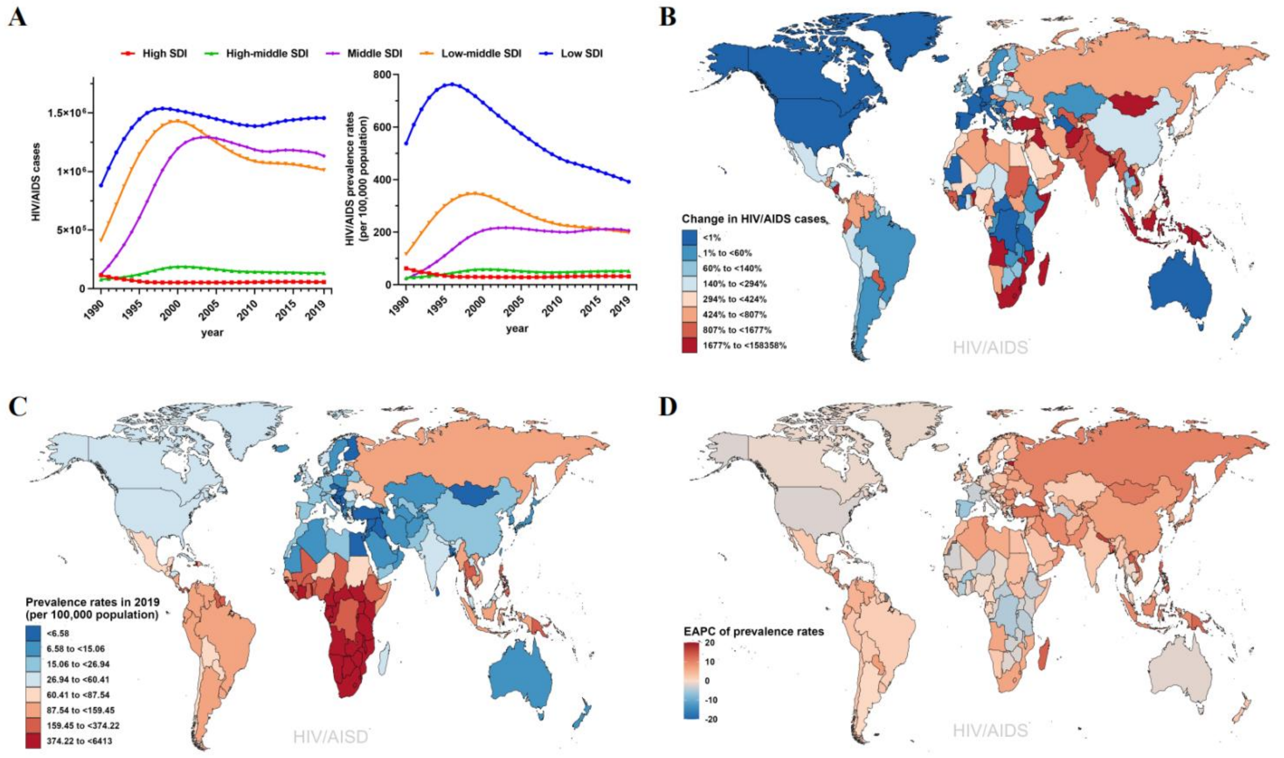

(A) Number of cases and prevalence by SDI regions. (B) The percentage change in cases between 1990 and 2019. (C) The prevalence rates in 2019. (D) The EAPC of prevalence rates from 1990 to 2019. EAPC, estimated annual percentage change.

Supplementary Figure S2. Correlation of EAPC, prevalence, and SDI of anemia impairment in adolescents and young adults with HIV/AIDS.

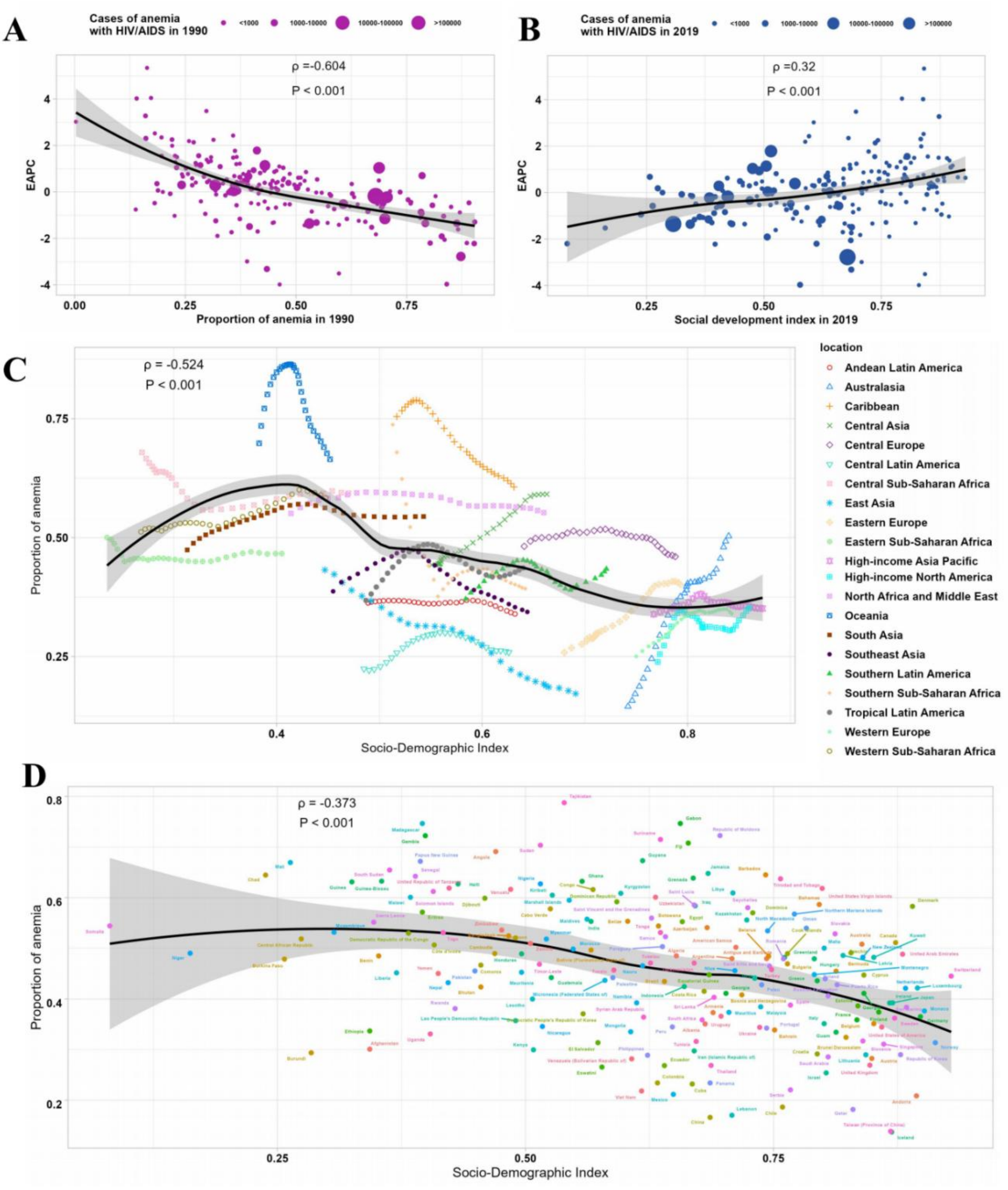

(A) The correlation between EAPC and the proportion of anemia in 1990. (B) The correlation between EAPC and SDI in 2019. (C) The proportion of anemia impairment in HIV/AIDS by regions from 1990 to 2019 based on SDI levels. (D) The proportion of anemia impairment in HIV/AIDS by nations from 1990 to 2019 is based on SDI levels. EAPC, estimated annual percentage change; SDI, Socio-Demographic Index.

Supplementary Table S1. The prevalence percentage and their change trends of anemia impairment in adolescents and young adults with HIV/AIDS for all genders at national level from 1990 to 2019.

Location	Severity	Cases, n (1990)	Cases, n (2019)	Changed, %	Percent, % (1990)	Percent, % (2019)	EAPC
**Afghanistan**	**Anemia**	30 (2 to 135)	630 (16 to 3732)	20.26 (0.06 to 129.61)	31.96 (15.38 to 57.42)	30.09 (9.11 to 59.11)	-0.11 (-0.25 to 0.03)
**Afghanistan**	Mild anemia	15 (1 to 66)	375 (10 to 2331)	23.83 (0.26 to 151.88)	16.54 (8.95 to 27.31)	17.9 (6 to 34.69)	0.39 (0.19 to 0.6)
**Afghanistan**	Moderat e anemia	13 (1 to 59)	236 (5 to 1443)	16.82 (-0.23 to 121.79)	14.09 (5.54 to 27.38)	11.33 (2.56 to 24.16)	-0.65 (-0.75 to -0.56)
**Afghanistan**	Severe anemia	1 (0 to 6)	18 (0 to 116)	13.59 (-0.48 to 108.34)	1.32 (0.43 to 2.91)	0.86 (0.18 to 2.08)	-1.5 (-1.62 to -1.37)
**Albania**	**Anemia**	2 (2 to 3)	2 (1 to 4)	0.12 (-0.24 to 0.64)	51.94 (47.85 to 55.66)	35.06 (32.14 to 38.64)	-1.44 (-1.87 to -1.01)
**Albania**	Mild anemia	1 (1 to 2)	2 (1 to 3)	0.3 (-0.14 to 0.91)	33.44 (29.05 to 37.67)	26.08 (23.01 to 29.33)	-0.93 (-1.35 to -0.5)
**Albania**	Moderat e anemia	1 (1 to 1)	1 (0 to 1)	-0.19 (-0.53 to 0.31)	17.78 (13.72 to 21.14)	8.7 (6.53 to 11.28)	-2.59 (-3.07 to -2.11)
**Albania**	Severe anemia	0 (0 to 0)	0 (0 to 0)	-0.35 (-0.69 to 0.41)	0.72 (0.4 to 1.17)	0.28 (0.18 to 0.43)	-3.25 (-3.49 to -3)
**Algeria**	**Anemia**	76 (30 to 324)	452 (66 to 1848)	4.93 (0.21 to 16.87)	57.24 (45.71 to 72.42)	48.52 (29.65 to 73.45)	-0.61 (-0.71 to -0.52)
**Algeria**	Mild anemia	42 (16 to 179)	294 (43 to 1114)	5.99 (0.39 to 21.05)	31.54 (24.26 to 40.21)	31.62 (18.78 to 49.32)	0.02 (-0.12 to 0.16)
**Algeria**	Moderat e anemia	32 (12 to 138)	152 (20 to 654)	3.7 (-0.11 to 13.9)	24.34 (18.01 to 32.41)	16.3 (7.81 to 28.68)	-1.48 (-1.65 to -1.31)
**Algeria**	Severe anemia	2 (0 to 7)	6 (1 to 22)	2.13 (-0.52 to 12.07)	1.36 (0.61 to 2.56)	0.6 (0.22 to 1.37)	-2.83 (-3.02 to -2.64)
**American Samoa**	**Anemia**	0 (0 to 1)	1 (1 to 2)	4.68 (1.58 to 12.98)	39.35 (31.76 to 48.51)	50.2 (43.87 to 55.6)	0.87 (0.31 to 1.42)

**American Samoa**	Mild anemia	0 (0 to 0)	1 (0 to 1)	5.17 (1.72 to 14.47)	23.25 (15.87 to 30.22)	32.17 (26.63 to 37.72)	1.31 (0.85 to 1.78)
**American Samoa**	Moderate anemia	0 (0 to 0)	0 (0 to 1)	4.65 (1.48 to 12.91)	13.55 (9.3 to 18.19)	17.29 (13.21 to 21.88)	0.97 (0.19 to 1.76)
**American Samoa**	Severe anemia	0 (0 to 0)	0 (0 to 0)	0.31 (-0.8 to 11.97)	2.55 (0.4 to 10.09)	0.74 (0.33 to 1.42)	-6.96 (-8.33 to -5.56)
**Andorra**	**Anemia**	1 (0 to 4)	1 (0 to 3)	-0.08 (-0.84 to 6.7)	22.57 (9.38 to 45.3)	20.87 (3.11 to 46.25)	-1.03 (-1.56 to -0.5)
**Andorra**	Mild anemia	1 (0 to 3)	1 (0 to 3)	-0.07 (-0.84 to 7.67)	18.79 (6.8 to 38.63)	17.55 (2.71 to 39.32)	-1 (-1.53 to -0.46)
**Andorra**	Moderate anemia	0 (0 to 1)	0 (0 to 0)	-0.14 (-0.88 to 7.71)	3.73 (1.19 to 9.59)	3.27 (0.38 to 7.68)	-1.21 (-1.72 to -0.69)
**Andorra**	Severe anemia	0 (0 to 0)	0 (0 to 0)	-0.14 (-0.93 to 12.02)	0.05 (0.01 to 0.16)	0.04 (0 to 0.14)	-1.39 (-1.81 to -0.98)
**Angola**	**Anemia**	1128 (423 to 2429)	32955 (18028 to 52932)	28.2 (10.45 to 84.47)	75.31 (60.56 to 89.65)	69.1 (53.41 to 81.01)	-0.34 (-0.37 to -0.31)
**Angola**	Mild anemia	501 (185 to 1091)	16668 (9175 to 27273)	32.26 (12.09 to 99.01)	33.4 (27.22 to 40.38)	34.94 (28.06 to 41.78)	0.21 (0.13 to 0.3)
**Angola**	Moderate anemia	582 (219 to 1270)	15470 (7938 to 25144)	25.59 (9.05 to 80.02)	38.85 (29.06 to 49.03)	32.45 (22.81 to 40.71)	-0.75 (-0.8 to -0.69)
**Angola**	Severe anemia	46 (13 to 107)	818 (337 to 1591)	16.92 (4.66 to 64.66)	3.06 (1.45 to 5.42)	1.71 (0.86 to 2.96)	-2.16 (-2.25 to -2.07)
**Antigua and Barbuda**	**Anemia**	4 (2 to 6)	14 (9 to 20)	2.61 (1.68 to 3.94)	41.67 (36.75 to 47.18)	47.97 (43.67 to 53.12)	0.31 (0.1 to 0.52)
**Antigua and Barbuda**	Mild anemia	2 (1 to 4)	9 (6 to 13)	2.64 (1.55 to 4.3)	26.08 (21.22 to 31.14)	30.3 (24.42 to 36.68)	0.44 (0.25 to 0.62)
**Antigua and Barbuda**	Moderate anemia	1 (1 to 2)	5 (3 to 8)	2.58 (1.38 to 4.25)	14.91 (11.66 to 17.92)	17.06 (12.33 to 21.17)	0.12 (-0.14 to 0.38)
**Antigua and Barbuda**	Severe anemia	0 (0 to 0)	0 (0 to 0)	1.85 (0.08 to 6.06)	0.68 (0.33 to 1.17)	0.61 (0.25 to 1.3)	-0.33 (-0.86 to 0.21)
**Argentina**	**Anemia**	4028 (2296 to	7662 (3973 to	0.9 (0.36 to	39.92 (34.29 to	47.97 (40.62 to	0.14 (-0.1 to

		6173)	12768)	1.61)	45.76)	54.65)	0.38)
**Argentina**	Mild anemia	2762 (1585 to 4236)	5772 (2995 to 9758)	1.09 (0.49 to 1.94)	27.37 (23.31 to 32.17)	36.1 (30.65 to 42.22)	0.45 (0.18 to 0.72)
**Argentina**	Moderate anemia	1221 (667 to 1939)	1847 (914 to 3201)	0.51 (-0.05 to 1.28)	12.1 (9.8 to 14.71)	11.6 (8.18 to 15.05)	-0.62 (-0.81 to -0.43)
**Argentina**	Severe anemia	45 (22 to 77)	43 (15 to 91)	-0.03 (-0.61 to 1.05)	0.45 (0.29 to 0.65)	0.27 (0.12 to 0.49)	-2.04 (-2.23 to -1.84)
**Armenia**	**Anemia**	1 (0 to 2)	10 (5 to 16)	5.91 (0 to 8.91)	41.49 (0 to 51.84)	37.31 (23.29 to 43.72)	-0.52 (-0.7 to -0.35)
**Armenia**	Mild anemia	1 (0 to 1)	7 (3 to 11)	6.46 (0 to 9.6)	26.8 (0 to 34.38)	26.02 (17.41 to 31.03)	-0.29 (-0.47 to -0.12)
**Armenia**	Moderate anemia	0 (0 to 1)	3 (1 to 5)	4.98 (0 to 9.14)	13.94 (0 to 19.78)	10.87 (5.2 to 15.25)	-0.94 (-1.13 to -0.75)
**Armenia**	Severe anemia	0 (0 to 0)	0 (0 to 0)	3.3 (0 to 8.37)	0.74 (0 to 1.32)	0.42 (0.18 to 0.76)	-2.33 (-2.53 to -2.12)
**Australia**	**Anemia**	113 (58 to 192)	240 (135 to 393)	1.12 (0.34 to 2.21)	14 (9.75 to 20.7)	50.82 (42.3 to 58.73)	4.02 (3.43 to 4.62)
**Australia**	Mild anemia	91 (47 to 158)	193 (107 to 319)	1.12 (0.29 to 2.33)	11.31 (7.61 to 17.59)	40.95 (33.36 to 48.15)	3.99 (3.4 to 4.59)
**Australia**	Moderate anemia	21 (11 to 37)	46 (24 to 80)	1.15 (0.37 to 2.61)	2.64 (1.63 to 3.85)	9.71 (7.16 to 12.74)	4.12 (3.47 to 4.76)
**Australia**	Severe anemia	0 (0 to 1)	1 (0 to 2)	0.69 (-0.45 to 4.17)	0.06 (0.02 to 0.12)	0.16 (0.06 to 0.35)	4.96 (3.92 to 6.01)
**Austria**	**Anemia**	73 (0 to 150)	111 (73 to 161)	0.51 (-0.16 to 3.74)	22.67 (0 to 34.98)	28.25 (24.56 to 32.89)	1.55 (1.16 to 1.95)
**Austria**	Mild anemia	61 (0 to 123)	92 (59 to 135)	0.52 (-0.18 to 3.6)	18.81 (0 to 29.4)	23.58 (19.75 to 28.16)	1.46 (1.13 to 1.79)
**Austria**	Moderate anemia	12 (0 to 28)	18 (11 to 28)	0.48 (-0.33 to 4.24)	3.8 (0 to 7.61)	4.62 (3.07 to 6.2)	1.97 (1.24 to 2.71)
**Austria**	Severe anemia	0 (0 to 1)	0 (0 to 1)	0.27 (-0.75 to 8.24)	0.06 (0 to 0.18)	0.06 (0.01 to 0.15)	1.59 (0.73 to 2.47)
**Azerbaijan**	**Anemia**	24	79	2.25	36.51	54.09	1.2

		(16 to 37)	(54 to 109)	(1.31 to 3.46)	(32.73 to 40.29)	(50.56 to 57.31)	(1.06 to 1.33)
**Azerbaijan**	Mild anemia	14 (9 to 21)	44 (29 to 62)	2.23 (1.22 to 3.63)	20.53 (17.83 to 23.47)	30.29 (26.2 to 35.01)	1.27 (1.19 to 1.36)
**Azerbaijan**	Moderate anemia	10 (6 to 15)	33 (22 to 47)	2.37 (1.36 to 3.74)	14.87 (12.93 to 17.1)	22.83 (19.5 to 25.71)	1.23 (1.01 to 1.44)
**Azerbaijan**	Severe anemia	1 (0 to 1)	1 (1 to 3)	0.9 (-0.21 to 3.4)	1.11 (0.7 to 1.72)	0.96 (0.46 to 1.82)	-1.12 (-1.42 to -0.81)
**Bahamas**	**Anemia**	123 (67 to 204)	188 (115 to 295)	0.53 (0.18 to 1.03)	50.52 (45.3 to 55.75)	58.66 (54.65 to 62.68)	0.61 (0.35 to 0.87)
**Bahamas**	Mild anemia	73 (39 to 126)	120 (67 to 200)	0.64 (0.1 to 1.36)	30.1 (24.72 to 35.14)	37.44 (27.61 to 46.15)	0.75 (0.5 to 1.01)
**Bahamas**	Moderate anemia	47 (25 to 81)	65 (34 to 119)	0.38 (-0.2 to 1.26)	19.48 (15.15 to 23.36)	20.37 (12.57 to 28.99)	0.4 (0.11 to 0.7)
**Bahamas**	Severe anemia	2 (1 to 5)	3 (1 to 6)	0.18 (-0.55 to 2.21)	0.94 (0.44 to 1.75)	0.84 (0.34 to 1.81)	-0.19 (-0.4 to 0.01)
**Bahrain**	**Anemia**	2 (1 to 3)	5 (3 to 6)	1.56 (0.86 to 2.61)	31.81 (26.96 to 37.56)	33.87 (29.87 to 37.56)	0.06 (-0.37 to 0.48)
**Bahrain**	Mild anemia	1 (1 to 2)	4 (2 to 5)	2.57 (1.37 to 4.3)	16.88 (13.02 to 21.42)	25.02 (20 to 29.15)	1.46 (1.18 to 1.75)
**Bahrain**	Moderate anemia	1 (1 to 1)	1 (1 to 2)	0.45 (-0.08 to 1.41)	14.42 (12.06 to 16.78)	8.68 (5.94 to 13.04)	-1.97 (-2.68 to -1.25)
**Bahrain**	Severe anemia	0 (0 to 0)	0 (0 to 0)	-0.21 (-0.72 to 1.28)	0.52 (0.26 to 0.94)	0.17 (0.08 to 0.33)	-3.73 (-4.12 to -3.34)
**Bangladesh**	**Anemia**	NA	686 (276 to 2454)	NA	NA	52.48 (39.14 to 68.21)	NA
**Bangladesh**	Mild anemia	NA	408 (157 to 1427)	NA	NA	31.15 (22.9 to 41.14)	NA
**Bangladesh**	Moderate anemia	NA	268 (100 to 990)	NA	NA	20.53 (13.72 to 28.18)	NA
**Bangladesh**	Severe anemia	NA	10 (3 to 37)	NA	NA	0.79 (0.37 to 1.46)	NA

**Barbados**	**Anemia**	18 (10 to 30)	55 (39 to 80)	2.05 (1.13 to 3.39)	31.35 (27.09 to 36.57)	64.51 (60.39 to 69.12)	2.24 (1.88 to 2.61)
**Barbados**	Mild anemia	13 (7 to 22)	40 (26 to 61)	2.06 (1.01 to 3.62)	22.49 (18.42 to 27.2)	46.42 (37.66 to 55.34)	2.25 (1.88 to 2.61)
**Barbados**	Moderate anemia	5 (3 to 9)	15 (8 to 26)	2.04 (0.54 to 4.4)	8.63 (6.7 to 10.72)	17.69 (9.87 to 25.26)	2.27 (1.89 to 2.65)
**Barbados**	Severe anemia	0 (0 to 0)	0 (0 to 1)	1.54 (-0.2 to 5.7)	0.24 (0.13 to 0.38)	0.4 (0.13 to 0.91)	0.95 (0.48 to 1.43)
**Belarus**	**Anemia**	21 (0 to 41)	118 (49 to 194)	4.54 (0 to 11.1)	42.86 (0 to 55.78)	48.44 (24.4 to 57.8)	0.91 (0.43 to 1.39)
**Belarus**	Mild anemia	15 (0 to 29)	89 (38 to 142)	4.91 (0 to 11.45)	30.13 (0 to 39.23)	36.39 (19.53 to 45.06)	1.15 (0.68 to 1.63)
**Belarus**	Moderate anemia	6 (0 to 12)	29 (10 to 55)	3.72 (0 to 11.68)	12.25 (0 to 18.83)	11.74 (4.64 to 18.6)	0.33 (-0.16 to 0.82)
**Belarus**	Severe anemia	0 (0 to 1)	1 (0 to 2)	2.1 (-0.27 to 10.16)	0.49 (0 to 1.05)	0.3 (0.09 to 0.71)	-1.34 (-1.7 to -0.97)
**Belgium**	**Anemia**	87 (36 to 159)	218 (98 to 330)	1.5 (0.1 to 4.68)	33.51 (18.74 to 46.48)	35.11 (14.87 to 43.27)	0.37 (0.08 to 0.66)
**Belgium**	Mild anemia	73 (30 to 131)	181 (82 to 273)	1.49 (0.1 to 4.55)	27.89 (15.91 to 38.5)	29.07 (12.53 to 35.97)	0.34 (0.06 to 0.63)
**Belgium**	Moderate anemia	14 (5 to 29)	37 (16 to 63)	1.58 (0.06 to 6.09)	5.54 (2.5 to 9.34)	5.97 (2.42 to 8.46)	0.5 (0.17 to 0.83)
**Belgium**	Severe anemia	0 (0 to 1)	0 (0 to 1)	1.32 (-0.61 to 13.04)	0.08 (0.01 to 0.23)	0.07 (0.01 to 0.2)	0.15 (-0.1 to 0.4)
**Belize**	**Anemia**	29 (19 to 45)	200 (109 to 304)	5.77 (4.1 to 7.65)	49.6 (45.07 to 53.85)	55.35 (51.88 to 58.86)	0.31 (0.22 to 0.41)
**Belize**	Mild anemia	17 (11 to 26)	117 (60 to 187)	5.85 (3.78 to 8.32)	28.62 (24.28 to 33.2)	32.29 (26.53 to 38.25)	0.33 (0.24 to 0.43)
**Belize**	Moderate anemia	12 (7 to 18)	80 (42 to 127)	5.73 (3.71 to 8.31)	19.88 (16.28 to 23.09)	22.06 (17.72 to 26.46)	0.32 (0.23 to 0.41)
**Belize**	Severe anemia	1 (0 to 1)	4 (1 to 8)	4.5 (1.22 to	1.11 (0.57 to	1 (0.46 to	-0.44 (-0.53 to

				11.86)	1.85)	1.93)	-0.35)
**Benin**	**Anemia**	298 (174 to 478)	4340 (2858 to 6382)	13.55 (8 to 22.55)	61.48 (50.28 to 72.11)	48.5 (39.94 to 57.99)	-1.16 (-1.36 to -0.97)
**Benin**	Mild anemia	134 (80 to 210)	2011 (1289 to 2974)	14.01 (8.21 to 23.39)	27.63 (22.96 to 32.61)	22.46 (18.25 to 27.26)	-0.94 (-1.19 to -0.7)
**Benin**	Moderat e anemia	153 (84 to 255)	2210 (1395 to 3335)	13.41 (7.45 to 23.55)	31.61 (23.31 to 40)	24.7 (18.81 to 30.67)	-1.27 (-1.47 to -1.06)
**Benin**	Severe anemia	11 (5 to 20)	119 (61 to 201)	9.93 (3.67 to 23.57)	2.24 (1.23 to 3.5)	1.33 (0.75 to 2.17)	-2.35 (-2.82 to -1.88)
**Bermuda**	**Anemia**	4 (2 to 6)	5 (3 to 8)	0.32 (-0.2 to 0.84)	38.18 (33.08 to 43.85)	45.49 (28.8 to 52.23)	0.42 (0.12 to 0.71)
**Bermuda**	Mild anemia	3 (2 to 4)	4 (3 to 6)	0.47 (-0.1 to 1.16)	25.68 (20.57 to 31.22)	34.12 (22.72 to 40.78)	0.84 (0.58 to 1.11)
**Bermuda**	Moderat e anemia	1 (1 to 2)	1 (1 to 2)	0.02 (-0.49 to 0.66)	12.06 (9.33 to 15.07)	11.09 (5.46 to 16.22)	-0.58 (-0.96 to -0.2)
**Bermuda**	Severe anemia	0 (0 to 0)	0 (0 to 0)	-0.3 (-0.78 to 1.01)	0.44 (0.21 to 0.83)	0.28 (0.09 to 0.6)	-1.88 (-2.15 to -1.61)
**Bhutan**	**Anemia**	9 (1 to 58)	46 (2 to 218)	4.21 (-0.5 to 50.17)	36.86 (24.3 to 56.64)	42.37 (21.06 to 69.33)	0.31 (0.25 to 0.37)
**Bhutan**	Mild anemia	4 (0 to 24)	24 (1 to 117)	5.17 (-0.42 to 60.59)	16.36 (11.73 to 23.44)	22.53 (12.56 to 35.99)	1.13 (0.99 to 1.26)
**Bhutan**	Moderat e anemia	4 (0 to 29)	20 (1 to 109)	3.68 (-0.6 to 43.26)	18.35 (10.78 to 30.07)	18.83 (7.62 to 33.74)	-0.28 (-0.44 to -0.11)
**Bhutan**	Severe anemia	0 (0 to 3)	1 (0 to 6)	1.17 (-0.84 to 21.09)	2.15 (0.9 to 4.31)	1.01 (0.36 to 2.26)	-2.96 (-3.09 to -2.82)
**Bolivia (Plurinational State of)**	**Anemia**	348 (93 to 1343)	1201 (155 to 4323)	2.45 (-0.44 to 11.94)	44.22 (34.68 to 55.29)	49.67 (35.77 to 67.51)	0.33 (0.27 to 0.39)
**Bolivia (Plurinational State of)**	Mild anemia	165 (43 to 657)	721 (89 to 2476)	3.38 (-0.31 to 16.26)	20.89 (15.91 to 26.88)	29.81 (21.63 to 41.3)	1.17 (1.1 to 1.23)
**Bolivia (Plurinational State of)**	Moderat e anemia	166 (45 to 643)	449 (58 to 1555)	1.71 (-0.56 to 8.95)	21.1 (15.66 to 27.13)	18.62 (12.19 to 26.06)	-0.49 (-0.58 to -0.4)
**Bolivia**	Severe	17	30	0.74	2.23	1.25	-2.04

**(Plurinational State of)**	anemia	(4 to 66)	(4 to 104)	(-0.74 to 6.37)	(1.34 to 3.37)	(0.71 to 2.02)	(-2.12 to -1.96)
**Bosnia and Herzegovina**	**Anemia**	3 (3 to 4)	4 (2 to 5)	0.1 (-0.3 to 0.55)	31.7 (26.13 to 37.13)	40.82 (34.37 to 47.67)	0.14 (-0.16 to 0.43)
**Bosnia and Herzegovina**	Mild anemia	2 (2 to 3)	3 (1 to 4)	0.14 (-0.33 to 0.68)	22.45 (18.24 to 26.73)	29.93 (20.12 to 36.01)	0.44 (0.22 to 0.66)
**Bosnia and Herzegovina**	Moderat e anemia	1 (1 to 1)	1 (0 to 1)	-0.04 (-0.47 to 0.53)	8.81 (6.66 to 11.65)	9.98 (6.12 to 13.01)	-0.67 (-1.13 to -0.21)
**Bosnia and Herzegovina**	Severe anemia	0 (0 to 0)	0 (0 to 1)	0.72 (-0.73 to 23.53)	0.44 (0.24 to 0.71)	0.91 (0.17 to 14.8)	-0.5 (-1.78 to 0.8)
**Botswana**	**Anemia**	10150 (7626 to 12717)	11786 (7297 to 16179)	0.16 (-0.3 to 0.74)	78.47 (69.92 to 84.21)	54.41 (29.57 to 67.98)	0.7 (-0.44 to 1.86)
**Botswana**	Mild anemia	4925 (3585 to 6421)	7556 (4959 to 10463)	0.53 (-0.07 to 1.4)	38.01 (33.06 to 44.38)	34.62 (21.2 to 42.76)	1.81 (0.62 to 3.01)
**Botswana**	Moderat e anemia	4871 (3598 to 6153)	4025 (1578 to 6372)	-0.17 (-0.7 to 0.44)	37.72 (31.05 to 42.72)	18.83 (5.91 to 29.44)	-0.62 (-1.91 to 0.7)
**Botswana**	Severe anemia	353 (237 to 491)	204 (64 to 399)	-0.42 (-0.84 to 0.31)	2.74 (1.94 to 3.71)	0.96 (0.23 to 1.96)	-3.58 (-4.38 to -2.78)
**Brazil**	**Anemia**	16587 (10304 to 24750)	30205 (19363 to 41678)	0.82 (0.44 to 1.33)	36.74 (32.88 to 41.09)	43.46 (40.8 to 46.7)	0.05 (-0.26 to 0.37)
**Brazil**	Mild anemia	8860 (5323 to 13199)	17439 (11171 to 25016)	0.97 (0.49 to 1.67)	19.65 (16.07 to 23.38)	25.09 (21.44 to 28.93)	0.39 (0.07 to 0.71)
**Brazil**	Moderat e anemia	7042 (4204 to 10935)	11992 (7311 to 17075)	0.7 (0.28 to 1.3)	15.57 (13.33 to 17.9)	17.25 (14.45 to 20.11)	-0.28 (-0.6 to 0.04)
**Brazil**	Severe anemia	685 (305 to 1279)	774 (356 to 1434)	0.13 (-0.45 to 1.48)	1.51 (0.83 to 2.5)	1.11 (0.61 to 1.86)	-1.54 (-1.78 to -1.29)
**Brunei Darussalam**	**Anemia**	2 (1 to 3)	23 (13 to 37)	11.3 (7.74 to 17.47)	45 (38.66 to 51.61)	32.42 (27.33 to 37.77)	-1.31 (-1.43 to -1.2)
**Brunei Darussalam**	Mild anemia	1 (1 to 2)	16 (9 to 26)	12.9 (8.49 to 20.4)	27.28 (22.02 to 32.33)	22.2 (17.93 to 26.9)	-0.87 (-1.02 to -0.72)
**Brunei Darussalam**	Moderat e anemia	1 (0 to 1)	7 (4 to 12)	9 (5.51 to 15.51)	16.71 (12.96 to 21.01)	9.81 (7.41 to 12.82)	-2.11 (-2.21 to -2.02)

**Brunei Darussalam**	Severe anemia	0 (0 to 0)	0 (0 to 1)	6.13 (2.34 to 15.94)	1.01 (0.55 to 1.77)	0.42 (0.23 to 0.73)	-2.54 (-2.86 to -2.23)
**Bulgaria**	**Anemia**	17 (0 to 29)	84 (49 to 128)	3.93 (0 to 6.61)	38.24 (0 to 46.78)	46.94 (39.55 to 52.63)	0.51 (0.42 to 0.6)
**Bulgaria**	Mild anemia	12 (0 to 20)	60 (35 to 94)	4.07 (0 to 7.17)	26.83 (0 to 33.01)	33.92 (27.63 to 39.72)	0.71 (0.63 to 0.79)
**Bulgaria**	Moderate anemia	5 (0 to 9)	22 (12 to 37)	3.62 (0 to 7.47)	10.94 (0 to 14.73)	12.59 (8.6 to 16.23)	0.51 (0.38 to 0.65)
**Bulgaria**	Severe anemia	0 (0 to 0)	1 (0 to 1)	2.65 (0 to 8.27)	0.46 (0 to 0.83)	0.42 (0.21 to 0.74)	-5.35 (-7.19 to -3.47)
**Burkina Faso**	**Anemia**	16065 (11240 to 22085)	6768 (4601 to 9259)	-0.58 (-0.71 to -0.39)	39.21 (31.03 to 47.7)	47.88 (36.13 to 57.54)	0.68 (0.25 to 1.11)
**Burkina Faso**	Mild anemia	7641 (5335 to 10371)	2848 (1973 to 3950)	-0.63 (-0.74 to -0.46)	18.61 (15.65 to 22.03)	20.13 (15.55 to 24.77)	0.4 (-0.22 to 1.02)
**Burkina Faso**	Moderate anemia	7949 (4810 to 11599)	3638 (2201 to 5140)	-0.54 (-0.74 to -0.21)	19.44 (12.55 to 26.06)	25.74 (16.21 to 32.55)	0.8 (0.49 to 1.11)
**Burkina Faso**	Severe anemia	476 (221 to 885)	283 (141 to 484)	-0.41 (-0.74 to 0.44)	1.16 (0.58 to 2.11)	2 (1.04 to 3.28)	2.39 (1.81 to 2.98)
**Burundi**	**Anemia**	9246 (2358 to 17605)	4238 (2725 to 5960)	-0.54 (-0.78 to 0.73)	35.13 (22.17 to 50.83)	29.37 (19.83 to 36.38)	0 (-0.34 to 0.34)
**Burundi**	Mild anemia	4804 (1203 to 9435)	2403 (1594 to 3380)	-0.5 (-0.76 to 1.02)	18.02 (11.94 to 27.11)	16.66 (11.97 to 21.38)	0.45 (0.02 to 0.89)
**Burundi**	Moderate anemia	4107 (1067 to 8095)	1730 (970 to 2827)	-0.58 (-0.83 to 0.69)	15.81 (7.9 to 24.69)	12 (7 to 18.07)	-0.42 (-0.72 to -0.12)
**Burundi**	Severe anemia	335 (72 to 847)	104 (51 to 192)	-0.69 (-0.9 to 0.65)	1.3 (0.44 to 2.87)	0.72 (0.38 to 1.27)	-1.81 (-2.18 to -1.43)
**Cabo Verde**	**Anemia**	140 (51 to 279)	144 (63 to 296)	0.03 (-0.54 to 1.95)	86.6 (78.58 to 91.47)	57.8 (50.51 to 67.96)	-1.61 (-1.73 to -1.5)
**Cabo Verde**	Mild anemia	76 (28 to 150)	88 (38 to 182)	0.17 (-0.49 to 2.53)	46.86 (39.05 to 54.78)	35.41 (29.29 to 43.53)	-1.1 (-1.23 to -0.98)
**Cabo Verde**	Moderate anemia	61 (22 to	54 (23 to	-0.12 (-0.63 to	37.69 (30.19 to	21.62 (16.6 to	-2.2 (-2.37 to

		126)	112)	1.54)	43.91)	27.36)	-2.04)
**Cabo Verde**	Severe anemia	3 (1 to 8)	2 (1 to 5)	-0.42 (-0.81 to 0.93)	2.05 (0.98 to 3.89)	0.77 (0.37 to 1.41)	-3.89 (-4.2 to -3.58)
**Cambodia**	**Anemia**	131 (72 to 212)	2348 (1272 to 3695)	16.99 (7.59 to 37.46)	35.56 (30.28 to 40.61)	48.97 (42.27 to 54.71)	0.29 (-0.19 to 0.78)
**Cambodia**	Mild anemia	69 (36 to 112)	1513 (790 to 2380)	20.97 (9.55 to 47.04)	18.76 (15.76 to 22)	31.63 (26.28 to 36.18)	1.26 (0.92 to 1.61)
**Cambodia**	Moderate anemia	57 (31 to 94)	800 (394 to 1382)	12.98 (5.03 to 31.84)	15.58 (12.85 to 18.48)	16.59 (12.75 to 21.42)	-0.81 (-1.51 to -0.11)
**Cambodia**	Severe anemia	4 (2 to 8)	36 (13 to 67)	7 (1.51 to 22.37)	1.22 (0.69 to 1.93)	0.75 (0.33 to 1.28)	-3.1 (-3.74 to -2.45)
**Cameroon**	**Anemia**	3776 (2432 to 5762)	40046 (28601 to 51859)	9.61 (5.35 to 16.71)	40.42 (32.27 to 48.64)	52.06 (40.15 to 63.85)	0.93 (0.79 to 1.07)
**Cameroon**	Mild anemia	2037 (1286 to 3103)	20804 (15656 to 26729)	9.21 (5.33 to 15.8)	21.8 (17.98 to 25.61)	27.04 (21.95 to 32.4)	0.71 (0.56 to 0.87)
**Cameroon**	Moderate anemia	1616 (967 to 2601)	18170 (11058 to 25129)	10.24 (4.83 to 19.67)	17.31 (12.53 to 22.78)	23.63 (15.09 to 31.05)	1.23 (0.98 to 1.48)
**Cameroon**	Severe anemia	123 (62 to 222)	1072 (528 to 1875)	7.73 (2.5 to 19.54)	1.31 (0.77 to 2.08)	1.39 (0.71 to 2.41)	0.13 (-0.34 to 0.61)
**Canada**	**Anemia**	606 (222 to 1211)	1055 (580 to 1664)	0.74 (0.01 to 2.69)	16 (10.48 to 22.35)	51.13 (37.83 to 60.54)	3.27 (2.59 to 3.96)
**Canada**	Mild anemia	476 (178 to 966)	832 (455 to 1307)	0.75 (-0.01 to 2.67)	12.57 (8.21 to 18.2)	40.36 (29.52 to 48.74)	3.32 (2.65 to 4)
**Canada**	Moderate anemia	127 (41 to 268)	218 (114 to 379)	0.72 (-0.07 to 2.9)	3.34 (1.94 to 5.11)	10.55 (7 to 14.49)	3.11 (2.39 to 3.84)
**Canada**	Severe anemia	3 (1 to 9)	5 (1 to 12)	0.47 (-0.59 to 4.42)	0.08 (0.02 to 0.19)	0.22 (0.07 to 0.49)	2.61 (1.87 to 3.34)
**Central African Republic**	**Anemia**	7773 (4114 to 11747)	7589 (3905 to 13010)	-0.02 (-0.49 to 0.79)	50.22 (33.35 to 64.94)	51.86 (35.61 to 65.86)	0.18 (-0.04 to 0.41)
**Central African Republic**	Mild anemia	3970 (2137 to 6074)	3686 (2027 to 6353)	-0.07 (-0.48 to 0.69)	25.58 (18.94 to 33.06)	25.12 (19.41 to 31.9)	0 (-0.19 to 0.2)
**Central African**	Moderat	3550	3652	0.03	23	25.02	0.38

**Republic**	e anemia	(1554 to 5779)	(1607 to 6534)	(-0.55 to 1.28)	(11.88 to 33.4)	(13.1 to 34.18)	(0.13 to 0.63)
**Central African Republic**	Severe anemia	253 (88 to 530)	251 (89 to 531)	-0.01 (-0.67 to 2.05)	1.65 (0.65 to 3.25)	1.73 (0.73 to 3.24)	0.19 (-0.15 to 0.54)
**Chad**	**Anemia**	3459 (1566 to 7582)	10423 (6383 to 15620)	2.01 (0.34 to 6.22)	78.39 (61.4 to 90.07)	64.47 (52.6 to 73.78)	-0.93 (-1.05 to -0.82)
**Chad**	Mild anemia	1568 (680 to 3394)	4706 (2876 to 6973)	2 (0.34 to 6.27)	35.48 (28.89 to 42.41)	29.13 (23.75 to 35.1)	-0.97 (-1.11 to -0.82)
**Chad**	Moderat e anemia	1739 (768 to 3835)	5298 (3141 to 8029)	2.05 (0.35 to 6.18)	39.46 (28.96 to 47.34)	32.76 (25.37 to 38.95)	-0.88 (-0.99 to -0.76)
**Chad**	Severe anemia	152 (55 to 363)	418 (193 to 807)	1.75 (-0.01 to 7.62)	3.46 (1.74 to 5.82)	2.58 (1.37 to 4.51)	-1.28 (-1.54 to -1.01)
**Chile**	**Anemia**	244 (139 to 402)	505 (219 to 938)	1.07 (0.09 to 2.43)	18.06 (12.87 to 23.46)	18.66 (10.87 to 26.07)	-0.48 (-0.83 to -0.14)
**Chile**	Mild anemia	185 (103 to 313)	404 (179 to 752)	1.18 (0.12 to 2.66)	13.75 (9.78 to 18.36)	14.9 (8.71 to 20.83)	-0.29 (-0.64 to 0.06)
**Chile**	Moderat e anemia	56 (29 to 93)	99 (41 to 200)	0.77 (-0.09 to 2.28)	4.15 (2.53 to 5.64)	3.67 (2.03 to 5.83)	-1.13 (-1.48 to -0.79)
**Chile**	Severe anemia	2 (1 to 4)	2 (1 to 5)	0.05 (-0.64 to 1.88)	0.16 (0.07 to 0.28)	0.09 (0.03 to 0.17)	-2.85 (-3.14 to -2.57)
**China**	**Anemia**	4782 (3021 to 6522)	6944 (3600 to 11889)	0.45 (-0.08 to 1.2)	43.42 (38.31 to 46.69)	16.61 (13.36 to 19.73)	-3.32 (-3.52 to -3.12)
**China**	Mild anemia	2768 (1735 to 3744)	5039 (2641 to 8503)	0.82 (0.04 to 1.79)	25.13 (21.81 to 28.48)	12.04 (10.02 to 13.99)	-2.49 (-2.72 to -2.25)
**China**	Moderat e anemia	1854 (1086 to 2641)	1713 (908 to 3051)	-0.08 (-0.46 to 0.53)	16.83 (12.39 to 20.68)	4.1 (2.96 to 5.27)	-4.89 (-5.08 to -4.69)
**China**	Severe anemia	160 (76 to 277)	193 (41 to 565)	0.2 (-0.77 to 3.04)	1.45 (0.78 to 2.31)	0.47 (0.12 to 1.47)	-5.8 (-6.46 to -5.14)
**Colombia**	**Anemia**	269 (128 to 547)	2420 (990 to 4373)	7.98 (3.24 to 13.97)	18.82 (15.07 to 23.67)	23.42 (11.63 to 31.04)	0.06 (-0.51 to 0.64)
**Colombia**	Mild anemia	198 (91 to 408)	1883 (798 to 3351)	8.51 (3.57 to 15.06)	13.84 (10.62 to 17.87)	18.23 (9.57 to 24.1)	0.27 (-0.31 to 0.85)

**Colombia**	Moderate anemia	69 (31 to 141)	522 (177 to 1049)	6.61 (2.1 to 13.76)	4.78 (3.44 to 6.25)	5.06 (2.11 to 7.7)	-0.54 (-1.12 to 0.04)
**Colombia**	Severe anemia	3 (1 to 7)	15 (3 to 38)	4.2 (0.33 to 13.64)	0.2 (0.1 to 0.36)	0.14 (0.04 to 0.33)	-1.49 (-1.87 to -1.11)
**Comoros**	**Anemia**	0 (0 to 1)	6 (3 to 22)	15.88 (7.3 to 56.45)	45.88 (39.77 to 49.98)	46.68 (32.16 to 58.56)	0.43 (0.23 to 0.62)
**Comoros**	Mild anemia	0 (0 to 0)	4 (2 to 13)	18.65 (8.47 to 67.5)	22.72 (18.62 to 26.59)	26.95 (19.9 to 34.12)	1 (0.83 to 1.18)
**Comoros**	Moderate anemia	0 (0 to 0)	3 (1 to 9)	13.52 (5.61 to 48.46)	21.22 (17.35 to 24.02)	18.56 (11.29 to 25.37)	-0.28 (-0.57 to 0.01)
**Comoros**	Severe anemia	0 (0 to 0)	0 (0 to 1)	9.12 (2.02 to 37.76)	1.94 (0.96 to 3.3)	1.17 (0.53 to 2.18)	-0.08 (-0.98 to 0.83)
**Congo**	**Anemia**	7609 (4037 to 12062)	7510 (4135 to 13044)	-0.01 (-0.48 to 0.99)	78.22 (64.25 to 91.44)	61.6 (45.37 to 72.76)	-0.87 (-0.95 to -0.79)
**Congo**	Mild anemia	3378 (1717 to 5657)	3795 (2083 to 6528)	0.12 (-0.42 to 1.41)	34.72 (27.99 to 42.5)	31.07 (23.43 to 39.67)	-0.47 (-0.64 to -0.31)
**Congo**	Moderate anemia	3974 (2031 to 6487)	3551 (1621 to 6384)	-0.11 (-0.62 to 0.82)	40.86 (29.5 to 51.2)	29.19 (15.53 to 37.02)	-1.15 (-1.26 to -1.03)
**Congo**	Severe anemia	256 (93 to 520)	164 (60 to 356)	-0.36 (-0.81 to 0.9)	2.64 (1.24 to 4.85)	1.35 (0.58 to 2.41)	-2.3 (-2.52 to -2.07)
**Cook Islands**	**Anemia**	0 (0 to 0)	2 (0 to 6)	18.74 (1.36 to 57.31)	38.08 (22.58 to 56.31)	48.96 (26.06 to 75.86)	0.77 (0.59 to 0.96)
**Cook Islands**	Mild anemia	0 (0 to 0)	1 (0 to 4)	20 (1.65 to 62.67)	24.6 (15.18 to 35.43)	33.93 (18.38 to 52.43)	1.1 (0.96 to 1.23)
**Cook Islands**	Moderate anemia	0 (0 to 0)	0 (0 to 2)	16.63 (1.19 to 52.97)	12.91 (6.23 to 21.74)	14.58 (6.47 to 24.96)	0.4 (0.23 to 0.57)
**Cook Islands**	Severe anemia	0 (0 to 0)	0 (0 to 0)	11.16 (0.35 to 49.82)	0.58 (0.22 to 1.21)	0.46 (0.15 to 1.03)	-3.74 (-5.93 to -1.5)
**Costa Rica**	**Anemia**	44 (27 to 73)	290 (151 to 466)	5.53 (3.65 to 7.88)	19.61 (16.37 to 23.73)	41.52 (33.99 to 45.96)	2.35 (1.98 to 2.71)
**Costa Rica**	Mild anemia	32 (18 to 52)	211 (111 to	5.7 (3.59 to	13.92 (10.96 to	30.2 (25.06 to	2.45 (2.1 to

			345)	8.57)	17.8)	34.48)	2.81)
**Costa Rica**	Moderate anemia	12 (7 to 20)	76 (37 to 127)	5.18 (3.05 to 8.06)	5.45 (4.17 to 6.7)	10.94 (7.61 to 14.24)	2.11 (1.7 to 2.52)
**Costa Rica**	Severe anemia	1 (0 to 1)	3 (1 to 6)	3.76 (0.85 to 9.96)	0.24 (0.12 to 0.42)	0.37 (0.16 to 0.7)	1.16 (0.82 to 1.51)
**Côte d'Ivoire**	**Anemia**	30567 (7870 to 64402)	27013 (19639 to 34771)	-0.12 (-0.57 to 2.34)	60.69 (44.53 to 74.68)	50.66 (40.21 to 57.61)	-0.52 (-0.82 to -0.22)
**Côte d'Ivoire**	Mild anemia	13422 (3211 to 28968)	11821 (8704 to 15635)	-0.12 (-0.59 to 2.34)	26.31 (20.75 to 32)	22.16 (18.18 to 25.75)	-0.6 (-0.89 to -0.31)
**Côte d'Ivoire**	Moderate anemia	16170 (4178 to 34111)	14216 (9239 to 18684)	-0.12 (-0.59 to 2.32)	32.4 (20.76 to 43.13)	26.67 (18.42 to 31.57)	-0.52 (-0.83 to -0.22)
**Côte d'Ivoire**	Severe anemia	975 (233 to 2244)	977 (533 to 1610)	0 (-0.62 to 3.22)	1.98 (0.96 to 3.62)	1.83 (1.02 to 2.88)	0.45 (0.01 to 0.89)
**Croatia**	**Anemia**	5 (2 to 7)	10 (7 to 14)	1.14 (0.29 to 2.33)	27.36 (18.26 to 35.34)	29.14 (23.35 to 35.54)	0.4 (0.15 to 0.66)
**Croatia**	Mild anemia	4 (2 to 6)	8 (5 to 11)	1.21 (0.33 to 2.41)	21.23 (14.32 to 27.37)	23.33 (18.29 to 29.08)	0.52 (0.29 to 0.76)
**Croatia**	Moderate anemia	1 (0 to 2)	2 (1 to 3)	0.92 (0.02 to 2.64)	5.92 (3.16 to 8.39)	5.67 (4.15 to 7.44)	0 (-0.32 to 0.33)
**Croatia**	Severe anemia	0 (0 to 0)	0 (0 to 0)	0.46 (-0.45 to 2.83)	0.2 (0.08 to 0.39)	0.15 (0.06 to 0.28)	-1.01 (-1.3 to -0.72)
**Cuba**	**Anemia**	134 (89 to 199)	249 (120 to 450)	0.86 (0.2 to 1.7)	41.33 (35.35 to 47.21)	23.21 (17.28 to 30.95)	-1.7 (-1.95 to -1.45)
**Cuba**	Mild anemia	89 (56 to 137)	184 (84 to 340)	1.06 (0.26 to 2.21)	27.44 (22.03 to 33.08)	17.1 (12.04 to 23.66)	-1.4 (-1.63 to -1.17)
**Cuba**	Moderate anemia	43 (27 to 65)	64 (29 to 117)	0.46 (-0.14 to 1.21)	13.38 (10.42 to 16.45)	5.94 (4.11 to 8.25)	-2.37 (-2.71 to -2.02)
**Cuba**	Severe anemia	2 (1 to 3)	2 (1 to 5)	0.17 (-0.52 to 1.86)	0.51 (0.25 to 0.91)	0.18 (0.08 to 0.36)	-2.61 (-3.71 to -1.5)
**Cyprus**	**Anemia**	0 (0 to 1)	11 (6 to 20)	29.86 (13.5 to 66.03)	16.35 (8.34 to 29.25)	44.72 (23.46 to 57.55)	5.34 (4.32 to 6.37)
**Cyprus**	Mild	0	10	30.65	13.18	37.09	5.26

	anemia	(0 to 1)	(5 to 16)	(13.03 to 87.52)	(4.91 to 24.68)	(19.28 to 48.46)	(4.33 to 6.18)
**Cyprus**	Moderate anemia	0 (0 to 0)	2 (1 to 4)	26.95 (8.4 to 92.4)	3.08 (0.97 to 7.65)	7.53 (3.77 to 10.62)	5.91 (4.31 to 7.53)
**Cyprus**	Severe anemia	0 (0 to 0)	0 (0 to 0)	11.46 (0.48 to 125.78)	0.09 (0.01 to 0.33)	0.1 (0.02 to 0.27)	3.37 (0.79 to 6.01)
**Czechia**	**Anemia**	4 (3 to 6)	36 (22 to 54)	7.02 (4.38 to 10.68)	37.38 (26.93 to 45.7)	49.11 (35.96 to 56.84)	0.65 (0.27 to 1.03)
**Czechia**	Mild anemia	3 (2 to 5)	27 (16 to 41)	7.22 (4.45 to 10.86)	27.68 (20.49 to 33.84)	37.3 (27.86 to 44.66)	0.4 (-0.14 to 0.95)
**Czechia**	Moderate anemia	1 (1 to 2)	8 (4 to 12)	6.02 (3.25 to 10.68)	9.33 (5.81 to 12.74)	10.72 (7.17 to 13.98)	1.01 (0.36 to 1.66)
**Czechia**	Severe anemia	0 (0 to 0)	1 (0 to 6)	16.5 (0.91 to 159.44)	0.38 (0.17 to 0.67)	1.09 (0.13 to 9.44)	4.56 (2.72 to 6.44)
**Democratic People's Republic of Korea**	**Anemia**	51 (14 to 250)	482 (78 to 2144)	8.4 (0.59 to 51.9)	45.47 (24.15 to 65.08)	37.07 (21.25 to 62.42)	-0.68 (-0.82 to -0.54)
**Democratic People's Republic of Korea**	Mild anemia	29 (8 to 133)	307 (47 to 1378)	9.66 (0.7 to 62.13)	25.62 (14.27 to 36.11)	23.53 (13.65 to 40.1)	-0.57 (-0.68 to -0.47)
**Democratic People's Republic of Korea**	Moderate anemia	21 (6 to 98)	166 (24 to 741)	6.88 (0.27 to 43.61)	18.66 (8.02 to 29.01)	12.86 (5.91 to 24.87)	-0.81 (-1.17 to -0.45)
**Democratic People's Republic of Korea**	Severe anemia	1 (0 to 6)	9 (1 to 42)	5.48 (0 to 39.71)	1.19 (0.45 to 2.45)	0.68 (0.26 to 1.42)	-1.27 (-1.96 to -0.58)
**Democratic Republic of the Congo**	**Anemia**	50460 (33543 to 71902)	26348 (17190 to 37482)	-0.48 (-0.63 to -0.26)	70.07 (60.74 to 79.04)	52.99 (42.27 to 61.21)	-1.15 (-1.42 to -0.88)
**Democratic Republic of the Congo**	Mild anemia	18184 (11799 to 27033)	12302 (7837 to 17894)	-0.32 (-0.55 to 0.03)	25.22 (21.11 to 30.1)	24.73 (19.13 to 29.16)	-0.3 (-0.73 to 0.13)
**Democratic Republic of the Congo**	Moderate anemia	28295 (18228 to 41408)	13122 (8426 to 18690)	-0.54 (-0.69 to -0.32)	39.3 (32.46 to 46.88)	26.4 (20.19 to 31.3)	-1.5 (-1.62 to -1.37)
**Democratic Republic of the Congo**	Severe anemia	3982 (1807 to 7445)	925 (503 to 1556)	-0.77 (-0.9 to -0.44)	5.55 (2.69 to 9.44)	1.86 (1.1 to 2.89)	-4.05 (-4.5 to -3.6)
**Denmark**	**Anemia**	66 (37 to 103)	125 (78 to 187)	0.89 (0.3 to 1.74)	35.81 (30.43 to 42.14)	58.29 (42.79 to 70.25)	1.48 (1.11 to 1.84)

**Denmark**	Mild anemia	57 (32 to 90)	112 (69 to 168)	0.94 (0.32 to 1.88)	31.22 (26.09 to 37.27)	52.11 (38.78 to 63.23)	1.58 (1.21 to 1.96)
**Denmark**	Moderate anemia	8 (4 to 14)	13 (7 to 23)	0.57 (-0.18 to 1.82)	4.57 (3.12 to 6.28)	6.16 (3.38 to 9.29)	0.68 (0.38 to 0.99)
**Denmark**	Severe anemia	0 (0 to 0)	0 (0 to 0)	0.46 (-0.79 to 4.08)	0.02 (0.01 to 0.03)	0.02 (0 to 0.07)	0.44 (0.12 to 0.76)
**Djibouti**	**Anemia**	12 (5 to 32)	715 (334 to 1314)	57.37 (18.58 to 182.88)	85.93 (79.62 to 90.43)	59.89 (49.64 to 69.1)	-1.36 (-1.64 to -1.08)
**Djibouti**	Mild anemia	6 (2 to 16)	392 (178 to 753)	65.7 (20.14 to 207.47)	41.25 (34.81 to 48.23)	32.79 (25.59 to 40.14)	-0.83 (-1.1 to -0.56)
**Djibouti**	Moderate anemia	6 (2 to 15)	305 (140 to 575)	50.69 (16.72 to 158.67)	41.37 (35.75 to 46.29)	25.61 (20.31 to 31.6)	-1.81 (-2.1 to -1.51)
**Djibouti**	Severe anemia	0 (0 to 1)	18 (7 to 38)	36.76 (9.67 to 148.47)	3.31 (1.74 to 5.57)	1.49 (0.78 to 2.6)	-3.11 (-3.57 to -2.64)
**Dominica**	**Anemia**	6 (4 to 9)	14 (8 to 20)	1.23 (0.72 to 1.92)	45.57 (40.16 to 50.65)	57.01 (52.9 to 61.34)	0.91 (0.68 to 1.15)
**Dominica**	Mild anemia	4 (2 to 6)	8 (4 to 12)	1.07 (0.52 to 1.87)	27.9 (23.28 to 32.85)	32.34 (26.86 to 38.26)	0.67 (0.43 to 0.91)
**Dominica**	Moderate anemia	2 (1 to 3)	6 (3 to 9)	1.5 (0.76 to 2.54)	16.85 (13.48 to 20.01)	23.57 (18.76 to 27.53)	1.26 (1.03 to 1.5)
**Dominica**	Severe anemia	0 (0 to 0)	0 (0 to 1)	1.38 (-0.12 to 4.88)	0.82 (0.42 to 1.42)	1.09 (0.43 to 2.3)	1.29 (0.99 to 1.59)
**Dominican Republic**	**Anemia**	2812 (1407 to 4917)	2358 (1147 to 4190)	-0.16 (-0.58 to 0.71)	69.38 (54.6 to 79.84)	59.19 (46.87 to 69.22)	-0.65 (-0.73 to -0.57)
**Dominican Republic**	Mild anemia	1495 (719 to 2663)	1393 (649 to 2452)	-0.07 (-0.56 to 0.99)	36.89 (29.31 to 44.42)	34.99 (27.18 to 42.14)	-0.35 (-0.46 to -0.24)
**Dominican Republic**	Moderate anemia	1240 (593 to 2159)	925 (431 to 1685)	-0.25 (-0.63 to 0.6)	30.58 (21.92 to 37.73)	23.2 (16.59 to 29.59)	-0.99 (-1.05 to -0.92)
**Dominican Republic**	Severe anemia	78 (29 to 170)	40 (14 to 91)	-0.48 (-0.83 to 0.46)	1.91 (0.93 to 3.37)	1.01 (0.45 to 1.98)	-2.04 (-2.2 to -1.88)
**Ecuador**	**Anemia**	222 (162 to	1609 (752 to	6.23 (2.98 to	34.04 (29.74 to	26.84 (18.46 to	-0.15 (-0.43 to

		324)	2828)	10.53)	38.9)	31.68)	0.13)
**Ecuador**	Mild anemia	137 (95 to 204)	1113 (527 to 1917)	7.11 (3.53 to 12.02)	20.98 (17.36 to 25.29)	18.59 (13.45 to 22.1)	0.16 (-0.07 to 0.39)
**Ecuador**	Moderate anemia	80 (56 to 117)	473 (209 to 870)	4.93 (1.93 to 9.32)	12.21 (9.56 to 14.43)	7.88 (4.95 to 10.14)	-0.68 (-1.04 to -0.31)
**Ecuador**	Severe anemia	6 (3 to 10)	22 (9 to 48)	3.04 (0.54 to 9.09)	0.85 (0.48 to 1.4)	0.37 (0.2 to 0.6)	-1.91 (-2.38 to -1.45)
**Egypt**	**Anemia**	110 (87 to 135)	471 (289 to 731)	3.27 (1.89 to 5.17)	57.59 (48.93 to 63.56)	55.27 (46.42 to 60.4)	-0.09 (-0.15 to -0.04)
**Egypt**	Mild anemia	58 (46 to 71)	338 (201 to 537)	4.83 (2.78 to 7.66)	30.23 (26.08 to 34.8)	39.57 (31.71 to 47.4)	0.99 (0.94 to 1.03)
**Egypt**	Moderate anemia	50 (37 to 63)	126 (57 to 215)	1.52 (0.19 to 3.12)	26.01 (20.48 to 30.3)	14.74 (8 to 20.54)	-2.24 (-2.36 to -2.11)
**Egypt**	Severe anemia	3 (1 to 4)	8 (2 to 71)	2.12 (-0.44 to 27.97)	1.34 (0.7 to 2.24)	0.96 (0.22 to 8.7)	2.42 (0.21 to 4.68)
**El Salvador**	**Anemia**	145 (72 to 257)	529 (269 to 808)	2.64 (0.9 to 4.76)	29.94 (25.06 to 34.92)	31.38 (18.59 to 38.72)	0.42 (0.2 to 0.65)
**El Salvador**	Mild anemia	94 (47 to 166)	373 (195 to 574)	2.98 (1.24 to 5.35)	19.32 (15.76 to 23.15)	22.11 (13.68 to 27.43)	0.72 (0.47 to 0.97)
**El Salvador**	Moderate anemia	48 (24 to 86)	149 (72 to 242)	2.1 (0.41 to 4.45)	9.89 (7.56 to 11.93)	8.84 (4.76 to 12.12)	-0.11 (-0.3 to 0.07)
**El Salvador**	Severe anemia	4 (2 to 7)	7 (3 to 15)	1 (-0.33 to 3.81)	0.74 (0.45 to 1.12)	0.43 (0.17 to 0.79)	-1.64 (-1.74 to -1.55)
**Equatorial Guinea**	**Anemia**	128 (55 to 262)	4215 (1850 to 7105)	32.02 (10.24 to 97.59)	83.55 (71.59 to 93.21)	44.78 (21.69 to 67.46)	-2.06 (-2.27 to -1.84)
**Equatorial Guinea**	Mild anemia	43 (19 to 92)	2734 (1203 to 4675)	61.99 (18.97 to 179.06)	28.45 (23.62 to 35.06)	28.85 (15.06 to 41.54)	0.35 (0.06 to 0.64)
**Equatorial Guinea**	Moderate anemia	75 (33 to 160)	1425 (559 to 3090)	17.92 (5.06 to 58.92)	49.21 (38.91 to 58.34)	15.33 (6.22 to 31.8)	-3.96 (-4.34 to -3.58)
**Equatorial Guinea**	Severe anemia	9 (3 to 21)	56 (20 to 151)	5.26 (0.5 to 25.15)	5.88 (2.51 to 10.09)	0.61 (0.22 to 1.5)	-7.92 (-8.43 to -7.4)
**Eritrea**	**Anemia**	2507	2018	-0.2	90.25	57.15	-2.22

		(727 to 6113)	(1278 to 3009)	(-0.66 to 1.69)	(85.61 to 93.52)	(51.5 to 63.7)	(-2.48 to -1.97)
**Eritrea**	Mild anemia	1065 (297 to 2649)	1027 (661 to 1526)	-0.04 (-0.6 to 2.3)	38.15 (32.22 to 44.64)	29.12 (24.35 to 34.47)	-1.78 (-2.11 to -1.45)
**Eritrea**	Moderate anemia	1301 (379 to 3051)	918 (542 to 1403)	-0.29 (-0.69 to 1.39)	46.96 (42.21 to 50.43)	25.96 (21.07 to 30.49)	-2.49 (-2.69 to -2.29)
**Eritrea**	Severe anemia	141 (36 to 363)	73 (31 to 131)	-0.48 (-0.83 to 1.2)	5.14 (2.78 to 8.36)	2.07 (1.09 to 3.59)	-3.78 (-3.99 to -3.56)
**Estonia**	**Anemia**	1 (0 to 1)	70 (32 to 105)	84.74 (31.06 to 137.3)	22.02 (14.17 to 28.78)	38.64 (17.44 to 47.69)	1.99 (1.41 to 2.57)
**Estonia**	Mild anemia	1 (0 to 1)	54 (25 to 82)	92.99 (33.86 to 157.22)	15.57 (9.3 to 21.11)	29.96 (13.56 to 37.6)	2.3 (1.71 to 2.89)
**Estonia**	Moderate anemia	0 (0 to 0)	15 (7 to 25)	77.07 (25.95 to 160.6)	5.29 (2.21 to 7.91)	8.46 (3.56 to 12.78)	1.43 (0.75 to 2.12)
**Estonia**	Severe anemia	0 (0 to 0)	0 (0 to 1)	8.52 (0 to 118.24)	1.17 (0.11 to 5.34)	0.22 (0.08 to 0.52)	-3.81 (-4.86 to -2.75)
**Eswatini**	**Anemia**	633 (394 to 1248)	6085 (3379 to 9053)	8.62 (3.08 to 18.02)	84.04 (74.79 to 87.48)	26.55 (13.11 to 43.77)	-3.97 (-5.1 to -2.83)
**Eswatini**	Mild anemia	322 (191 to 645)	4223 (2140 to 5992)	12.12 (3.81 to 24.72)	42.71 (37.17 to 48.83)	18.4 (7.85 to 27.46)	-3.09 (-4.46 to -1.71)
**Eswatini**	Moderate anemia	291 (174 to 559)	1765 (593 to 3356)	5.07 (0.68 to 14.44)	38.67 (31.38 to 43.65)	7.72 (2.36 to 16.32)	-5.26 (-6.12 to -4.4)
**Eswatini**	Severe anemia	20 (9 to 42)	98 (29 to 199)	3.89 (0.12 to 15.7)	2.67 (1.5 to 4.26)	0.43 (0.11 to 0.98)	-5.93 (-6.64 to -5.21)
**Ethiopia**	**Anemia**	42833 (24945 to 65512)	43718 (35117 to 54140)	0.02 (-0.31 to 0.66)	53.01 (43.36 to 62.81)	33.68 (29.69 to 37.8)	-1.36 (-1.46 to -1.26)
**Ethiopia**	Mild anemia	20107 (11827 to 30740)	24072 (19556 to 29669)	0.2 (-0.2 to 0.94)	24.85 (21.22 to 28.72)	18.54 (16.25 to 20.86)	-0.81 (-0.97 to -0.64)
**Ethiopia**	Moderate anemia	20358 (11966 to 31358)	18235 (14195 to 22974)	-0.1 (-0.39 to 0.45)	25.21 (19.69 to 30.81)	14.05 (12.03 to 16.14)	-1.78 (-1.93 to -1.64)
**Ethiopia**	Severe anemia	2369 (1341 to 4016)	1411 (1048 to 1895)	-0.4 (-0.65 to 0.05)	2.94 (2 to 4.16)	1.09 (0.86 to 1.4)	-3.45 (-3.63 to -3.27)

**Fiji**	**Anemia**	10 (6 to 16)	45 (29 to 66)	3.54 (2.24 to 6.67)	71.24 (66.6 to 75.03)	70.78 (66 to 75.65)	0.03 (-0.02 to 0.08)
**Fiji**	Mild anemia	6 (3 to 10)	27 (17 to 41)	3.67 (1.93 to 7.51)	41.57 (32.29 to 52.48)	42.53 (37.59 to 48.24)	0.11 (0.07 to 0.14)
**Fiji**	Moderat e anemia	4 (2 to 6)	17 (11 to 26)	3.91 (2.07 to 8.35)	25.31 (17.55 to 31.43)	27.23 (23.25 to 31.6)	0.16 (-0.04 to 0.36)
**Fiji**	Severe anemia	1 (0 to 2)	1 (0 to 1)	0.11 (-0.7 to 6.6)	4.36 (0.74 to 13.75)	1.02 (0.65 to 1.53)	-3.3 (-4.25 to -2.34)
**Finland**	**Anemia**	8 (4 to 12)	16 (10 to 24)	1.04 (0.42 to 2.33)	36.61 (22.68 to 44.35)	37.41 (28.94 to 46.37)	0.43 (0.26 to 0.61)
**Finland**	Mild anemia	6 (3 to 9)	13 (8 to 20)	1.16 (0.5 to 2.42)	28.33 (18.67 to 35.38)	30.69 (23.27 to 38.5)	0.62 (0.45 to 0.79)
**Finland**	Moderat e anemia	2 (1 to 3)	3 (2 to 4)	0.63 (-0.05 to 2.48)	8.09 (3.81 to 11.84)	6.61 (4.42 to 9.21)	-0.3 (-0.49 to -0.11)
**Finland**	Severe anemia	0 (0 to 0)	0 (0 to 0)	0.13 (-0.72 to 4.13)	0.19 (0.04 to 0.44)	0.1 (0.03 to 0.25)	-1.5 (-1.74 to -1.26)
**France**	**Anemia**	1215 (556 to 2244)	687 (303 to 1137)	-0.43 (-0.75 to 0.2)	16.08 (8.02 to 28.18)	35.88 (14.86 to 48.62)	2.3 (1.79 to 2.82)
**France**	Mild anemia	1030 (471 to 1919)	571 (255 to 940)	-0.44 (-0.76 to 0.18)	13.62 (6.76 to 23.94)	29.84 (12.52 to 41.01)	2.36 (1.86 to 2.86)
**France**	Moderat e anemia	183 (80 to 357)	114 (42 to 197)	-0.37 (-0.76 to 0.38)	2.43 (1.2 to 4.55)	5.98 (2.15 to 9)	1.96 (1.26 to 2.66)
**France**	Severe anemia	3 (1 to 6)	1 (0 to 3)	-0.59 (-0.93 to 0.62)	0.03 (0.01 to 0.09)	0.06 (0.01 to 0.15)	1.64 (0.83 to 2.45)
**Gabon**	**Anemia**	861 (279 to 2407)	2977 (1708 to 4936)	2.46 (0.09 to 11.49)	89.07 (77.44 to 94.52)	74.65 (61.02 to 82.93)	-0.47 (-0.58 to -0.36)
**Gabon**	Mild anemia	300 (89 to 890)	1390 (774 to 2450)	3.64 (0.3 to 17.71)	30.59 (24.52 to 37.75)	34.86 (27.18 to 48.99)	0.28 (0.16 to 0.4)
**Gabon**	Moderat e anemia	513 (163 to 1355)	1510 (696 to 2620)	1.94 (-0.12 to 10.29)	53.49 (43.05 to 61.51)	37.86 (20.6 to 47.39)	-0.84 (-0.99 to -0.69)
**Gabon**	Severe anemia	48 (11 to	77 (27 to	0.61 (-0.63 to	4.99 (2.23 to	1.93 (0.78 to	-2.43 (-2.85 to

		142)	151)	7.17)	8.87)	3.55)	-2.01)
**Gambia**	**Anemia**	146 (75 to 282)	1986 (1109 to 3120)	12.64 (5.92 to 25.6)	85.03 (74.52 to 91.25)	72.24 (65.24 to 78.43)	-0.65 (-0.78 to -0.51)
**Gambia**	Mild anemia	58 (30 to 113)	830 (457 to 1321)	13.33 (5.95 to 27.97)	33.74 (27.86 to 39.22)	30.16 (25.62 to 34.86)	-0.62 (-0.77 to -0.47)
**Gambia**	Moderat e anemia	80 (41 to 155)	1079 (609 to 1698)	12.56 (5.64 to 26.21)	46.5 (38.97 to 51.53)	39.27 (34.14 to 43.55)	-0.58 (-0.71 to -0.44)
**Gambia**	Severe anemia	8 (3 to 17)	77 (38 to 138)	8.45 (2.91 to 24.43)	4.79 (2.49 to 8.06)	2.81 (1.83 to 4.12)	-1.69 (-1.94 to -1.44)
**Georgia**	**Anemia**	6 (5 to 9)	39 (20 to 61)	5.53 (2.89 to 8.31)	43.68 (40.4 to 46.83)	41.11 (37.53 to 44.45)	-0.71 (-0.9 to -0.53)
**Georgia**	Mild anemia	3 (2 to 5)	24 (12 to 40)	6.07 (2.99 to 9.81)	24.85 (20.91 to 29.13)	25.3 (20.22 to 31.34)	-0.2 (-0.38 to -0.02)
**Georgia**	Moderat e anemia	2 (2 to 4)	13 (6 to 23)	4.49 (1.9 to 7.77)	17.67 (14.27 to 20.48)	13.94 (9.77 to 18.32)	-1.03 (-1.35 to -0.7)
**Georgia**	Severe anemia	0 (0 to 0)	2 (0 to 4)	9.74 (1.91 to 35.05)	1.17 (0.65 to 1.93)	1.87 (0.71 to 5.36)	-2.74 (-5.14 to -0.27)
**Germany**	**Anemia**	1364 (731 to 2093)	912 (544 to 1509)	-0.33 (-0.53 to 0.12)	32.86 (20.71 to 41.3)	36.52 (29.35 to 45.21)	1.09 (0.67 to 1.51)
**Germany**	Mild anemia	1083 (603 to 1677)	748 (443 to 1223)	-0.31 (-0.53 to 0.1)	26.09 (17.54 to 33.57)	29.98 (24.03 to 37.34)	1.19 (0.8 to 1.58)
**Germany**	Moderat e anemia	276 (118 to 494)	161 (88 to 271)	-0.42 (-0.68 to 0.24)	6.65 (3.23 to 10.6)	6.45 (4.51 to 8.83)	0.69 (-0.05 to 1.43)
**Germany**	Severe anemia	5 (1 to 13)	2 (1 to 6)	-0.52 (-0.86 to 0.73)	0.12 (0.04 to 0.28)	0.09 (0.03 to 0.22)	-0.35 (-1.17 to 0.47)
**Ghana**	**Anemia**	8996 (5778 to 13275)	22255 (15947 to 29563)	1.47 (0.63 to 2.87)	69.64 (55 to 83.5)	63.24 (50.36 to 69.44)	-0.48 (-0.74 to -0.22)
**Ghana**	Mild anemia	3700 (2412 to 5465)	11721 (7273 to 16280)	2.17 (0.84 to 4.03)	28.66 (22.76 to 35.12)	33.34 (22.78 to 41.55)	0.27 (-0.02 to 0.57)
**Ghana**	Moderat e anemia	4873 (2955 to 7345)	9844 (6383 to 14068)	1.02 (0.27 to 2.45)	37.71 (26.58 to 47.37)	27.93 (19.82 to 36.77)	-1.06 (-1.33 to -0.79)
**Ghana**	Severe	423	691	0.63	3.27	1.96	-1.77

	anemia	(153 to 948)	(266 to 1397)	(-0.52 to 4.6)	(1.27 to 6.65)	(0.79 to 3.84)	(-2.27 to -1.27)
**Greece**	**Anemia**	32 (16 to 58)	85 (57 to 121)	1.65 (0.46 to 3.96)	17.25 (9.12 to 29.69)	43.6 (31.03 to 55.95)	4.04 (3.59 to 4.5)
**Greece**	Mild anemia	28 (14 to 50)	71 (47 to 101)	1.57 (0.39 to 3.88)	14.9 (7.8 to 25.71)	36.51 (25.92 to 47.53)	3.93 (3.43 to 4.42)
**Greece**	Moderat e anemia	4 (2 to 9)	14 (8 to 21)	2.16 (0.57 to 5.64)	2.32 (1.12 to 4.45)	6.99 (4.37 to 9.99)	4.99 (3.51 to 6.49)
**Greece**	Severe anemia	0 (0 to 0)	0 (0 to 0)	1.82 (-0.46 to 13.23)	0.03 (0.01 to 0.1)	0.09 (0.02 to 0.23)	0.51 (-4.03 to 5.26)
**Greenland**	**Anemia**	3 (1 to 6)	3 (1 to 5)	-0.18 (-0.56 to 0.62)	30.19 (23.57 to 38.25)	48.19 (33.27 to 61.06)	1.28 (0.95 to 1.62)
**Greenland**	Mild anemia	2 (1 to 4)	2 (1 to 3)	-0.16 (-0.55 to 0.69)	20.71 (14.91 to 27.93)	33.9 (23.13 to 44.44)	1.39 (1.08 to 1.69)
**Greenland**	Moderat e anemia	1 (0 to 2)	1 (0 to 1)	-0.22 (-0.62 to 0.77)	9.08 (6.31 to 12.48)	13.77 (8.47 to 20.29)	1.05 (0.66 to 1.44)
**Greenland**	Severe anemia	0 (0 to 0)	0 (0 to 0)	-0.34 (-0.8 to 1.19)	0.4 (0.16 to 0.77)	0.51 (0.19 to 1.11)	1.03 (0.37 to 1.69)
**Grenada**	**Anemia**	5 (3 to 7)	10 (6 to 15)	1.08 (0.6 to 1.69)	51.06 (46.94 to 54.84)	63.87 (60.08 to 67.63)	0.61 (0.46 to 0.77)
**Grenada**	Mild anemia	3 (2 to 4)	6 (3 to 10)	1.09 (0.47 to 1.92)	30.09 (26.49 to 33.98)	37.82 (30.44 to 48.34)	0.65 (0.45 to 0.84)
**Grenada**	Moderat e anemia	2 (1 to 3)	4 (2 to 7)	1.09 (0.25 to 1.95)	19.91 (17.28 to 22.57)	25.06 (15.77 to 30.87)	0.58 (0.48 to 0.68)
**Grenada**	Severe anemia	0 (0 to 0)	0 (0 to 0)	0.54 (-0.42 to 2.49)	1.06 (0.64 to 1.6)	0.99 (0.34 to 2.09)	0.14 (-0.48 to 0.77)
**Guam**	**Anemia**	1 (1 to 3)	6 (2 to 11)	3.34 (0.99 to 9.44)	22.77 (17.06 to 29.97)	33.44 (27.42 to 40.81)	1.28 (0.51 to 2.06)
**Guam**	Mild anemia	1 (0 to 2)	4 (2 to 8)	3.24 (0.96 to 9.26)	16.06 (11.71 to 21.19)	22.9 (18.11 to 28.13)	1.19 (0.49 to 1.9)
**Guam**	Moderat e anemia	0 (0 to 1)	2 (1 to 4)	3.62 (0.91 to 10.09)	6.42 (4.4 to 9.27)	10.16 (7.31 to 13.96)	1.5 (0.59 to 2.43)

**Guam**	Severe anemia	0 (0 to 0)	0 (0 to 0)	2.83 (0.3 to 11.53)	0.3 (0.15 to 0.51)	0.39 (0.19 to 0.73)	0.98 (0.23 to 1.73)
**Guatemala**	**Anemia**	226 (160 to 317)	1888 (911 to 3548)	7.35 (3.62 to 13.23)	41.89 (38.14 to 45.68)	44.22 (36.62 to 49.48)	0.81 (0.56 to 1.06)
**Guatemala**	Mild anemia	123 (87 to 176)	1278 (597 to 2454)	9.37 (4.64 to 16.87)	22.86 (19.68 to 26.25)	29.95 (24.88 to 34.24)	1.57 (1.31 to 1.82)
**Guatemala**	Moderat e anemia	95 (66 to 132)	582 (261 to 1140)	5.13 (2.17 to 9.98)	17.61 (15.15 to 20.01)	13.63 (9.84 to 16.64)	-0.28 (-0.53 to -0.04)
**Guatemala**	Severe anemia	8 (4 to 13)	27 (10 to 62)	2.57 (0.37 to 7.49)	1.42 (0.9 to 2.21)	0.64 (0.35 to 1.05)	-2.23 (-2.5 to -1.97)
**Guinea**	**Anemia**	975 (574 to 1606)	9548 (5614 to 15132)	8.79 (4.34 to 16.68)	62.72 (53.72 to 72.21)	63.12 (51.07 to 73.56)	-0.07 (-0.14 to -0.01)
**Guinea**	Mild anemia	365 (215 to 600)	4288 (2427 to 6819)	10.76 (5.37 to 20.85)	23.44 (20 to 27.25)	28.29 (23.64 to 33.7)	0.6 (0.56 to 0.64)
**Guinea**	Moderat e anemia	549 (319 to 908)	4931 (2657 to 7898)	7.99 (3.49 to 16.62)	35.31 (28.36 to 42.2)	32.64 (22.1 to 40.24)	-0.38 (-0.46 to -0.3)
**Guinea**	Severe anemia	62 (28 to 124)	330 (140 to 650)	4.34 (0.96 to 13.59)	3.97 (2.14 to 6.51)	2.19 (1.1 to 3.86)	-2.37 (-2.58 to -2.15)
**Guinea-Bissau**	**Anemia**	321 (194 to 516)	2394 (1197 to 4056)	6.45 (3.16 to 12.6)	75.89 (59.97 to 88.75)	63.26 (55.49 to 70.57)	-0.49 (-0.67 to -0.32)
**Guinea-Bissau**	Mild anemia	135 (81 to 215)	1059 (528 to 1806)	6.83 (3.09 to 13.13)	31.95 (25.82 to 39.23)	27.9 (22.79 to 33.44)	-0.3 (-0.47 to -0.14)
**Guinea-Bissau**	Moderat e anemia	170 (93 to 278)	1240 (635 to 2027)	6.3 (2.96 to 12.8)	40.16 (27.74 to 50.3)	32.85 (27.01 to 37.52)	-0.57 (-0.74 to -0.4)
**Guinea-Bissau**	Severe anemia	16 (6 to 35)	95 (39 to 183)	4.9 (1.41 to 16.38)	3.79 (1.46 to 7.07)	2.51 (1.35 to 4.03)	-1.34 (-1.65 to -1.04)
**Guyana**	**Anemia**	215 (123 to 348)	541 (315 to 751)	1.52 (0.82 to 2.31)	54.77 (51.52 to 57.73)	67.31 (62.36 to 71.14)	0.54 (0.37 to 0.7)
**Guyana**	Mild anemia	103 (58 to 167)	299 (166 to 429)	1.92 (1.11 to 3)	26.17 (23.27 to 29.13)	37.26 (31.94 to 43.18)	1.11 (0.92 to 1.3)
**Guyana**	Moderat e anemia	104 (58 to	230 (131 to	1.22 (0.53 to	26.48 (23.93 to	28.68 (24.21 to	0.03 (-0.13 to

		175)	327)	2.06)	28.88)	33.15)	0.2)
**Guyana**	Severe anemia	8 (4 to 16)	11 (5 to 20)	0.33 (-0.35 to 1.78)	2.12 (1.44 to 3.06)	1.37 (0.79 to 2.28)	-1.74 (-1.85 to -1.64)
**Haiti**	**Anemia**	12180 (5386 to 20991)	11159 (7438 to 15947)	-0.08 (-0.47 to 0.95)	86.32 (76.92 to 92.18)	62.73 (56.68 to 69.75)	-1.35 (-1.47 to -1.24)
**Haiti**	Mild anemia	4436 (1968 to 7848)	4654 (3233 to 6675)	0.05 (-0.42 to 1.31)	31.41 (22.86 to 37.77)	26.23 (22.84 to 31.14)	-0.9 (-1.03 to -0.78)
**Haiti**	Moderate anemia	6746 (2941 to 11644)	6043 (3819 to 9130)	-0.1 (-0.49 to 0.93)	47.79 (41.35 to 54.27)	33.91 (29.11 to 39.36)	-1.38 (-1.49 to -1.27)
**Haiti**	Severe anemia	998 (373 to 1935)	461 (247 to 789)	-0.54 (-0.8 to 0.28)	7.12 (4.24 to 11.46)	2.59 (1.6 to 3.95)	-3.95 (-4.18 to -3.72)
**Honduras**	**Anemia**	210 (158 to 282)	513 (352 to 696)	1.44 (0.66 to 2.44)	44.49 (40.92 to 48.43)	48.92 (41.48 to 53.96)	0.17 (0.1 to 0.24)
**Honduras**	Mild anemia	133 (97 to 181)	361 (249 to 492)	1.72 (0.83 to 2.9)	28.11 (24.65 to 31.5)	34.47 (29.76 to 39.07)	0.57 (0.5 to 0.64)
**Honduras**	Moderate anemia	74 (54 to 100)	146 (92 to 211)	0.98 (0.22 to 2.01)	15.56 (13.31 to 17.68)	13.89 (10.36 to 17.09)	-0.6 (-0.7 to -0.5)
**Honduras**	Severe anemia	4 (2 to 6)	6 (3 to 10)	0.51 (-0.33 to 2.07)	0.82 (0.57 to 1.15)	0.56 (0.31 to 0.9)	-1.51 (-1.65 to -1.37)
**Hungary**	**Anemia**	24 (15 to 35)	20 (14 to 28)	-0.18 (-0.38 to 0.14)	30.76 (22.56 to 36.33)	47.94 (44.33 to 51.41)	1.33 (1.17 to 1.49)
**Hungary**	Mild anemia	17 (11 to 25)	15 (10 to 21)	-0.12 (-0.35 to 0.21)	22.05 (16.86 to 26.82)	36.56 (32.47 to 40.22)	1.59 (1.35 to 1.82)
**Hungary**	Moderate anemia	6 (4 to 10)	5 (3 to 7)	-0.3 (-0.52 to 0.15)	8.35 (5.34 to 10.97)	11.05 (8.88 to 13.82)	0.6 (0.27 to 0.92)
**Hungary**	Severe anemia	0 (0 to 1)	0 (0 to 0)	-0.52 (-0.79 to 0.16)	0.36 (0.16 to 0.63)	0.32 (0.16 to 0.59)	-0.32 (-3.9 to 3.39)
**Iceland**	**Anemia**	2 (1 to 3)	1 (0 to 1)	-0.53 (-0.88 to 0.45)	19.91 (8.29 to 35.73)	13.73 (3.76 to 25.27)	0 (-0.59 to 0.59)
**Iceland**	Mild anemia	1 (0 to 2)	1 (0 to 1)	-0.55 (-0.91 to 0.51)	16.75 (7.19 to 29.86)	10.91 (2.73 to 21.7)	-0.05 (-0.68 to 0.59)
**Iceland**	Moderat	0	0	-0.42	3.12	2.66	0.15

	e anemia	(0 to 1)	(0 to 1)	(-0.97 to 2.78)	(1.05 to 6.87)	(0.19 to 14.34)	(-0.26 to 0.56)
**Iceland**	Severe anemia	0 (0 to 0)	0 (0 to 0)	1.96 (-0.96 to 36.08)	0.04 (0 to 0.13)	0.16 (0 to 1.16)	2.41 (1.13 to 3.71)
**India**	**Anemia**	4570 (2254 to 10019)	89617 (70045 to 111889)	18.61 (7.79 to 41.37)	48.29 (44.21 to 51.75)	55.32 (52 to 58.51)	0.39 (0.25 to 0.52)
**India**	Mild anemia	1932 (957 to 4162)	35876 (28114 to 45254)	17.57 (7.35 to 38.6)	20.45 (18.76 to 22.17)	22.14 (20.74 to 23.56)	0.14 (-0.04 to 0.32)
**India**	Moderate anemia	2310 (1113 to 5040)	48805 (37988 to 61687)	20.13 (8.41 to 44.85)	24.36 (21.82 to 27.2)	30.13 (27.98 to 32.12)	0.69 (0.58 to 0.8)
**India**	Severe anemia	328 (159 to 735)	4935 (3746 to 6372)	14.06 (5.7 to 32.81)	3.48 (2.75 to 4.16)	3.05 (2.67 to 3.47)	-0.48 (-0.62 to -0.33)
**Indonesia**	**Anemia**	127 (0 to 226)	31492 (25940 to 38525)	246.57 (0 to 256.06)	36.57 (0 to 51.19)	42.45 (39.76 to 45.1)	-0.58 (-0.91 to -0.24)
**Indonesia**	Mild anemia	73 (0 to 131)	20632 (16291 to 26108)	282.8 (0 to 310.41)	20.91 (0 to 30.43)	27.81 (24.18 to 31.42)	-0.1 (-0.41 to 0.22)
**Indonesia**	Moderate anemia	51 (0 to 94)	10421 (7483 to 13892)	201.95 (0 to 229.4)	14.75 (0 to 21.72)	14.05 (10.79 to 17.29)	-1.18 (-1.55 to -0.82)
**Indonesia**	Severe anemia	3 (0 to 7)	439 (264 to 683)	138.03 (0 to 227.93)	0.91 (0 to 1.85)	0.59 (0.37 to 0.89)	-3.18 (-4.2 to -2.14)
**Iran (Islamic Republic of)**	**Anemia**	64 (46 to 92)	692 (371 to 1188)	9.77 (4.76 to 17.95)	25.13 (20.09 to 30.22)	29.74 (20.9 to 41.64)	1.01 (0.76 to 1.26)
**Iran (Islamic Republic of)**	Mild anemia	41 (29 to 59)	516 (274 to 856)	11.66 (5.67 to 21.31)	15.93 (12.9 to 19.49)	22.17 (15.82 to 30.3)	1.64 (1.4 to 1.87)
**Iran (Islamic Republic of)**	Moderate anemia	22 (15 to 32)	169 (87 to 307)	6.63 (2.77 to 13.49)	8.68 (6.33 to 11.17)	7.29 (4.55 to 11.06)	-0.03 (-0.43 to 0.37)
**Iran (Islamic Republic of)**	Severe anemia	1 (1 to 3)	7 (2 to 18)	4.13 (0.55 to 14.93)	0.52 (0.25 to 0.93)	0.29 (0.1 to 0.73)	-3.82 (-5.15 to -2.48)
**Iraq**	**Anemia**	14 (6 to 32)	386 (150 to 768)	26.92 (10.89 to 65.75)	63.12 (47.12 to 76.09)	58.4 (42.6 to 71.49)	-0.23 (-0.3 to -0.15)
**Iraq**	Mild anemia	8 (3 to 18)	238 (93 to 478)	29.11 (12.34 to 68.66)	35.95 (28.25 to 43.37)	35.94 (25.73 to 48.73)	0.28 (0.16 to 0.41)

**Iraq**	Moderate anemia	6 (2 to 13)	129 (43 to 265)	21.76 (7.85 to 56.2)	25.97 (16.95 to 34.07)	19.48 (10 to 26.24)	-1.07 (-1.19 to -0.95)
**Iraq**	Severe anemia	0 (0 to 1)	19 (1 to 120)	72.76 (3.8 to 654.48)	1.2 (0.55 to 2.32)	2.98 (0.24 to 18.21)	-0.31 (-1.45 to 0.85)
**Ireland**	**Anemia**	17 (9 to 26)	40 (21 to 63)	1.36 (0.41 to 3.14)	36.11 (24.63 to 42.84)	39.21 (29.84 to 47.93)	0.24 (-0.19 to 0.66)
**Ireland**	Mild anemia	14 (7 to 22)	34 (18 to 54)	1.48 (0.48 to 3.29)	29.21 (20.86 to 35.87)	33.28 (24.86 to 41.6)	0.4 (0.05 to 0.75)
**Ireland**	Moderate anemia	3 (1 to 6)	6 (3 to 9)	0.87 (-0.02 to 3.19)	6.81 (3.53 to 10.32)	5.86 (3.97 to 7.92)	-0.38 (-1.22 to 0.46)
**Ireland**	Severe anemia	0 (0 to 0)	0 (0 to 0)	0.38 (-0.69 to 5.08)	0.09 (0.02 to 0.24)	0.06 (0.01 to 0.16)	-5.86 (-7.49 to -4.21)
**Israel**	**Anemia**	37 (15 to 71)	151 (62 to 255)	3.04 (0.74 to 7.28)	28.51 (17.23 to 37.64)	25.41 (11.33 to 37.55)	-0.41 (-0.7 to -0.12)
**Israel**	Mild anemia	31 (13 to 59)	126 (54 to 213)	3.12 (0.81 to 7.23)	23.42 (14.63 to 31.14)	21.3 (9.59 to 31.01)	-0.33 (-0.61 to -0.04)
**Israel**	Moderate anemia	7 (2 to 13)	24 (8 to 47)	2.67 (0.22 to 8.67)	5.01 (2.24 to 8.05)	4.06 (1.44 to 7.32)	-0.79 (-1.11 to -0.48)
**Israel**	Severe anemia	0 (0 to 0)	0 (0 to 1)	2.03 (-0.57 to 15.82)	0.08 (0.02 to 0.2)	0.06 (0.01 to 0.17)	-1.5 (-1.76 to -1.24)
**Italy**	**Anemia**	1739 (752 to 3031)	769 (408 to 1152)	-0.56 (-0.8 to -0.08)	23.02 (11.21 to 35.58)	35.12 (17.81 to 46.76)	1.05 (0.64 to 1.46)
**Italy**	Mild anemia	1445 (627 to 2506)	638 (346 to 963)	-0.56 (-0.79 to -0.08)	19.12 (9.44 to 29.25)	29.14 (15.06 to 39.19)	1.06 (0.67 to 1.45)
**Italy**	Moderate anemia	289 (101 to 575)	129 (59 to 215)	-0.55 (-0.83 to 0.13)	3.83 (1.44 to 7.12)	5.9 (2.66 to 9.18)	1.03 (0.54 to 1.52)
**Italy**	Severe anemia	5 (1 to 16)	2 (0 to 5)	-0.66 (-0.94 to 1.02)	0.07 (0.01 to 0.22)	0.08 (0.02 to 0.23)	-0.46 (-1.08 to 0.17)
**Jamaica**	**Anemia**	261 (125 to 509)	686 (349 to 1156)	1.63 (0.81 to 3.06)	47.11 (41.85 to 52.89)	64.79 (61.66 to 68.24)	1.04 (0.84 to 1.24)
**Jamaica**	Mild anemia	160 (77 to	423 (205 to	1.64 (0.68 to	28.89 (24.21 to	39.87 (32.17 to	1.05 (0.82 to

		322)	759)	3.33)	34.3)	49.95)	1.27)
**Jamaica**	Moderate anemia	97 (44 to 186)	254 (112 to 468)	1.63 (0.43 to 3.36)	17.46 (13.87 to 21.1)	23.99 (14.65 to 30.33)	1.05 (0.86 to 1.23)
**Jamaica**	Severe anemia	4 (1 to 10)	10 (3 to 24)	1.35 (-0.18 to 5.35)	0.76 (0.35 to 1.45)	0.93 (0.36 to 1.92)	0.63 (0.47 to 0.79)
**Japan**	**Anemia**	143 (68 to 244)	682 (443 to 1002)	3.77 (2.46 to 6.28)	34.35 (31.47 to 38.14)	39.23 (36.06 to 42.75)	0.53 (0.46 to 0.61)
**Japan**	Mild anemia	103 (48 to 176)	528 (339 to 779)	4.15 (2.63 to 6.95)	24.62 (21.46 to 28.51)	30.33 (26.8 to 34.24)	0.84 (0.76 to 0.92)
**Japan**	Moderate anemia	37 (17 to 66)	151 (93 to 236)	3.04 (1.57 to 6.01)	8.99 (7.29 to 10.71)	8.7 (6.65 to 11.17)	-0.14 (-0.23 to -0.04)
**Japan**	Severe anemia	3 (1 to 7)	4 (1 to 7)	0.19 (-0.58 to 4.31)	0.75 (0.2 to 1.51)	0.21 (0.09 to 0.4)	-5.12 (-5.55 to -4.69)
**Jordan**	**Anemia**	3 (2 to 5)	36 (24 to 49)	9.59 (6.65 to 13.98)	57.14 (50.73 to 62.56)	44.08 (39.77 to 48.63)	-1.33 (-1.73 to -0.94)
**Jordan**	Mild anemia	2 (1 to 3)	22 (15 to 32)	10.22 (6.15 to 23.65)	33.66 (17.81 to 43.13)	27.58 (23.18 to 32.87)	-0.78 (-1.15 to -0.4)
**Jordan**	Moderate anemia	1 (1 to 2)	13 (8 to 19)	10.84 (5.62 to 19.66)	18.28 (13.98 to 29.52)	15.81 (11.83 to 19.27)	-1.73 (-2.43 to -1.03)
**Jordan**	Severe anemia	0 (0 to 1)	1 (0 to 1)	0.88 (-0.67 to 7.89)	5.2 (1.16 to 24.22)	0.69 (0.45 to 0.94)	-5.97 (-7.75 to -4.15)
**Kazakhstan**	**Anemia**	109 (54 to 188)	266 (179 to 373)	1.45 (0.66 to 2.77)	36.21 (31.04 to 41.6)	55.6 (52.46 to 58.89)	1.77 (1.67 to 1.87)
**Kazakhstan**	Mild anemia	64 (30 to 115)	153 (98 to 221)	1.4 (0.54 to 3.06)	21.16 (16.28 to 25.68)	31.96 (26.71 to 37.81)	1.89 (1.66 to 2.12)
**Kazakhstan**	Moderate anemia	40 (21 to 70)	109 (68 to 160)	1.69 (0.7 to 3.34)	13.45 (10.61 to 16.43)	22.68 (18.22 to 26.98)	1.8 (1.67 to 1.93)
**Kazakhstan**	Severe anemia	5 (1 to 20)	5 (2 to 10)	-0.02 (-0.8 to 2.87)	1.6 (0.58 to 6.04)	0.96 (0.45 to 1.89)	-0.94 (-1.24 to -0.64)
**Kenya**	**Anemia**	47241 (40371 to 54424)	63295 (52011 to 75676)	0.34 (0.15 to 0.56)	35.97 (32.42 to 39.78)	29.96 (26.42 to 33.55)	0.05 (-0.2 to 0.3)
**Kenya**	Mild	23514	35393	0.51	17.9	16.75	0.36

	anemia	(19968 to 27229)	(28992 to 42314)	(0.28 to 0.78)	(16.1 to 19.86)	(14.77 to 18.81)	(0.15 to 0.57)
**Kenya**	Moderate anemia	21337 (18049 to 24778)	25893 (21034 to 31180)	0.21 (0.03 to 0.42)	16.25 (14.38 to 18.2)	12.26 (10.6 to 13.88)	-0.2 (-0.52 to 0.12)
**Kenya**	Severe anemia	2391 (1948 to 2933)	2008 (1485 to 2652)	-0.16 (-0.37 to 0.12)	1.82 (1.51 to 2.19)	0.95 (0.73 to 1.23)	-1.38 (-1.74 to -1.02)
**Kiribati**	**Anemia**	2 (2 to 3)	4 (2 to 6)	0.69 (0.12 to 1.42)	76.02 (73.33 to 78.52)	60.49 (56.58 to 64.44)	-0.43 (-0.57 to -0.3)
**Kiribati**	Mild anemia	1 (1 to 2)	2 (1 to 3)	0.58 (-0.05 to 1.59)	40.69 (28.84 to 50.08)	30.36 (25.55 to 35.96)	-0.46 (-0.69 to -0.24)
**Kiribati**	Moderate anemia	1 (1 to 1)	2 (1 to 3)	1 (0.18 to 2.15)	29.94 (23.29 to 40.87)	28.18 (23.57 to 32.05)	-0.19 (-0.32 to -0.07)
**Kiribati**	Severe anemia	0 (0 to 0)	0 (0 to 0)	-0.23 (-0.79 to 1.34)	5.39 (2.13 to 12.49)	1.95 (0.95 to 3.46)	-2.4 (-2.93 to -1.86)
**Kuwait**	**Anemia**	4 (3 to 5)	10 (7 to 16)	1.63 (1 to 2.5)	45.41 (42.07 to 49.4)	48.22 (44.13 to 52.92)	0.17 (-0.1 to 0.44)
**Kuwait**	Mild anemia	2 (1 to 3)	7 (4 to 10)	2.28 (1.36 to 3.66)	23.08 (20.33 to 25.99)	30.56 (25.81 to 36.02)	0.56 (0.24 to 0.88)
**Kuwait**	Moderate anemia	2 (1 to 2)	4 (2 to 6)	1.03 (0.37 to 1.93)	20.75 (18.69 to 23.03)	16.96 (12.92 to 21.22)	-1.31 (-1.74 to -0.88)
**Kuwait**	Severe anemia	0 (0 to 0)	0 (0 to 0)	0.09 (-0.55 to 1.3)	1.58 (1.1 to 2.19)	0.7 (0.35 to 1.21)	2.66 (-0.79 to 6.23)
**Kyrgyzstan**	**Anemia**	16 (10 to 29)	324 (162 to 525)	19.42 (10.03 to 35.68)	35.79 (30.03 to 40.79)	60.86 (56.42 to 65.36)	2.43 (2.24 to 2.62)
**Kyrgyzstan**	Mild anemia	9 (5 to 17)	175 (83 to 290)	17.7 (8.72 to 34.61)	21.03 (14.75 to 25.63)	32.84 (28.42 to 37.6)	2.28 (2.04 to 2.52)
**Kyrgyzstan**	Moderate anemia	6 (3 to 11)	142 (71 to 231)	23.74 (12.34 to 43.89)	12.98 (9.79 to 16.46)	26.65 (22.79 to 30.66)	2.8 (2.55 to 3.06)
**Kyrgyzstan**	Severe anemia	1 (0 to 4)	7 (3 to 15)	8.36 (0.68 to 32.78)	1.78 (0.64 to 8.94)	1.38 (0.75 to 2.37)	0.24 (-0.11 to 0.59)
**Lao People's Democratic Republic**	**Anemia**	0 (0 to 2)	450 (156 to 1888)	1161.69 (309.1 to 25479.6)	47.5 (22.64 to 81.8)	35.72 (23.27 to 53.85)	-0.53 (-0.78 to -0.28)

**Lao People's Democratic Republic**	Mild anemia	0 (0 to 1)	248 (84 to 1060)	1275.86 (320.2 to 27372.62)	23.2 (13.35 to 37.69)	19.51 (13.58 to 27.99)	-0.18 (-0.38 to 0.02)
**Lao People's Democratic Republic**	Moderate anemia	0 (0 to 1)	193 (66 to 797)	1090.2 (285.72 to 24276.95)	22.23 (8.95 to 41.81)	15.44 (8.61 to 25.54)	-0.76 (-1.07 to -0.46)
**Lao People's Democratic Republic**	Severe anemia	0 (0 to 0)	9 (3 to 40)	579.85 (144.27 to 14072.54)	2.06 (0.77 to 4.25)	0.77 (0.42 to 1.33)	-3.01 (-3.48 to -2.55)
**Latvia**	**Anemia**	12 (6 to 22)	48 (23 to 78)	2.94 (1.26 to 5.76)	30.81 (18.77 to 38.62)	48.59 (28.31 to 54.97)	1.49 (1.29 to 1.69)
**Latvia**	Mild anemia	8 (4 to 14)	36 (17 to 60)	3.42 (1.67 to 6.76)	20.36 (12.46 to 26.37)	36.1 (22.08 to 43.36)	1.85 (1.62 to 2.08)
**Latvia**	Moderate anemia	3 (1 to 7)	12 (5 to 23)	2.62 (0.74 to 7.98)	8.3 (3.58 to 13.8)	12.15 (6.18 to 18.59)	0.98 (0.73 to 1.23)
**Latvia**	Severe anemia	1 (0 to 2)	0 (0 to 1)	-0.59 (-0.9 to 3.83)	2.15 (0.18 to 4.14)	0.35 (0.12 to 0.76)	-3.34 (-5.09 to -1.56)
**Lebanon**	**Anemia**	37 (5 to 143)	31 (1 to 169)	-0.15 (-0.9 to 2.61)	38.94 (25.05 to 65.36)	17.04 (6.37 to 35.95)	-2.99 (-3.15 to -2.84)
**Lebanon**	Mild anemia	24 (3 to 93)	25 (1 to 133)	0.06 (-0.88 to 3.67)	25.35 (16.51 to 40.27)	13.71 (5.2 to 28.35)	-2.22 (-2.38 to -2.05)
**Lebanon**	Moderate anemia	13 (2 to 51)	6 (0 to 34)	-0.52 (-0.95 to 1.26)	13.1 (7.31 to 23.55)	3.25 (1.03 to 7.68)	-5.09 (-5.32 to -4.87)
**Lebanon**	Severe anemia	0 (0 to 2)	0 (0 to 1)	-0.69 (-0.98 to 0.78)	0.49 (0.25 to 0.91)	0.08 (0.02 to 0.24)	-6.43 (-6.55 to -6.32)
**Lesotho**	**Anemia**	4272 (2967 to 5888)	14548 (9834 to 19211)	2.41 (1.05 to 4.39)	82.9 (74.02 to 86.97)	39.99 (23.67 to 56.67)	-1.91 (-2.72 to -1.1)
**Lesotho**	Mild anemia	2053 (1366 to 2981)	9398 (7189 to 11536)	3.58 (2 to 6.05)	39.82 (33.7 to 47.08)	25.72 (18.19 to 32.4)	-1.09 (-1.61 to -0.57)
**Lesotho**	Moderate anemia	2037 (1386 to 2882)	4810 (1757 to 7889)	1.36 (-0.17 to 3.53)	39.55 (31.63 to 44.58)	13.33 (4.3 to 23.19)	-2.94 (-4.11 to -1.74)
**Lesotho**	Severe anemia	182 (92 to 324)	339 (113 to 597)	0.86 (-0.44 to 3.59)	3.54 (2 to 5.67)	0.94 (0.26 to 1.76)	-3.7 (-5.16 to -2.21)
**Liberia**	**Anemia**	400 (86 to	1930 (1050 to	3.83 (0.5 to	70.11 (57.75 to	45.17 (27.02 to	-1.07 (-1.29 to

		1083)	3189)	23.19)	81.17)	62.19)	-0.86)
**Liberia**	Mild anemia	137 (28 to 361)	975 (557 to 1567)	6.11 (1.34 to 33.01)	24.06 (19.66 to 29.24)	22.76 (15.79 to 29.69)	0.21 (0.05 to 0.37)
**Liberia**	Moderate anemia	235 (51 to 655)	903 (398 to 1659)	2.84 (0 to 20.38)	41.25 (30.31 to 51.02)	21.19 (10.16 to 34.88)	-1.75 (-2.05 to -1.46)
**Liberia**	Severe anemia	27 (5 to 83)	52 (18 to 121)	0.91 (-0.58 to 10.82)	4.8 (2.01 to 8.47)	1.22 (0.43 to 2.68)	-4.11 (-4.57 to -3.65)
**Libya**	**Anemia**	22 (10 to 63)	175 (28 to 820)	6.87 (0.45 to 27.96)	55.8 (43.42 to 71.8)	60.94 (37.15 to 79.12)	-0.04 (-0.27 to 0.2)
**Libya**	Mild anemia	13 (6 to 36)	115 (18 to 556)	8.02 (0.63 to 31.2)	32.26 (23.47 to 41.94)	39.89 (25.27 to 54.2)	0.55 (0.29 to 0.81)
**Libya**	Moderate anemia	9 (4 to 26)	57 (9 to 270)	5.44 (0.11 to 22.82)	22.32 (14.3 to 31.64)	20.32 (10.89 to 28.89)	-0.84 (-1.96 to 0.29)
**Libya**	Severe anemia	0 (0 to 1)	2 (0 to 10)	3.15 (-0.48 to 17.06)	1.22 (0.54 to 2.29)	0.72 (0.29 to 1.4)	-2.73 (-4.2 to -1.23)
**Lithuania**	**Anemia**	14 (0 to 30)	13 (8 to 17)	-0.11 (-0.63 to 1.55)	59.69 (0 to 74.47)	28.97 (23.23 to 35.47)	-3.51 (-3.83 to -3.19)
**Lithuania**	Mild anemia	10 (0 to 21)	10 (6 to 13)	-0.03 (-0.59 to 1.63)	41.78 (0 to 53.09)	22.23 (17.07 to 28.09)	-3.32 (-3.69 to -2.95)
**Lithuania**	Moderate anemia	4 (0 to 9)	3 (2 to 4)	-0.31 (-0.73 to 1.26)	17.27 (0 to 26.43)	6.54 (4.55 to 9.41)	-4.23 (-4.55 to -3.91)
**Lithuania**	Severe anemia	0 (0 to 0)	0 (0 to 0)	-0.42 (-0.83 to 1.71)	0.65 (0 to 1.51)	0.2 (0.09 to 0.4)	-1.56 (-3.55 to 0.48)
**Luxembourg**	**Anemia**	3 (1 to 6)	7 (4 to 11)	1.28 (0.3 to 4.48)	29.83 (13.93 to 41.1)	42.17 (28.93 to 50.2)	1.38 (1.23 to 1.54)
**Luxembourg**	Mild anemia	3 (1 to 5)	6 (3 to 10)	1.31 (0.25 to 4.53)	24.5 (12.02 to 34.65)	35.12 (24.35 to 42.14)	1.41 (1.26 to 1.56)
**Luxembourg**	Moderate anemia	1 (0 to 1)	1 (1 to 2)	1.14 (0.07 to 5.45)	5.25 (1.96 to 9.36)	6.97 (4.21 to 9.59)	1.27 (1.06 to 1.48)
**Luxembourg**	Severe anemia	0 (0 to 0)	0 (0 to 0)	0.73 (-0.69 to 9.16)	0.08 (0.01 to 0.22)	0.08 (0.02 to 0.22)	0.76 (0.4 to 1.12)
**Madagascar**	**Anemia**	13	3852	290.62	81.51	74.65	-0.18

		(4 to 25)	(1765 to 7098)	(116.94 to 964.57)	(68.81 to 91.94)	(60.23 to 83.85)	(-0.35 to -0.02)
**Madagascar**	Mild anemia	6 (2 to 12)	1980 (910 to 3666)	336.7 (139.15 to 1086.3)	36.19 (30.84 to 41.52)	38.34 (31.88 to 44.96)	0.42 (0.24 to 0.6)
**Madagascar**	Moderate anemia	7 (2 to 13)	1765 (783 to 3236)	262.03 (104.34 to 877.22)	41.39 (33.19 to 48.69)	34.22 (25.14 to 40.7)	-0.6 (-0.74 to -0.46)
**Madagascar**	Severe anemia	1 (0 to 1)	108 (38 to 227)	167.78 (46.29 to 695.82)	3.93 (2.34 to 6.09)	2.09 (1.1 to 3.46)	-2.42 (-2.71 to -2.13)
**Malawi**	**Anemia**	62007 (44091 to 82928)	75566 (60315 to 91184)	0.22 (-0.12 to 0.8)	70.53 (59.49 to 80.58)	60.08 (51.69 to 65.06)	-0.23 (-0.4 to -0.05)
**Malawi**	Mild anemia	21925 (14638 to 30288)	35082 (27040 to 44452)	0.6 (0.11 to 1.56)	24.88 (20.79 to 29.6)	27.88 (23.67 to 32.45)	0.84 (0.65 to 1.03)
**Malawi**	Moderate anemia	35670 (25482 to 48583)	37847 (28725 to 46910)	0.06 (-0.27 to 0.62)	40.6 (32.29 to 47.77)	30.1 (23.86 to 34.91)	-0.78 (-0.97 to -0.59)
**Malawi**	Severe anemia	4413 (2323 to 7558)	2637 (1607 to 4081)	-0.4 (-0.71 to 0.27)	5.04 (2.71 to 8.22)	2.1 (1.31 to 3.21)	-2.86 (-3 to -2.72)
**Malaysia**	**Anemia**	169 (123 to 233)	1719 (1357 to 2112)	9.17 (6.1 to 12.34)	26.87 (24.41 to 29.15)	38.45 (35.25 to 42.03)	2.14 (1.69 to 2.58)
**Malaysia**	Mild anemia	89 (63 to 123)	1030 (788 to 1320)	10.64 (6.94 to 15.07)	14.06 (12.67 to 15.39)	23.06 (19.42 to 27.09)	2.51 (2.08 to 2.94)
**Malaysia**	Moderate anemia	73 (52 to 99)	646 (455 to 824)	7.88 (4.66 to 11.31)	11.58 (10.28 to 12.86)	14.45 (11.21 to 17.01)	1.72 (1.24 to 2.21)
**Malaysia**	Severe anemia	8 (5 to 11)	42 (19 to 159)	4.5 (1.37 to 18.01)	1.23 (1 to 1.48)	0.95 (0.46 to 3.45)	0.71 (0.1 to 1.32)
**Maldives**	**Anemia**	1 (0 to 1)	2 (1 to 4)	2.73 (1.02 to 5.25)	78.07 (66.94 to 86.29)	55.75 (34.95 to 78.75)	-1.13 (-1.47 to -0.78)
**Maldives**	Mild anemia	0 (0 to 0)	2 (1 to 3)	5.18 (1.7 to 12.43)	34.13 (19.91 to 53.5)	40.35 (24.2 to 59.81)	0.17 (-0.44 to 0.79)
**Maldives**	Moderate anemia	0 (0 to 0)	1 (0 to 1)	0.87 (0.01 to 2.25)	41.93 (29.58 to 48.72)	15.02 (9.32 to 21.81)	-3.2 (-3.36 to -3.05)
**Maldives**	Severe anemia	0 (0 to 0)	0 (0 to 0)	-0.01 (-0.7 to 2.19)	2.01 (0.71 to 5.33)	0.38 (0.15 to 0.74)	-5.96 (-6.09 to -5.83)

**Mali**	**Anemia**	2612 (783 to 6678)	9342 (6056 to 14050)	2.58 (0.28 to 10.89)	80.8 (70.88 to 90.14)	66.94 (60.39 to 73.71)	-0.58 (-0.6 to -0.55)
**Mali**	Mild anemia	866 (266 to 2360)	3819 (2385 to 6014)	3.41 (0.53 to 13.41)	26.67 (22.04 to 32.12)	27.33 (22.07 to 33.27)	0.07 (-0.01 to 0.14)
**Mali**	Moderat e anemia	1559 (457 to 4007)	5060 (3263 to 7684)	2.25 (0.17 to 10.13)	48.27 (39.07 to 56.54)	36.29 (31.4 to 41.3)	-0.85 (-0.9 to -0.79)
**Mali**	Severe anemia	188 (49 to 461)	463 (240 to 838)	1.47 (-0.24 to 9.28)	5.86 (2.98 to 9.79)	3.33 (1.95 to 5.28)	-1.86 (-1.98 to -1.73)
**Malta**	**Anemia**	3 (1 to 4)	6 (4 to 9)	1.2 (0.57 to 3.2)	36.6 (19.99 to 46.93)	50.04 (42.05 to 56.67)	1.51 (1.24 to 1.78)
**Malta**	Mild anemia	2 (1 to 4)	5 (3 to 8)	1.23 (0.57 to 3.18)	29.88 (16.94 to 38.5)	41.57 (34.21 to 48.12)	1.57 (1.3 to 1.85)
**Malta**	Moderat e anemia	0 (0 to 1)	1 (1 to 2)	1.03 (0.14 to 3.86)	6.62 (2.78 to 10.64)	8.36 (5.66 to 11.24)	1.2 (0.94 to 1.45)
**Malta**	Severe anemia	0 (0 to 0)	0 (0 to 0)	0.76 (-0.61 to 7.3)	0.1 (0.02 to 0.25)	0.11 (0.02 to 0.27)	0.87 (0.59 to 1.15)
**Marshall Islands**	**Anemia**	1 (0 to 8)	8 (0 to 53)	6.34 (-0.12 to 58.89)	68.37 (42.19 to 88.96)	59.5 (33.39 to 86.53)	-0.53 (-0.65 to -0.4)
**Marshall Islands**	Mild anemia	1 (0 to 4)	5 (0 to 31)	6.96 (-0.07 to 63.77)	36.9 (23.87 to 48.69)	34.73 (20.26 to 51.34)	-0.21 (-0.38 to -0.04)
**Marshall Islands**	Moderat e anemia	0 (0 to 3)	3 (0 to 20)	5.7 (-0.2 to 54.82)	29.47 (16.93 to 39.92)	23.47 (11.5 to 35.95)	-0.87 (-0.96 to -0.79)
**Marshall Islands**	Severe anemia	0 (0 to 0)	0 (0 to 1)	4.35 (-0.44 to 46.67)	2 (0.92 to 3.88)	1.31 (0.53 to 2.5)	-1.66 (-1.76 to -1.56)
**Mauritania**	**Anemia**	49 (29 to 141)	41 (17 to 179)	-0.16 (-0.75 to 2.32)	44.71 (37.42 to 51.92)	45.27 (26.09 to 60.39)	0.48 (0.28 to 0.68)
**Mauritania**	Mild anemia	25 (14 to 72)	23 (9 to 97)	-0.1 (-0.7 to 2.49)	23.11 (18.94 to 27.73)	25 (15.85 to 33.99)	0.75 (0.57 to 0.93)
**Mauritania**	Moderat e anemia	22 (13 to 61)	17 (6 to 75)	-0.21 (-0.8 to 2.15)	20.04 (15.9 to 24.58)	19.25 (8.83 to 26.68)	0.26 (0.02 to 0.49)
**Mauritania**	Severe anemia	2 (1 to 5)	1 (0 to 4)	-0.46 (-0.89 to	1.56 (0.8 to	1.02 (0.37 to	-1 (-1.35 to

				1.19)	2.76)	1.97)	-0.65)
**Mauritius**	**Anemia**	4 (3 to 5)	66 (26 to 118)	14.75 (5.65 to 24.31)	68.04 (53.99 to 73.72)	37.36 (16.86 to 54.12)	-1.83 (-2.4 to -1.24)
**Mauritius**	Mild anemia	3 (2 to 4)	51 (20 to 94)	18.86 (6.24 to 37.43)	41.72 (26.65 to 58.05)	28.86 (12.73 to 42.19)	-1.08 (-1.62 to -0.53)
**Mauritius**	Moderate anemia	1 (1 to 2)	14 (6 to 29)	9.62 (3.58 to 20.15)	22.17 (10.38 to 31.92)	8.23 (3.67 to 15.26)	-3.3 (-3.83 to -2.77)
**Mauritius**	Severe anemia	0 (0 to 1)	0 (0 to 1)	0.89 (-0.79 to 20.85)	4.15 (0.41 to 18.16)	0.27 (0.09 to 0.67)	-7.25 (-8.93 to -5.55)
**Mexico**	**Anemia**	1527 (938 to 2317)	5607 (3137 to 8467)	2.67 (1.97 to 3.54)	18.58 (17.09 to 20.18)	21.17 (18.98 to 23.17)	0.35 (0.09 to 0.62)
**Mexico**	Mild anemia	1025 (628 to 1558)	3897 (2176 to 5925)	2.8 (2.03 to 3.72)	12.47 (11.47 to 13.6)	14.7 (13.2 to 16.12)	0.47 (0.19 to 0.76)
**Mexico**	Moderate anemia	469 (288 to 714)	1604 (891 to 2402)	2.42 (1.71 to 3.28)	5.71 (5.22 to 6.23)	6.06 (5.28 to 6.87)	0.12 (-0.14 to 0.38)
**Mexico**	Severe anemia	33 (20 to 51)	105 (63 to 155)	2.16 (1.37 to 3.33)	0.4 (0.35 to 0.46)	0.4 (0.31 to 0.54)	-0.37 (-0.56 to -0.18)
**Micronesia (Federated States of)**	**Anemia**	3 (0 to 17)	21 (1 to 114)	6.97 (0.04 to 54.52)	64.16 (39.38 to 84.46)	43.68 (24.38 to 71.95)	-1.24 (-1.61 to -0.88)
**Micronesia (Federated States of)**	Mild anemia	1 (0 to 10)	12 (0 to 65)	7.16 (0.07 to 55.86)	36.89 (23.84 to 50.35)	26.21 (14.54 to 43.86)	-0.99 (-1.31 to -0.67)
**Micronesia (Federated States of)**	Moderate anemia	1 (0 to 6)	8 (0 to 46)	6.93 (-0.02 to 53.06)	25.25 (12.87 to 35.85)	16.65 (8.76 to 27.96)	-1.12 (-1.63 to -0.6)
**Micronesia (Federated States of)**	Severe anemia	0 (0 to 0)	0 (0 to 2)	4.04 (-0.46 to 42.66)	2.02 (0.73 to 4.06)	0.82 (0.34 to 1.71)	-5.98 (-7.15 to -4.8)
**Monaco**	**Anemia**	0 (0 to 1)	0 (0 to 1)	0.23 (-0.63 to 5.74)	27.43 (10.75 to 54.35)	37.64 (13.21 to 61.22)	1.16 (0.91 to 1.41)
**Monaco**	Mild anemia	0 (0 to 1)	0 (0 to 1)	0.23 (-0.63 to 5.81)	23.04 (9.45 to 44.51)	31.45 (10.98 to 51.04)	1.15 (0.89 to 1.4)
**Monaco**	Moderate anemia	0 (0 to 0)	0 (0 to 0)	0.27 (-0.69 to 6.75)	4.33 (1.36 to 9.84)	6.12 (2.13 to 10.8)	1.24 (0.98 to 1.49)
**Monaco**	Severe	0	0	0.23	0.05	0.07	1.29

	anemia	(0 to 0)	(0 to 0)	(-0.84 to 13.25)	(0.01 to 0.17)	(0.01 to 0.21)	(1.04 to 1.54)
**Mongolia**	**Anemia**	0 (0 to 0)	7 (3 to 13)	272.62 (0 to 0)	0.3 (0 to 0)	33.52 (27.03 to 39.53)	3.02 (-0.5 to 6.66)
**Mongolia**	Mild anemia	0 (0 to 0)	4 (2 to 7)	269.34 (0 to 0)	0.16 (0 to 0)	18.24 (14.21 to 22.38)	3.18 (-0.25 to 6.72)
**Mongolia**	Moderate anemia	0 (0 to 0)	3 (1 to 6)	283.73 (0 to 0)	0.12 (0 to 0)	13.73 (9.69 to 17.55)	3.38 (-0.25 to 7.14)
**Mongolia**	Severe anemia	0 (0 to 0)	0 (0 to 2)	226.5 (0 to 0)	0.02 (0 to 0)	1.55 (0.76 to 7.03)	-0.01 (-3.78 to 3.92)
**Montenegro**	**Anemia**	1 (1 to 2)	5 (3 to 7)	3.36 (2.18 to 5.11)	44.29 (34.23 to 51.44)	44.81 (36.03 to 50.23)	-0.05 (-0.18 to 0.08)
**Montenegro**	Mild anemia	1 (1 to 1)	4 (2 to 5)	3.47 (2.28 to 5.09)	32.46 (25.66 to 37.87)	33.68 (27.8 to 38.63)	0.07 (-0.05 to 0.2)
**Montenegro**	Moderate anemia	0 (0 to 0)	1 (1 to 2)	3.09 (1.53 to 5.55)	11.39 (7.36 to 14.95)	10.81 (7.24 to 14.05)	-0.37 (-0.54 to -0.2)
**Montenegro**	Severe anemia	0 (0 to 0)	0 (0 to 0)	2.16 (0.43 to 6.68)	0.44 (0.2 to 0.81)	0.32 (0.14 to 0.59)	-1.47 (-1.68 to -1.25)
**Morocco**	**Anemia**	265 (98 to 793)	1180 (112 to 6520)	3.45 (-0.4 to 16.12)	58.62 (47.08 to 75.52)	49.86 (31.81 to 73.14)	-0.74 (-0.81 to -0.67)
**Morocco**	Mild anemia	146 (53 to 447)	786 (74 to 4453)	4.4 (-0.29 to 18.82)	32.28 (25.59 to 42.02)	33.38 (21.11 to 49.59)	-0.06 (-0.15 to 0.04)
**Morocco**	Moderate anemia	111 (40 to 329)	378 (33 to 2047)	2.41 (-0.57 to 12.16)	24.47 (17.52 to 32.35)	15.83 (9.14 to 25.32)	-1.76 (-1.97 to -1.56)
**Morocco**	Severe anemia	9 (2 to 29)	15 (1 to 83)	0.79 (-0.83 to 7.53)	1.87 (0.88 to 3.6)	0.64 (0.28 to 1.18)	-2.14 (-3.78 to -0.47)
**Mozambique**	**Anemia**	14851 (8854 to 24651)	226407 (177777 to 280963)	14.25 (7.96 to 25.94)	79.21 (66.74 to 86.34)	53.17 (43.35 to 61.94)	-1.35 (-1.54 to -1.16)
**Mozambique**	Mild anemia	6415 (3671 to 10703)	114973 (86901 to 148024)	16.92 (9.02 to 31.93)	34.18 (25.98 to 42.5)	26.93 (22.45 to 31.89)	-0.85 (-1.06 to -0.64)
**Mozambique**	Moderate anemia	7600 (4274 to	103959 (78394 to	12.68 (6.61 to	40.55 (30.43 to	24.47 (17.97 to	-1.67 (-1.85 to

		12590)	135518)	25.7)	49.79)	30.78)	-1.49)
**Mozambique**	Severe anemia	836 (343 to 1735)	7475 (4155 to 12323)	7.94 (2.79 to 24.91)	4.47 (2.04 to 7.95)	1.77 (0.94 to 3.01)	-3.07 (-3.3 to -2.84)
**Myanmar**	**Anemia**	956 (730 to 1249)	10458 (5931 to 14409)	9.94 (6.91 to 12.47)	45.87 (36.44 to 57.26)	51.84 (42.45 to 57.83)	0.08 (-0.27 to 0.44)
**Myanmar**	Mild anemia	481 (370 to 624)	5461 (3101 to 7629)	10.35 (6.54 to 13.7)	23.1 (18.55 to 28.92)	27.1 (22.15 to 31.21)	0.34 (-0.03 to 0.7)
**Myanmar**	Moderate anemia	440 (317 to 606)	4789 (2703 to 6615)	9.88 (6.8 to 12.78)	21.12 (15.59 to 27.72)	23.71 (18.64 to 27.69)	-0.08 (-0.44 to 0.28)
**Myanmar**	Severe anemia	34 (18 to 59)	208 (101 to 363)	5.09 (1.97 to 11.52)	1.64 (0.86 to 2.8)	1.03 (0.6 to 1.67)	-1.84 (-2.42 to -1.26)
**Namibia**	**Anemia**	1767 (986 to 2420)	6804 (4137 to 9238)	2.85 (1.09 to 5.73)	80.3 (44.22 to 87.19)	39.24 (23.48 to 52.59)	-2.2 (-2.47 to -1.93)
**Namibia**	Mild anemia	775 (517 to 1083)	4157 (2466 to 5584)	4.36 (2.02 to 7.83)	35.22 (25.46 to 43.45)	23.96 (14.98 to 31.21)	-1.22 (-1.4 to -1.03)
**Namibia**	Moderate anemia	907 (405 to 1260)	2511 (1200 to 3640)	1.77 (0.33 to 4.75)	41.21 (18.2 to 48.02)	14.49 (6.8 to 20.55)	-3.23 (-3.61 to -2.84)
**Namibia**	Severe anemia	85 (29 to 153)	137 (65 to 215)	0.61 (-0.37 to 3.48)	3.87 (1.37 to 6.92)	0.79 (0.36 to 1.23)	-4.92 (-5.45 to -4.39)
**Nauru**	**Anemia**	0 (0 to 0)	1 (0 to 5)	23.72 (2.99 to 69.76)	39.12 (24.73 to 52.13)	46.56 (25.95 to 73.91)	0.47 (0.24 to 0.71)
**Nauru**	Mild anemia	0 (0 to 0)	1 (0 to 3)	24.37 (3.27 to 73.17)	23.25 (15.68 to 31.83)	28.8 (16.94 to 44.85)	0.6 (0.47 to 0.73)
**Nauru**	Moderate anemia	0 (0 to 0)	0 (0 to 2)	23.06 (2.67 to 67.87)	14.98 (8.19 to 21.33)	17 (8.09 to 29.69)	0.33 (-0.03 to 0.7)
**Nauru**	Severe anemia	0 (0 to 0)	0 (0 to 0)	17.11 (1.24 to 67.67)	0.89 (0.39 to 1.66)	0.76 (0.28 to 1.64)	-0.6 (-1.18 to -0.01)
**Nepal**	**Anemia**	1 (0 to 5)	1367 (83 to 5701)	1714.06 (231.03 to 21141.02)	39.15 (22.98 to 66.28)	43.22 (26.31 to 66.17)	0.62 (0.51 to 0.73)
**Nepal**	Mild anemia	0 (0 to 2)	765 (47 to 3331)	2018.04 (266.95 to 25171.34)	19 (13.16 to 26.61)	24.42 (16.13 to 35.77)	1.15 (1.02 to 1.29)
**Nepal**	Moderat	0	575	1522.29	18.16	17.95	0.23

	e anemia	(0 to 3)	(33 to 2537)	(191.11 to 18097.5)	(7.94 to 35.53)	(8.96 to 29.72)	(0.12 to 0.34)
**Nepal**	Severe anemia	0 (0 to 0)	27 (1 to 131)	668.84 (80.6 to 8449.79)	1.99 (0.73 to 4.19)	0.84 (0.38 to 1.59)	-2.8 (-2.92 to -2.67)
**Netherlands**	**Anemia**	246 (125 to 381)	244 (127 to 358)	-0.01 (-0.52 to 0.91)	29.2 (15.15 to 36.23)	42.12 (20.39 to 51.28)	0.66 (0.27 to 1.05)
**Netherlands**	Mild anemia	185 (66 to 320)	203 (105 to 298)	0.09 (-0.53 to 1.63)	22.03 (10.39 to 32.3)	34.98 (17.51 to 43.14)	0.82 (0.35 to 1.29)
**Netherlands**	Moderat e anemia	59 (9 to 164)	41 (18 to 66)	-0.31 (-0.77 to 3.37)	7.01 (1.16 to 17.76)	7.05 (3.12 to 9.75)	0.03 (-0.17 to 0.22)
**Netherlands**	Severe anemia	1 (0 to 5)	0 (0 to 1)	-0.64 (-0.95 to 5.07)	0.16 (0.01 to 0.58)	0.08 (0.01 to 0.23)	-1.38 (-1.99 to -0.77)
**New Zealand**	**Anemia**	20 (12 to 30)	63 (34 to 103)	2.16 (1.04 to 3.51)	18.11 (13.82 to 22.75)	48.23 (37.3 to 56.92)	2.52 (1.96 to 3.08)
**New Zealand**	Mild anemia	16 (10 to 23)	50 (26 to 80)	2.18 (1.04 to 3.59)	14.19 (10.91 to 17.72)	38.07 (29.69 to 45.16)	2.49 (1.93 to 3.06)
**New Zealand**	Moderat e anemia	4 (2 to 7)	13 (6 to 23)	2.09 (0.71 to 4.27)	3.82 (2.61 to 5.23)	9.95 (6.67 to 13.83)	2.54 (1.96 to 3.12)
**New Zealand**	Severe anemia	0 (0 to 0)	0 (0 to 1)	1.59 (-0.24 to 6.41)	0.1 (0.05 to 0.18)	0.22 (0.07 to 0.5)	4.27 (2.81 to 5.74)
**Nicaragua**	**Anemia**	35 (23 to 52)	738 (389 to 1147)	20.26 (11.35 to 29.65)	41.55 (36.68 to 46.07)	34.57 (21.37 to 43.8)	-0.07 (-0.32 to 0.19)
**Nicaragua**	Mild anemia	22 (14 to 33)	554 (299 to 859)	24.24 (13.9 to 35.63)	26.28 (22.51 to 30.19)	25.96 (16.52 to 32.89)	0.61 (0.34 to 0.87)
**Nicaragua**	Moderat e anemia	12 (8 to 19)	178 (83 to 304)	13.72 (6.24 to 23.84)	14.51 (11.88 to 17.1)	8.35 (4.52 to 11.86)	-1.51 (-1.73 to -1.3)
**Nicaragua**	Severe anemia	1 (0 to 1)	5 (2 to 12)	7.63 (1.93 to 20.09)	0.76 (0.48 to 1.16)	0.26 (0.11 to 0.49)	-3.62 (-3.86 to -3.39)
**Niger**	**Anemia**	1204 (551 to 2349)	2407 (1534 to 3561)	1 (0.03 to 3.27)	67.08 (56.33 to 77.91)	49.03 (41.53 to 57.06)	-1.52 (-1.72 to -1.32)
**Niger**	Mild anemia	450 (212 to 855)	1016 (642 to 1536)	1.26 (0.13 to 3.8)	25.09 (21.11 to 30.47)	20.7 (17.56 to 24.76)	-1.13 (-1.37 to -0.9)

**Niger**	Moderate anemia	664 (302 to 1306)	1293 (796 to 1936)	0.95 (-0.05 to 3.3)	36.99 (29.77 to 44.18)	26.35 (20.76 to 31.66)	-1.56 (-1.74 to -1.39)
**Niger**	Severe anemia	90 (35 to 191)	98 (48 to 186)	0.08 (-0.57 to 2.23)	5 (2.75 to 7.86)	1.98 (1.19 to 3.18)	-3.88 (-4.19 to -3.57)
**Nigeria**	**Anemia**	16538 (11084 to 23273)	109070 (85306 to 135051)	5.6 (3.72 to 8.63)	41.17 (34.1 to 49.01)	62.73 (56.51 to 68.18)	1.78 (1.65 to 1.92)
**Nigeria**	Mild anemia	9134 (6309 to 12890)	51896 (39785 to 66669)	4.68 (3 to 7.28)	22.74 (19.34 to 26.82)	29.84 (25.32 to 34.47)	1.03 (0.98 to 1.08)
**Nigeria**	Moderate anemia	6957 (4287 to 10083)	53632 (39019 to 69649)	6.71 (3.96 to 11.16)	17.32 (12.51 to 22.29)	30.85 (24.74 to 36.17)	2.52 (2.3 to 2.74)
**Nigeria**	Severe anemia	446 (197 to 830)	3542 (1911 to 6070)	6.93 (2.75 to 18.05)	1.11 (0.53 to 2.01)	2.04 (1.14 to 3.38)	2.76 (2.38 to 3.14)
**Niue**	**Anemia**	0 (0 to 0)	0 (0 to 0)	13.29 (1.01 to 39.78)	37.47 (23.83 to 51.64)	45.59 (24.7 to 73.7)	0.42 (0.19 to 0.65)
**Niue**	Mild anemia	0 (0 to 0)	0 (0 to 0)	14.45 (1.27 to 50.38)	22.72 (13.98 to 33.97)	30.66 (16.7 to 48.46)	0.79 (0.56 to 1.02)
**Niue**	Moderate anemia	0 (0 to 0)	0 (0 to 0)	14.54 (1.09 to 47.14)	10.98 (5.54 to 17.34)	14.42 (6.52 to 25.12)	0.24 (-0.2 to 0.67)
**Niue**	Severe anemia	0 (0 to 0)	0 (0 to 0)	0.84 (-0.85 to 21.59)	3.76 (0.35 to 10.86)	0.51 (0.18 to 1.16)	-4.95 (-6.31 to -3.58)
**North Macedonia**	**Anemia**	1 (1 to 1)	5 (3 to 6)	4.88 (3.46 to 6.65)	47.27 (41.7 to 52.17)	53.45 (50.07 to 56.93)	0.59 (0.46 to 0.71)
**North Macedonia**	Mild anemia	1 (0 to 1)	3 (2 to 4)	5.03 (3.34 to 7.03)	30.95 (26.89 to 35.11)	35.91 (31.19 to 41.13)	0.67 (0.52 to 0.81)
**North Macedonia**	Moderate anemia	0 (0 to 0)	1 (1 to 2)	4.62 (2.55 to 7.23)	15.44 (12.16 to 18.05)	16.68 (12.42 to 20.36)	0.47 (0.38 to 0.57)
**North Macedonia**	Severe anemia	0 (0 to 0)	0 (0 to 0)	4.16 (0.75 to 8.38)	0.88 (0.55 to 1.28)	0.86 (0.36 to 1.18)	-0.49 (-0.67 to -0.31)
**Northern Mariana Islands**	**Anemia**	0 (0 to 1)	2 (1 to 4)	3.09 (0.5 to 8.81)	63.92 (56.59 to 69.59)	56.77 (42.2 to 65.98)	-0.54 (-1.03 to -0.04)
**Northern Mariana Islands**	Mild anemia	0 (0 to 1)	1 (0 to 3)	3.47 (0.6 to	39.96 (33.02 to	38.88 (28.63 to	-0.18 (-0.65 to

				9.66)	46.68)	47.37)	0.29)
**Northern Mariana Islands**	Moderate anemia	0 (0 to 0)	1 (0 to 1)	2.49 (0.15 to 7.97)	23.02 (17.35 to 28.95)	17.34 (11.64 to 23.44)	-1.05 (-1.65 to -0.45)
**Northern Mariana Islands**	Severe anemia	0 (0 to 0)	0 (0 to 0)	1.71 (-0.33 to 8.28)	0.95 (0.42 to 1.85)	0.55 (0.24 to 1.06)	-4.15 (-4.98 to -3.32)
**Norway**	**Anemia**	16 (8 to 28)	108 (58 to 169)	5.56 (2.42 to 12.06)	24.44 (16.13 to 34.46)	31.38 (17.73 to 44.62)	0.66 (0.32 to 1)
**Norway**	Mild anemia	14 (7 to 24)	88 (48 to 136)	5.38 (2.36 to 11.44)	20.56 (13.51 to 28.8)	25.61 (14.63 to 36.3)	0.52 (0.19 to 0.84)
**Norway**	Moderate anemia	3 (1 to 5)	19 (9 to 33)	6.62 (2.34 to 15.41)	3.8 (2.29 to 5.69)	5.68 (2.88 to 9.1)	1.4 (0.93 to 1.87)
**Norway**	Severe anemia	0 (0 to 0)	0 (0 to 1)	4.01 (0.01 to 21.7)	0.09 (0.03 to 0.18)	0.09 (0.02 to 0.21)	0.52 (-0.14 to 1.18)
**Oman**	**Anemia**	3 (2 to 6)	71 (40 to 98)	19.87 (11.88 to 29.77)	46.22 (38.25 to 52.99)	53.97 (33.72 to 60.03)	1.09 (0.73 to 1.46)
**Oman**	Mild anemia	2 (1 to 3)	56 (32 to 80)	25.83 (14.99 to 40.2)	28.32 (22.92 to 33.98)	42.56 (27.58 to 49.93)	1.58 (1.2 to 1.95)
**Oman**	Moderate anemia	1 (1 to 2)	15 (7 to 30)	10.52 (4.51 to 22.69)	17.31 (12.15 to 21.82)	11.14 (5.7 to 20.74)	-0.02 (-0.59 to 0.56)
**Oman**	Severe anemia	0 (0 to 0)	0 (0 to 1)	7.19 (1.02 to 27.39)	0.59 (0.29 to 1.08)	0.27 (0.08 to 0.66)	-1.22 (-1.93 to -0.5)
**Pakistan**	**Anemia**	389 (122 to 1752)	6831 (616 to 32962)	16.58 (1.19 to 104)	38.67 (30.6 to 49.65)	45.53 (26 to 72.97)	0.49 (0.42 to 0.56)
**Pakistan**	Mild anemia	171 (54 to 768)	3315 (305 to 15544)	18.36 (1.33 to 120.16)	17.12 (13.92 to 21.95)	22.1 (14.57 to 33.43)	0.86 (0.81 to 0.9)
**Pakistan**	Moderate anemia	190 (58 to 825)	3214 (282 to 16521)	15.88 (1.02 to 101.86)	18.87 (13.95 to 25.64)	21.44 (10.29 to 38.06)	0.38 (0.23 to 0.52)
**Pakistan**	Severe anemia	27 (7 to 123)	301 (23 to 1620)	10.19 (0.16 to 81.27)	2.68 (1.56 to 4.25)	2 (0.83 to 4.07)	-1.36 (-1.66 to -1.06)
**Palau**	**Anemia**	0 (0 to 0)	1 (0 to 4)	14.77 (1.12 to 43.72)	36.4 (21.58 to 52.06)	42.81 (22.37 to 70.91)	0.36 (0.13 to 0.59)
**Palau**	Mild	0	1	16.07	22.17	29.02	0.68

	anemia	(0 to 0)	(0 to 3)	(1.42 to 53.98)	(13.5 to 32.97)	(15.38 to 47.35)	(0.43 to 0.92)
**Palau**	Moderate anemia	0 (0 to 0)	0 (0 to 1)	15.6 (1.39 to 54.39)	10.88 (5.16 to 19)	13.32 (6.05 to 23.78)	0.21 (-0.18 to 0.59)
**Palau**	Severe anemia	0 (0 to 0)	0 (0 to 0)	1.18 (-0.81 to 28.96)	3.35 (0.34 to 10.39)	0.46 (0.17 to 1)	-4.62 (-5.99 to -3.23)
**Palestine**	**Anemia**	1 (1 to 2)	32 (15 to 61)	32.26 (19.42 to 50.96)	25.82 (19.37 to 32.33)	44.21 (34.73 to 50.52)	2.19 (1.74 to 2.64)
**Palestine**	Mild anemia	1 (0 to 1)	23 (11 to 44)	34.59 (21.08 to 54.8)	17.49 (12.83 to 22.78)	32.03 (25.61 to 36.7)	2.49 (2.17 to 2.81)
**Palestine**	Moderate anemia	0 (0 to 1)	9 (4 to 17)	27.9 (14.29 to 49.31)	7.99 (5.29 to 10.58)	11.88 (7.62 to 15.43)	1.59 (0.85 to 2.33)
**Palestine**	Severe anemia	0 (0 to 0)	0 (0 to 0)	15.57 (5.62 to 42.62)	0.34 (0.17 to 0.6)	0.29 (0.15 to 0.5)	-0.31 (-1.13 to 0.51)
**Panama**	**Anemia**	164 (91 to 276)	909 (542 to 1346)	4.54 (3.06 to 6.6)	35.43 (31.44 to 40.26)	23.42 (19.69 to 28.19)	-1.74 (-2.19 to -1.28)
**Panama**	Mild anemia	93 (49 to 160)	592 (337 to 929)	5.38 (3.35 to 8.45)	20.01 (16.36 to 24.11)	15.24 (11.81 to 19.87)	-1.25 (-1.68 to -0.82)
**Panama**	Moderate anemia	67 (37 to 115)	305 (172 to 477)	3.52 (1.97 to 5.66)	14.57 (12.04 to 17.15)	7.86 (5.63 to 9.92)	-2.43 (-2.94 to -1.91)
**Panama**	Severe anemia	4 (2 to 9)	13 (5 to 27)	2.17 (0.3 to 7.03)	0.85 (0.42 to 1.48)	0.32 (0.13 to 0.62)	-3.42 (-3.86 to -2.97)
**Papua New Guinea**	**Anemia**	21 (6 to 51)	5435 (146 to 18065)	260.71 (22.56 to 583.07)	87 (78.31 to 92.18)	67.18 (52.91 to 77.03)	-0.89 (-1.07 to -0.7)
**Papua New Guinea**	Mild anemia	8 (2 to 20)	2502 (67 to 8102)	315.85 (28.47 to 767.03)	33 (26.1 to 56.65)	31.21 (24.1 to 37.89)	-0.15 (-0.35 to 0.06)
**Papua New Guinea**	Moderate anemia	11 (3 to 29)	2677 (69 to 8981)	233.17 (18.48 to 589.76)	48 (26.93 to 54.45)	32.83 (24.79 to 39.55)	-1.34 (-1.52 to -1.15)
**Papua New Guinea**	Severe anemia	1 (0 to 4)	256 (6 to 883)	176.76 (11.89 to 545.11)	5.99 (3.09 to 9.32)	3.13 (1.77 to 5.26)	-2.14 (-2.28 to -2.01)
**Paraguay**	**Anemia**	72 (47 to 120)	833 (479 to 1189)	10.54 (6.73 to 15.48)	47.05 (41.46 to 52.19)	50.33 (47.19 to 53.92)	0.51 (0.38 to 0.64)

**Paraguay**	Mild anemia	41 (25 to 70)	510 (283 to 748)	11.49 (6.96 to 17.38)	26.56 (22.06 to 31.33)	30.78 (26.11 to 35.84)	0.72 (0.6 to 0.83)
**Paraguay**	Moderate anemia	29 (19 to 49)	303 (168 to 456)	9.38 (5.53 to 15.51)	19.06 (15.73 to 22.57)	18.31 (14.27 to 21.92)	0.25 (0.1 to 0.4)
**Paraguay**	Severe anemia	2 (1 to 4)	20 (7 to 42)	8.35 (2.28 to 24.01)	1.42 (0.77 to 2.52)	1.24 (0.52 to 2.73)	-0.45 (-0.63 to -0.26)
**Peru**	**Anemia**	1053 (668 to 1680)	2951 (1807 to 4320)	1.8 (0.99 to 2.8)	34.74 (30.37 to 39.52)	34.48 (30.33 to 38.97)	-0.02 (-0.18 to 0.13)
**Peru**	Mild anemia	584 (360 to 930)	2067 (1250 to 3071)	2.54 (1.52 to 3.96)	19.24 (16.15 to 22.44)	24.14 (20.8 to 27.6)	0.89 (0.7 to 1.08)
**Peru**	Moderate anemia	430 (270 to 675)	852 (539 to 1226)	0.98 (0.41 to 1.75)	14.19 (12.15 to 16.43)	9.98 (8.73 to 11.42)	-1.43 (-1.64 to -1.23)
**Peru**	Severe anemia	39 (21 to 68)	32 (19 to 47)	-0.2 (-0.51 to 0.39)	1.3 (0.84 to 1.88)	0.37 (0.3 to 0.45)	-4.44 (-4.82 to -4.05)
**Philippines**	**Anemia**	NA	22635 (9694 to 42275)	NA	NA	28.89 (22.93 to 33.91)	NA
**Philippines**	Mild anemia	NA	15920 (6625 to 29449)	NA	NA	20.33 (16.17 to 24.12)	NA
**Philippines**	Moderate anemia	NA	6406 (2534 to 12343)	NA	NA	8.16 (5.4 to 10.78)	NA
**Philippines**	Severe anemia	NA	310 (96 to 685)	NA	NA	0.4 (0.18 to 0.72)	NA
**Poland**	**Anemia**	67 (31 to 127)	272 (164 to 420)	3.08 (1.52 to 5.74)	36.06 (28.43 to 43.28)	43.98 (36.96 to 50.59)	0.68 (0.53 to 0.83)
**Poland**	Mild anemia	47 (22 to 90)	201 (116 to 314)	3.29 (1.7 to 6.22)	25.34 (20.36 to 30.08)	32.47 (27.09 to 37.82)	0.81 (0.66 to 0.96)
**Poland**	Moderate anemia	19 (8 to 38)	66 (37 to 108)	2.48 (0.93 to 5.35)	10.21 (6.92 to 13.66)	10.62 (7.45 to 14.31)	0.18 (0 to 0.36)
**Poland**	Severe anemia	1 (0 to 2)	5 (1 to 11)	4.63 (0.31 to 16.38)	0.51 (0.26 to 0.83)	0.88 (0.18 to 2)	3.01 (1.78 to 4.24)
**Portugal**	**Anemia**	502 (249 to	345 (135 to	-0.31 (-0.74 to	21.99 (15.38 to	34.25 (13.21 to	2.25 (1.57 to

		851)	552)	0.4)	30.43)	47.93)	2.94)
**Portugal**	Mild anemia	415 (201 to 716)	285 (114 to 458)	-0.31 (-0.73 to 0.44)	18.14 (12.51 to 26.06)	28.27 (11.17 to 40.39)	2.27 (1.59 to 2.96)
**Portugal**	Moderate anemia	86 (40 to 148)	59 (20 to 104)	-0.31 (-0.78 to 0.58)	3.78 (2.11 to 5.55)	5.89 (1.83 to 9.79)	2.18 (1.48 to 2.88)
**Portugal**	Severe anemia	2 (0 to 4)	1 (0 to 3)	-0.43 (-0.93 to 1.56)	0.07 (0.02 to 0.16)	0.09 (0.01 to 0.25)	1.56 (0.93 to 2.2)
**Puerto Rico**	**Anemia**	591 (363 to 828)	144 (94 to 206)	-0.76 (-0.82 to -0.67)	39.08 (33.99 to 45.06)	42.81 (37.38 to 49.2)	0.31 (-0.01 to 0.63)
**Puerto Rico**	Mild anemia	398 (237 to 576)	100 (62 to 145)	-0.75 (-0.83 to -0.64)	26.33 (20.88 to 32.21)	29.8 (23.78 to 36.83)	0.46 (0.16 to 0.76)
**Puerto Rico**	Moderate anemia	186 (104 to 277)	43 (23 to 69)	-0.77 (-0.87 to -0.63)	12.3 (8.92 to 15.46)	12.68 (7.59 to 17.64)	0.02 (-0.36 to 0.4)
**Puerto Rico**	Severe anemia	7 (3 to 15)	1 (0 to 3)	-0.84 (-0.95 to -0.53)	0.45 (0.18 to 0.86)	0.33 (0.11 to 0.78)	-1 (-1.53 to -0.45)
**Qatar**	**Anemia**	1 (1 to 2)	2 (2 to 3)	0.71 (0.19 to 1.46)	46.3 (39.67 to 52.23)	18.18 (14.24 to 23.6)	-3.99 (-4.67 to -3.3)
**Qatar**	Mild anemia	1 (0 to 1)	2 (1 to 2)	1.13 (0.34 to 2.54)	28.39 (20.1 to 36.02)	13.88 (9.77 to 18.9)	-3.26 (-3.89 to -2.64)
**Qatar**	Moderate anemia	0 (0 to 1)	1 (0 to 1)	0.21 (-0.32 to 1.27)	15.1 (10.04 to 19.07)	4.2 (2.41 to 6.69)	-5.23 (-6.17 to -4.29)
**Qatar**	Severe anemia	0 (0 to 0)	0 (0 to 0)	-0.83 (-0.97 to 0.78)	2.81 (0.28 to 10.21)	0.11 (0.04 to 0.25)	-10.05 (-10.71 to -9.37)
**Republic of Korea**	**Anemia**	150 (0 to 284)	311 (206 to 457)	1.06 (0 to 1.75)	29.51 (0 to 38.06)	29 (24.37 to 35.8)	-0.95 (-1.23 to -0.66)
**Republic of Korea**	Mild anemia	77 (0 to 147)	219 (142 to 319)	1.86 (0 to 2.92)	15.02 (0 to 19.81)	20.46 (16.38 to 25.4)	0.22 (-0.14 to 0.59)
**Republic of Korea**	Moderate anemia	67 (0 to 127)	87 (54 to 135)	0.3 (-0.23 to 0.87)	13.17 (0 to 17.48)	8.14 (6 to 10.89)	-2.72 (-3.02 to -2.41)
**Republic of Korea**	Severe anemia	7 (0 to 14)	4 (2 to 8)	-0.36 (-0.72 to 0.16)	1.32 (0 to 2.1)	0.4 (0.23 to 0.65)	-5.02 (-5.41 to -4.63)
**Republic of**	**Anemia**	26	200	6.67	29.76	72.27	3.48

**Moldova**		(10 to 63)	(124 to 291)	(2.59 to 17.51)	(24.94 to 35.29)	(66.82 to 77.91)	(3.33 to 3.64)
**Republic of Moldova**	Mild anemia	16 (6 to 39)	129 (77 to 198)	6.84 (2.51 to 18.79)	18.75 (14.74 to 23.45)	46.56 (39.17 to 55.5)	3.58 (3.43 to 3.73)
**Republic of Moldova**	Moderate anemia	9 (4 to 22)	69 (38 to 108)	6.51 (2.18 to 17.57)	10.49 (7.82 to 13.17)	24.97 (17.1 to 31.58)	3.75 (3.48 to 4.03)
**Republic of Moldova**	Severe anemia	0 (0 to 1)	2 (1 to 4)	3.58 (0.29 to 16.98)	0.51 (0.24 to 0.89)	0.74 (0.31 to 1.44)	-2.36 (-3.92 to -0.78)
**Romania**	**Anemia**	185 (110 to 298)	842 (550 to 1136)	3.55 (1.9 to 5.61)	65.65 (58.32 to 69.23)	47.94 (35.35 to 54.82)	-1.28 (-1.37 to -1.2)
**Romania**	Mild anemia	113 (60 to 189)	624 (413 to 847)	4.5 (2.39 to 8.04)	40.05 (31.51 to 50.08)	35.51 (27.01 to 41.79)	-0.79 (-0.93 to -0.65)
**Romania**	Moderate anemia	53 (27 to 94)	211 (117 to 308)	2.99 (1.07 to 6.04)	18.65 (13.18 to 24.04)	12.03 (7.29 to 15.94)	-1.9 (-2.07 to -1.73)
**Romania**	Severe anemia	19 (1 to 36)	7 (3 to 14)	-0.63 (-0.88 to 5.01)	6.95 (0.48 to 13.99)	0.39 (0.16 to 0.75)	-7.24 (-9.07 to -5.37)
**Russian Federation**	**Anemia**	1221 (741 to 1921)	13340 (6562 to 20420)	9.93 (5.06 to 15.85)	28.02 (19.32 to 35.58)	40.62 (23.29 to 51.38)	1.56 (1.39 to 1.72)
**Russian Federation**	Mild anemia	837 (516 to 1332)	9674 (5062 to 14788)	10.55 (5.61 to 16.35)	19.19 (13.85 to 23.93)	29.46 (17.61 to 36.76)	1.81 (1.64 to 1.98)
**Russian Federation**	Moderate anemia	358 (189 to 597)	3553 (1471 to 6281)	8.93 (3.64 to 18.16)	8.23 (4.92 to 11.89)	10.82 (5.15 to 17.31)	1.11 (0.94 to 1.27)
**Russian Federation**	Severe anemia	26 (9 to 54)	114 (37 to 292)	3.42 (0.28 to 14.3)	0.6 (0.21 to 1.32)	0.35 (0.12 to 0.8)	-1.72 (-2 to -1.43)
**Rwanda**	**Anemia**	6960 (2247 to 16155)	7460 (4904 to 10239)	0.07 (-0.52 to 2.17)	54.62 (32.32 to 75.58)	38.1 (26.02 to 49.47)	-1.32 (-1.52 to -1.11)
**Rwanda**	Mild anemia	3674 (1163 to 8589)	4585 (3223 to 6096)	0.25 (-0.44 to 2.63)	28.88 (16.7 to 40.67)	23.41 (17.32 to 29.77)	-0.65 (-0.88 to -0.43)
**Rwanda**	Moderate anemia	2985 (911 to 7392)	2716 (1528 to 4140)	-0.09 (-0.67 to 1.83)	23.36 (13.4 to 35.32)	13.88 (7.93 to 20.22)	-1.93 (-2.27 to -1.6)
**Rwanda**	Severe anemia	301 (79 to 735)	159 (75 to 299)	-0.47 (-0.82 to 1.02)	2.37 (1.04 to 4.34)	0.81 (0.38 to 1.46)	-4.43 (-4.99 to -3.86)

**Saint Kitts and Nevis**	**Anemia**	4 (1 to 7)	18 (6 to 40)	4 (0.52 to 11.14)	37.41 (29.67 to 46.52)	46.46 (34.41 to 69.12)	0.6 (0.49 to 0.71)
**Saint Kitts and Nevis**	Mild anemia	2 (1 to 4)	11 (4 to 26)	4.09 (0.5 to 11.3)	23.85 (18.48 to 30.1)	30.1 (22.03 to 43.19)	0.71 (0.6 to 0.81)
**Saint Kitts and Nevis**	Moderat e anemia	1 (0 to 2)	6 (2 to 14)	3.9 (0.45 to 11.29)	12.96 (9.26 to 17.14)	15.83 (9.54 to 25.14)	0.44 (0.29 to 0.59)
**Saint Kitts and Nevis**	Severe anemia	0 (0 to 0)	0 (0 to 1)	2.57 (-0.19 to 11.4)	0.59 (0.29 to 1.09)	0.53 (0.21 to 1.13)	-0.29 (-0.55 to -0.02)
**Saint Lucia**	**Anemia**	8 (5 to 12)	16 (9 to 24)	0.9 (0.45 to 1.45)	50.94 (46.16 to 55.59)	58.55 (54.19 to 63.29)	0.33 (0.25 to 0.42)
**Saint Lucia**	Mild anemia	5 (3 to 7)	9 (5 to 15)	0.9 (0.34 to 1.74)	29.7 (24.9 to 34.47)	34.1 (27.34 to 44.42)	0.39 (0.28 to 0.5)
**Saint Lucia**	Moderat e anemia	3 (2 to 5)	6 (3 to 10)	0.92 (0.11 to 1.77)	20.17 (16.75 to 23.63)	23.49 (14.68 to 28.89)	0.29 (0.15 to 0.43)
**Saint Lucia**	Severe anemia	0 (0 to 0)	0 (0 to 1)	0.46 (-0.48 to 2.77)	1.07 (0.54 to 1.98)	0.96 (0.37 to 1.99)	-0.69 (-1.04 to -0.34)
**Saint Vincent and the Grenadines**	**Anemia**	21 (12 to 34)	28 (17 to 41)	0.32 (-0.05 to 0.81)	52.68 (47.5 to 57.39)	55.97 (51.01 to 60.85)	0.1 (0.02 to 0.19)
**Saint Vincent and the Grenadines**	Mild anemia	12 (6 to 20)	16 (9 to 25)	0.31 (-0.1 to 0.86)	30.47 (25.58 to 35.54)	32.08 (26.39 to 38.11)	0.09 (0.01 to 0.17)
**Saint Vincent and the Grenadines**	Moderat e anemia	8 (5 to 14)	11 (7 to 17)	0.35 (-0.1 to 0.96)	21.12 (17.32 to 24.99)	22.84 (17.67 to 27.03)	0.14 (0.05 to 0.24)
**Saint Vincent and the Grenadines**	Severe anemia	0 (0 to 1)	1 (0 to 1)	0.2 (-0.5 to 2.03)	1.09 (0.56 to 1.87)	1.05 (0.46 to 2.17)	-0.33 (-0.45 to -0.2)
**Samoa**	**Anemia**	4 (1 to 29)	25 (2 to 131)	5.09 (0.08 to 39.69)	62.17 (37.29 to 84.13)	52.3 (29.43 to 80.69)	-0.62 (-0.75 to -0.49)
**Samoa**	Mild anemia	3 (0 to 18)	17 (1 to 89)	5.37 (0.06 to 41.68)	39.53 (25.27 to 54.73)	35.05 (20 to 54.53)	-0.45 (-0.58 to -0.32)
**Samoa**	Moderat e anemia	1 (0 to 9)	8 (1 to 44)	4.65 (-0.08 to 36.28)	21.56 (11.43 to 30.96)	16.43 (7.92 to 26.4)	-0.91 (-1.04 to -0.78)
**Samoa**	Severe anemia	0 (0 to 0)	0 (0 to 2)	3.7 (-0.38 to	1.08 (0.47 to	0.81 (0.25 to	-1.45 (-1.65 to

				37.47)	1.99)	1.48)	-1.24)
**San Marino**	**Anemia**	0 (0 to 1)	0 (0 to 1)	0.05 (-0.69 to 5.79)	30.39 (13.6 to 57.85)	38.36 (13.21 to 61.36)	0.49 (0.21 to 0.78)
**San Marino**	Mild anemia	0 (0 to 1)	0 (0 to 1)	0.05 (-0.67 to 6.18)	25.2 (11.59 to 48.16)	32.01 (11.22 to 51.29)	0.51 (0.22 to 0.8)
**San Marino**	Moderat e anemia	0 (0 to 0)	0 (0 to 0)	0.04 (-0.72 to 6.53)	5.03 (1.87 to 10.59)	6.28 (2.14 to 10.79)	0.46 (0.19 to 0.73)
**San Marino**	Severe anemia	0 (0 to 0)	0 (0 to 0)	-0.38 (-0.95 to 10.05)	0.16 (0.01 to 1.24)	0.07 (0.01 to 0.22)	-1.68 (-2.34 to -1.03)
**Sao Tome and Principe**	**Anemia**	2 (2 to 3)	1 (1 to 1)	-0.54 (-0.72 to -0.3)	37.3 (31.25 to 41.01)	44.98 (37.42 to 50.61)	1.22 (0.92 to 1.51)
**Sao Tome and Principe**	Mild anemia	1 (1 to 1)	1 (0 to 1)	-0.49 (-0.7 to -0.2)	18.72 (15.33 to 21.49)	24.88 (19.27 to 29.8)	1.39 (1.2 to 1.58)
**Sao Tome and Principe**	Moderat e anemia	1 (1 to 1)	0 (0 to 1)	-0.58 (-0.75 to -0.32)	17.08 (13.13 to 19.53)	18.68 (14.74 to 22.74)	1.1 (0.67 to 1.52)
**Sao Tome and Principe**	Severe anemia	0 (0 to 0)	0 (0 to 0)	-0.64 (-0.88 to 0.55)	1.5 (0.79 to 2.4)	1.43 (0.58 to 5.87)	0.14 (-0.38 to 0.66)
**Saudi Arabia**	**Anemia**	64 (32 to 134)	274 (79 to 669)	3.28 (0.46 to 10.31)	26.54 (18.4 to 37.55)	28.56 (13.77 to 54.18)	0.58 (-0.45 to 1.63)
**Saudi Arabia**	Mild anemia	44 (22 to 93)	196 (60 to 497)	3.49 (0.5 to 11.05)	18.11 (13.19 to 24.4)	20.13 (10.51 to 46.14)	1.13 (0.1 to 2.17)
**Saudi Arabia**	Moderat e anemia	19 (8 to 41)	57 (16 to 150)	1.95 (-0.06 to 7.71)	8.02 (4.63 to 12.47)	5.82 (2.84 to 12.67)	-1.03 (-2.29 to 0.25)
**Saudi Arabia**	Severe anemia	1 (0 to 3)	21 (0 to 92)	19.98 (-0.53 to 142.15)	0.42 (0.16 to 0.87)	2.6 (0.06 to 13.73)	0.72 (-1.03 to 2.5)
**Senegal**	**Anemia**	1465 (844 to 2432)	3132 (2229 to 4357)	1.14 (0.29 to 2.66)	90.36 (83.86 to 93.58)	64.23 (58.86 to 69.9)	-1.29 (-1.44 to -1.14)
**Senegal**	Mild anemia	535 (296 to 908)	1406 (912 to 2029)	1.63 (0.5 to 3.91)	32.96 (27.84 to 38.17)	28.86 (22.76 to 36.01)	-0.62 (-0.74 to -0.49)
**Senegal**	Moderat e anemia	822 (468 to 1364)	1610 (1030 to 2360)	0.96 (0.14 to 2.36)	50.73 (45.1 to 54.36)	32.98 (25.86 to 38.94)	-1.53 (-1.77 to -1.29)
**Senegal**	Severe	108	116	0.08	6.66	2.39	-3.64

	anemia	(49 to 208)	(66 to 192)	(-0.51 to 1.73)	(3.7 to 10.44)	(1.49 to 3.52)	(-4.13 to -3.14)
**Serbia**	**Anemia**	42 (0 to 86)	18 (11 to 28)	-0.58 (-0.79 to 0)	32.25 (0 to 49.84)	22.05 (16.94 to 27.64)	-1.89 (-2.34 to -1.45)
**Serbia**	Mild anemia	29 (0 to 60)	14 (8 to 22)	-0.53 (-0.78 to 0)	22.25 (0 to 35.33)	16.93 (12.7 to 21.41)	-1.23 (-1.72 to -0.73)
**Serbia**	Moderate anemia	13 (0 to 26)	4 (2 to 6)	-0.68 (-0.86 to 0)	9.55 (0 to 16.69)	4.94 (3.45 to 6.54)	-3.15 (-3.59 to -2.71)
**Serbia**	Severe anemia	1 (0 to 1)	0 (0 to 0)	-0.75 (-0.92 to 0)	0.44 (0 to 1.05)	0.18 (0.09 to 0.26)	-7.57 (-9.41 to -5.68)
**Seychelles**	**Anemia**	1 (1 to 2)	6 (4 to 9)	4.22 (2.88 to 5.68)	51.94 (47.48 to 56.28)	58.03 (52.84 to 62.06)	0.48 (0.29 to 0.67)
**Seychelles**	Mild anemia	1 (0 to 1)	4 (2 to 6)	4.94 (3.26 to 7.1)	31.13 (25.57 to 36.88)	39.57 (33.19 to 45.79)	0.91 (0.71 to 1.1)
**Seychelles**	Moderate anemia	0 (0 to 1)	2 (1 to 3)	3.2 (1.79 to 5.31)	19.86 (14.73 to 24.17)	17.9 (12.65 to 23.11)	-0.22 (-0.42 to -0.01)
**Seychelles**	Severe anemia	0 (0 to 0)	0 (0 to 0)	1.71 (0.09 to 6.21)	0.95 (0.45 to 1.74)	0.56 (0.24 to 1.1)	-1.77 (-1.9 to -1.64)
**Sierra Leone**	**Anemia**	541 (158 to 1454)	6767 (4274 to 10315)	11.51 (3.32 to 40.43)	57.86 (42.59 to 71.17)	55.17 (39.56 to 68.67)	-0.15 (-0.35 to 0.04)
**Sierra Leone**	Mild anemia	235 (70 to 610)	3660 (2360 to 5381)	14.57 (4.53 to 50.75)	25.17 (20.81 to 30.27)	29.75 (24.07 to 35.58)	0.58 (0.4 to 0.76)
**Sierra Leone**	Moderate anemia	283 (79 to 809)	2939 (1552 to 4808)	9.39 (2.2 to 37.52)	30.22 (17.69 to 41.77)	24.04 (14.37 to 35.3)	-0.73 (-0.95 to -0.5)
**Sierra Leone**	Severe anemia	23 (5 to 70)	168 (88 to 288)	6.3 (0.91 to 33.13)	2.46 (0.99 to 5.04)	1.38 (0.79 to 2.27)	-2.08 (-2.32 to -1.85)
**Singapore**	**Anemia**	15 (7 to 26)	19 (11 to 30)	0.26 (-0.22 to 1.02)	25.59 (20.2 to 32.06)	31.08 (25.11 to 37.35)	0.77 (0.36 to 1.19)
**Singapore**	Mild anemia	11 (5 to 18)	15 (8 to 23)	0.36 (-0.17 to 1.24)	18.05 (13.18 to 23.21)	23.65 (18.61 to 28.88)	1.09 (0.68 to 1.5)
**Singapore**	Moderate anemia	4 (2 to 7)	4 (2 to 7)	0.04 (-0.39 to 0.83)	7.22 (5.04 to 9.78)	7.22 (5.23 to 9.8)	-0.03 (-0.48 to 0.42)

**Singapore**	Severe anemia	0 (0 to 0)	0 (0 to 0)	-0.32 (-0.72 to 0.71)	0.33 (0.16 to 0.61)	0.22 (0.1 to 0.39)	-1.6 (-1.94 to -1.26)
**Slovakia**	**Anemia**	1 (0 to 2)	9 (6 to 13)	5.21 (0 to 14.75)	46.9 (0 to 58.46)	52.86 (38.83 to 58.4)	0.28 (0.21 to 0.34)
**Slovakia**	Mild anemia	1 (0 to 2)	7 (4 to 10)	7.14 (0 to 22.18)	26.54 (0 to 39.38)	39.24 (29.28 to 45.29)	0.73 (0.49 to 0.97)
**Slovakia**	Moderate anemia	1 (0 to 1)	2 (1 to 3)	2.92 (0 to 12.32)	18.43 (0 to 28.97)	13.08 (7.92 to 17.56)	-0.87 (-1.2 to -0.54)
**Slovakia**	Severe anemia	0 (0 to 0)	0 (0 to 0)	0.55 (-0.89 to 8.78)	1.93 (0 to 14.57)	0.54 (0.16 to 0.71)	1.21 (-1.48 to 3.97)
**Slovenia**	**Anemia**	1 (0 to 1)	2 (1 to 3)	2.35 (0 to 5.45)	24.24 (0 to 32.98)	31.7 (25.54 to 38.48)	0.76 (0.43 to 1.09)
**Slovenia**	Mild anemia	0 (0 to 1)	2 (1 to 3)	2.44 (0 to 5.72)	18.94 (0 to 25.74)	25.42 (20.37 to 31.54)	0.91 (0.59 to 1.22)
**Slovenia**	Moderate anemia	0 (0 to 0)	0 (0 to 1)	2.08 (0 to 5.7)	5.1 (0 to 7.72)	6.12 (4.53 to 8.01)	0.29 (-0.1 to 0.69)
**Slovenia**	Severe anemia	0 (0 to 0)	0 (0 to 0)	1.04 (-0.19 to 4.53)	0.2 (0 to 0.37)	0.16 (0.07 to 0.27)	-1.88 (-2.77 to -0.99)
**Solomon Islands**	**Anemia**	11 (1 to 68)	104 (6 to 682)	8.85 (0.43 to 71.43)	69.6 (44.1 to 87.51)	61.23 (36.58 to 86.58)	-0.53 (-0.65 to -0.41)
**Solomon Islands**	Mild anemia	5 (1 to 33)	58 (3 to 362)	10.37 (0.67 to 82.99)	33.47 (22.8 to 43.12)	33.84 (21.54 to 48.11)	-0.08 (-0.24 to 0.08)
**Solomon Islands**	Moderate anemia	5 (1 to 33)	44 (2 to 268)	7.64 (0.3 to 64.56)	33.47 (19.97 to 43.95)	25.95 (14.14 to 38.52)	-0.92 (-1.01 to -0.83)
**Solomon Islands**	Severe anemia	0 (0 to 3)	2 (0 to 15)	4.79 (-0.23 to 46.77)	2.66 (1.24 to 4.7)	1.43 (0.6 to 2.89)	-2.12 (-2.25 to -1.99)
**Somalia**	**Anemia**	226 (75 to 661)	3725 (2355 to 5954)	15.52 (4.58 to 50.58)	88.58 (82.41 to 92.69)	54.43 (48.97 to 60.65)	-2.2 (-2.49 to -1.9)
**Somalia**	Mild anemia	73 (23 to 216)	1501 (920 to 2363)	19.58 (5.86 to 64.95)	28.6 (24.53 to 33.21)	21.93 (18.49 to 25.72)	-1.45 (-1.78 to -1.11)
**Somalia**	Moderate anemia	130 (44 to	2009 (1259 to	14.43 (4.07 to	51.18 (46.81 to	29.38 (26 to	-2.39 (-2.65 to

		387)	3204)	46.37)	54.72)	33.1)	-2.12)
**Somalia**	Severe anemia	22 (6 to 65)	214 (101 to 402)	8.59 (1.6 to 34.98)	8.8 (5.28 to 12.68)	3.12 (1.89 to 4.89)	-4.35 (-4.72 to -3.98)
**South Africa**	**Anemia**	28701 (22813 to 35493)	228594 (175582 to 297183)	6.96 (4.75 to 10.33)	87.23 (79.36 to 90.47)	35.88 (27.11 to 45.05)	-2.78 (-3.28 to -2.28)
**South Africa**	Mild anemia	13929 (10277 to 18250)	118800 (86665 to 157296)	7.53 (4.67 to 11.81)	42.32 (34.69 to 49.64)	18.68 (13.33 to 24.54)	-2.7 (-3.29 to -2.11)
**South Africa**	Moderate anemia	13361 (9892 to 17362)	102020 (67635 to 140941)	6.64 (3.84 to 10.8)	40.62 (32.75 to 47.14)	15.98 (10.76 to 21.71)	-2.78 (-3.22 to -2.33)
**South Africa**	Severe anemia	1411 (734 to 2420)	7775 (4617 to 12609)	4.51 (1.63 to 11.05)	4.3 (2.32 to 7.09)	1.22 (0.74 to 1.9)	-3.69 (-3.99 to -3.39)
**South Sudan**	**Anemia**	1383 (322 to 4890)	9213 (2974 to 20985)	5.66 (0.4 to 35.82)	83.34 (70.4 to 91.07)	65.47 (50.14 to 79.81)	-1.05 (-1.13 to -0.97)
**South Sudan**	Mild anemia	600 (131 to 2165)	4321 (1412 to 10223)	6.2 (0.5 to 41.34)	35.86 (28.87 to 44.43)	30.58 (23.25 to 38.26)	-0.76 (-0.85 to -0.68)
**South Sudan**	Moderate anemia	713 (165 to 2383)	4514 (1460 to 9984)	5.33 (0.33 to 32.46)	43.22 (33.2 to 51.4)	32.19 (22.7 to 40.71)	-1.21 (-1.28 to -1.13)
**South Sudan**	Severe anemia	71 (13 to 269)	378 (98 to 958)	4.32 (-0.09 to 35.43)	4.26 (2.02 to 7.51)	2.69 (1.35 to 4.96)	-1.97 (-2.14 to -1.8)
**Spain**	**Anemia**	4158 (3121 to 5326)	681 (457 to 937)	-0.84 (-0.89 to -0.76)	28.39 (23.16 to 34.53)	38.8 (29.32 to 46.19)	1.15 (0.86 to 1.45)
**Spain**	Mild anemia	3327 (2473 to 4492)	573 (381 to 798)	-0.83 (-0.89 to -0.74)	22.71 (17.87 to 28.99)	32.61 (24.56 to 39.3)	1.3 (1 to 1.61)
**Spain**	Moderate anemia	818 (472 to 1293)	108 (63 to 165)	-0.87 (-0.93 to -0.76)	5.59 (3.34 to 8.49)	6.13 (3.9 to 8.69)	0.46 (0.21 to 0.71)
**Spain**	Severe anemia	13 (4 to 33)	1 (0 to 3)	-0.91 (-0.98 to -0.66)	0.09 (0.03 to 0.24)	0.07 (0.02 to 0.17)	-0.98 (-1.14 to -0.82)
**Sri Lanka**	**Anemia**	38 (21 to 62)	130 (80 to 191)	2.42 (1.19 to 5.58)	53.55 (49.14 to 58)	40.32 (36.47 to 44.64)	-0.84 (-0.94 to -0.75)
**Sri Lanka**	Mild anemia	21 (11 to 34)	92 (56 to 136)	3.42 (1.73 to 7.4)	29.33 (25.77 to 32.75)	28.53 (24.3 to 33.11)	0.11 (0 to 0.21)

**Sri Lanka**	Moderate anemia	16 (9 to 27)	37 (21 to 57)	1.29 (0.36 to 3.6)	22.82 (20.42 to 25.16)	11.51 (8.78 to 14.23)	-2.34 (-2.41 to -2.26)
**Sri Lanka**	Severe anemia	1 (0 to 2)	1 (0 to 2)	-0.05 (-0.63 to 1.29)	1.4 (0.96 to 2.01)	0.29 (0.14 to 0.52)	-5.46 (-5.68 to -5.24)
**Sudan**	**Anemia**	856 (142 to 3033)	8402 (2041 to 25167)	8.82 (0.29 to 106.54)	73.5 (56.31 to 89.61)	70.36 (52.83 to 84.07)	-0.01 (-0.07 to 0.05)
**Sudan**	Mild anemia	384 (62 to 1346)	4448 (1192 to 13553)	10.57 (0.51 to 131.52)	33.05 (26.86 to 39.48)	37 (27.96 to 46.37)	0.45 (0.37 to 0.53)
**Sudan**	Moderate anemia	432 (70 to 1560)	3749 (967 to 11350)	7.68 (0.14 to 101.51)	37.08 (25.92 to 47.63)	31.62 (19.83 to 40.53)	-0.32 (-0.43 to -0.2)
**Sudan**	Severe anemia	39 (5 to 148)	205 (39 to 672)	4.2 (-0.45 to 65.52)	3.37 (1.44 to 6.14)	1.74 (0.68 to 3.54)	-1.98 (-2.17 to -1.79)
**Suriname**	**Anemia**	57 (33 to 109)	241 (149 to 374)	3.25 (1.53 to 5.46)	52.24 (46.7 to 57.14)	71.5 (68.4 to 74.5)	0.86 (0.65 to 1.06)
**Suriname**	Mild anemia	31 (18 to 60)	133 (77 to 220)	3.26 (1.35 to 5.78)	28.75 (24.16 to 33.7)	39.41 (32.85 to 47.18)	0.85 (0.68 to 1.02)
**Suriname**	Moderate anemia	24 (13 to 46)	103 (60 to 166)	3.29 (1.49 to 5.86)	22.13 (18.03 to 25.79)	30.56 (23.93 to 35.91)	0.9 (0.65 to 1.16)
**Suriname**	Severe anemia	1 (1 to 3)	5 (2 to 11)	2.44 (0.33 to 7.65)	1.36 (0.68 to 2.34)	1.53 (0.69 to 2.89)	0.14 (-0.18 to 0.46)
**Sweden**	**Anemia**	32 (12 to 62)	51 (31 to 77)	0.58 (-0.06 to 2.49)	23.1 (11.35 to 34.54)	36.22 (26.75 to 43.12)	1.5 (1.15 to 1.85)
**Sweden**	Mild anemia	27 (10 to 52)	42 (26 to 64)	0.56 (-0.06 to 2.36)	19.41 (9.69 to 28.7)	30.11 (23.38 to 36.44)	1.39 (1.05 to 1.73)
**Sweden**	Moderate anemia	5 (2 to 11)	9 (5 to 14)	0.67 (-0.17 to 3.67)	3.65 (1.45 to 6.68)	6.03 (3.61 to 8.28)	2.03 (1.64 to 2.43)
**Sweden**	Severe anemia	0 (0 to 0)	0 (0 to 0)	0.65 (-0.7 to 10.14)	0.05 (0.01 to 0.17)	0.08 (0.01 to 0.22)	1.8 (-0.18 to 3.81)
**Switzerland**	**Anemia**	349 (170 to 543)	107 (61 to 158)	-0.69 (-0.83 to -0.41)	37.25 (19.9 to 45.38)	44.51 (29.09 to 51.04)	0.63 (0.36 to 0.91)
**Switzerland**	Mild anemia	288 (146 to	89 (52 to	-0.69 (-0.82 to	30.8 (16.74 to	36.93 (24.74 to	0.64 (0.36 to

		454)	131)	-0.42)	38.56)	43.09)	0.92)
**Switzerland**	Moderate anemia	60 (22 to 109)	18 (9 to 29)	-0.7 (-0.85 to -0.3)	6.37 (2.77 to 10.25)	7.49 (4.33 to 10.42)	0.63 (0.38 to 0.88)
**Switzerland**	Severe anemia	1 (0 to 2)	0 (0 to 1)	-0.7 (-0.95 to 0.6)	0.08 (0.01 to 0.23)	0.09 (0.02 to 0.25)	0.26 (0.01 to 0.51)
**Syrian Arab Republic**	**Anemia**	16 (12 to 21)	44 (21 to 79)	1.83 (0.66 to 3.34)	59.69 (52.24 to 64.44)	36.41 (24.64 to 43.19)	-1.42 (-1.77 to -1.06)
**Syrian Arab Republic**	Mild anemia	8 (6 to 12)	33 (16 to 60)	2.96 (1.21 to 5.31)	31.52 (25.91 to 38.3)	26.95 (18.55 to 32.74)	-0.49 (-0.87 to -0.1)
**Syrian Arab Republic**	Moderate anemia	7 (5 to 10)	11 (5 to 21)	0.59 (-0.17 to 1.71)	26.54 (20.02 to 31.96)	9.12 (5.31 to 13.67)	-2.93 (-3.32 to -2.53)
**Syrian Arab Republic**	Severe anemia	0 (0 to 1)	0 (0 to 1)	-0.02 (-0.64 to 1.37)	1.62 (0.75 to 2.9)	0.34 (0.15 to 0.67)	-4.54 (-4.86 to -4.22)
**Taiwan (Province of China)**	**Anemia**	12 (6 to 19)	43 (20 to 76)	2.72 (0.86 to 5.16)	13.79 (10.27 to 19.41)	13.86 (11.25 to 17.9)	-0.76 (-1.01 to -0.5)
**Taiwan (Province of China)**	Mild anemia	8 (4 to 14)	33 (15 to 60)	2.97 (0.85 to 5.81)	9.83 (6.61 to 14.85)	10.55 (8.38 to 14.27)	-0.43 (-0.64 to -0.21)
**Taiwan (Province of China)**	Moderate anemia	3 (2 to 5)	10 (5 to 18)	2.15 (0.65 to 4.02)	3.78 (2.75 to 4.79)	3.21 (2.44 to 4.08)	-1.63 (-2 to -1.26)
**Taiwan (Province of China)**	Severe anemia	0 (0 to 0)	0 (0 to 1)	1.17 (-0.2 to 3.67)	0.18 (0.09 to 0.3)	0.11 (0.05 to 0.18)	-2.85 (-3.24 to -2.45)
**Tajikistan**	**Anemia**	30 (16 to 56)	159 (95 to 217)	4.36 (2.18 to 8.81)	68.71 (60.56 to 74.06)	78.76 (74.95 to 81.89)	0.49 (0.35 to 0.63)
**Tajikistan**	Mild anemia	15 (8 to 29)	81 (47 to 116)	4.35 (2.1 to 8.89)	35.01 (29.33 to 41)	40.13 (34.13 to 46.21)	0.44 (0.36 to 0.53)
**Tajikistan**	Moderate anemia	13 (7 to 25)	71 (41 to 100)	4.28 (2.03 to 8.93)	31.07 (23.7 to 36.13)	35.01 (30.26 to 39.16)	0.32 (0.15 to 0.5)
**Tajikistan**	Severe anemia	1 (0 to 2)	7 (2 to 17)	5.39 (0.57 to 22.35)	2.62 (1.25 to 4.62)	3.62 (1.28 to 8.18)	2.4 (1.35 to 3.45)
**Thailand**	**Anemia**	8004 (4941 to 12076)	10800 (4805 to 20153)	0.35 (-0.42 to 1.71)	39.49 (34.43 to 44.39)	26.88 (11.97 to 47.43)	-1.49 (-1.83 to -1.15)
**Thailand**	Mild	5357	8619	0.61	26.42	21.45	-0.92

	anemia	(3195 to 8022)	(3877 to 15969)	(-0.32 to 2.26)	(22.09 to 31.63)	(9.62 to 37.35)	(-1.32 to -0.52)
**Thailand**	Moderate anemia	2542 (1353 to 3975)	2113 (864 to 4443)	-0.17 (-0.68 to 0.82)	12.55 (8.26 to 15.46)	5.26 (2.19 to 10.23)	-3.1 (-3.39 to -2.81)
**Thailand**	Severe anemia	105 (42 to 220)	68 (17 to 190)	-0.36 (-0.84 to 0.98)	0.52 (0.25 to 1.02)	0.17 (0.04 to 0.44)	-4.23 (-4.72 to -3.73)
**Timor-Leste**	**Anemia**	74 (3 to 454)	355 (9 to 1827)	3.77 (0.04 to 47.07)	48.58 (22.3 to 81.03)	46.85 (28.34 to 73)	-0.53 (-0.77 to -0.3)
**Timor-Leste**	Mild anemia	43 (2 to 285)	228 (6 to 1196)	4.34 (0.12 to 53.29)	27.3 (15.54 to 42.53)	30.17 (19.19 to 46.64)	0.01 (-0.21 to 0.22)
**Timor-Leste**	Moderate anemia	30 (1 to 178)	123 (3 to 668)	3.09 (-0.19 to 44.24)	20.07 (6.43 to 36.86)	16.1 (8.07 to 26.05)	-1.43 (-1.81 to -1.05)
**Timor-Leste**	Severe anemia	2 (0 to 12)	4 (0 to 24)	1.5 (-0.58 to 29.01)	1.21 (0.36 to 2.69)	0.59 (0.27 to 1.06)	-1.17 (-2.8 to 0.49)
**Togo**	**Anemia**	1461 (457 to 3583)	6238 (4199 to 8700)	3.27 (0.8 to 11.9)	66.23 (49.87 to 79.7)	53.1 (43.08 to 61.63)	-0.84 (-1.06 to -0.62)
**Togo**	Mild anemia	671 (211 to 1666)	3298 (2063 to 4925)	3.92 (0.91 to 15.41)	30.37 (23.41 to 37.12)	28.1 (20.13 to 35.86)	-0.71 (-0.96 to -0.46)
**Togo**	Moderate anemia	740 (234 to 1842)	2797 (1864 to 4019)	2.78 (0.59 to 10.89)	33.61 (22.3 to 44.57)	23.78 (18.36 to 28.79)	-0.93 (-1.23 to -0.62)
**Togo**	Severe anemia	50 (12 to 143)	143 (84 to 225)	1.88 (-0.02 to 10.57)	2.26 (0.95 to 4.33)	1.22 (0.78 to 1.78)	-1.71 (-2.23 to -1.19)
**Tokelau**	**Anemia**	0 (0 to 0)	0 (0 to 0)	16.72 (1.98 to 48.72)	38.97 (24.92 to 51.11)	47.1 (26.75 to 74.05)	0.33 (0.02 to 0.64)
**Tokelau**	Mild anemia	0 (0 to 0)	0 (0 to 0)	18.25 (2.26 to 53.98)	22.27 (15.17 to 29.89)	29.66 (17.31 to 46.39)	0.75 (0.49 to 1)
**Tokelau**	Moderate anemia	0 (0 to 0)	0 (0 to 0)	14.99 (1.45 to 45.9)	15.66 (8.78 to 22.03)	16.73 (8.4 to 27.75)	-0.19 (-0.58 to 0.2)
**Tokelau**	Severe anemia	0 (0 to 0)	0 (0 to 0)	9.24 (0.46 to 38.6)	1.04 (0.46 to 1.94)	0.71 (0.28 to 1.45)	-1.84 (-2.24 to -1.44)
**Tonga**	**Anemia**	1 (0 to 2)	4 (2 to 7)	2.63 (0.88 to 6.39)	54.57 (48.5 to 60.11)	53.11 (47.16 to 59)	-0.03 (-0.08 to 0.02)

**Tonga**	Mild anemia	1 (0 to 1)	3 (1 to 5)	2.89 (0.99 to 7.32)	31.88 (26.63 to 37.29)	33.27 (27.85 to 38.92)	0.21 (0.16 to 0.25)
**Tonga**	Moderate anemia	0 (0 to 1)	1 (1 to 3)	2.3 (0.64 to 6.06)	21.49 (17.27 to 25.64)	18.98 (14.77 to 23.55)	-0.35 (-0.41 to -0.28)
**Tonga**	Severe anemia	0 (0 to 0)	0 (0 to 0)	1.72 (-0.05 to 7.04)	1.2 (0.65 to 1.98)	0.87 (0.42 to 1.6)	-1.02 (-1.1 to -0.93)
**Trinidad and Tobago**	**Anemia**	179 (92 to 314)	402 (301 to 560)	1.24 (0.5 to 2.92)	45.11 (39.84 to 50.36)	63.73 (59.9 to 67.95)	0.86 (0.58 to 1.15)
**Trinidad and Tobago**	Mild anemia	105 (53 to 189)	234 (164 to 359)	1.23 (0.36 to 2.99)	26.45 (21.21 to 31.83)	37.08 (30.93 to 46.55)	0.91 (0.64 to 1.18)
**Trinidad and Tobago**	Moderate anemia	70 (35 to 129)	161 (105 to 238)	1.29 (0.32 to 3.33)	17.7 (14.03 to 21.11)	25.59 (16.61 to 30.8)	0.85 (0.53 to 1.16)
**Trinidad and Tobago**	Severe anemia	4 (1 to 8)	7 (3 to 14)	0.78 (-0.38 to 3.72)	0.95 (0.47 to 1.7)	1.07 (0.44 to 2.14)	-0.12 (-0.43 to 0.19)
**Tunisia**	**Anemia**	14 (5 to 58)	206 (27 to 664)	13.23 (1.55 to 51.28)	44.73 (31.42 to 65.55)	31.61 (13.02 to 59.72)	-1.33 (-1.72 to -0.94)
**Tunisia**	Mild anemia	8 (2 to 35)	152 (17 to 503)	17.46 (2.14 to 77.96)	25.01 (14.39 to 41.97)	22.42 (9.44 to 44.25)	-0.37 (-0.93 to 0.19)
**Tunisia**	Moderate anemia	4 (1 to 19)	43 (4 to 147)	8.51 (0.29 to 40.51)	13.77 (8.41 to 22.12)	6.32 (2.25 to 13.67)	-3.07 (-3.57 to -2.56)
**Tunisia**	Severe anemia	2 (0 to 9)	12 (0 to 39)	5.66 (-0.92 to 233.5)	5.95 (0.39 to 18.73)	2.87 (0.07 to 15.01)	-4.7 (-7.64 to -1.67)
**Turkey**	**Anemia**	25 (0 to 38)	505 (271 to 850)	19.5 (0 to 24.7)	42.92 (0 to 56.1)	45.8 (42.68 to 49.81)	-0.42 (-0.59 to -0.24)
**Turkey**	Mild anemia	12 (0 to 20)	347 (178 to 616)	27.12 (0 to 36.99)	21.47 (0 to 30.8)	31.44 (24.32 to 37.75)	0.51 (0.3 to 0.72)
**Turkey**	Moderate anemia	12 (0 to 18)	153 (69 to 288)	12.33 (0 to 18.22)	20.05 (0 to 27.18)	13.91 (9.02 to 20.14)	-1.64 (-1.96 to -1.32)
**Turkey**	Severe anemia	1 (0 to 2)	5 (2 to 12)	5.27 (0 to 11.6)	1.4 (0 to 2.85)	0.46 (0.18 to 0.9)	-4.08 (-6.85 to -1.23)
**Turkmenistan**	**Anemia**	97 (48 to	47 (33 to 63)	-0.52 (-0.69 to	42.92 (37.77 to	47.09 (41.4 to	0.39 (0.22 to

		172)		-0.18)	48.24)	51.9)	0.57)
**Turkmenistan**	Mild anemia	53 (27 to 98)	27 (18 to 38)	-0.49 (-0.68 to -0.1)	23.43 (19.41 to 27.7)	27.62 (22.72 to 32.76)	0.66 (0.5 to 0.83)
**Turkmenistan**	Moderate anemia	41 (20 to 72)	18 (12 to 26)	-0.55 (-0.73 to -0.2)	18.19 (14.19 to 21.89)	18.5 (13.18 to 23.04)	0.11 (-0.09 to 0.3)
**Turkmenistan**	Severe anemia	3 (1 to 6)	1 (0 to 2)	-0.67 (-0.87 to -0.08)	1.3 (0.63 to 2.32)	0.97 (0.45 to 1.92)	-0.99 (-1.2 to -0.78)
**Tuvalu**	**Anemia**	0 (0 to 0)	1 (0 to 5)	23.59 (3.14 to 68)	41.28 (27.47 to 52.8)	45.69 (25.97 to 71.73)	0.19 (0 to 0.38)
**Tuvalu**	Mild anemia	0 (0 to 0)	1 (0 to 3)	25.92 (3.77 to 77.48)	22.31 (15.88 to 28.97)	27.38 (16.07 to 42.66)	0.54 (0.37 to 0.72)
**Tuvalu**	Moderate anemia	0 (0 to 0)	0 (0 to 2)	21.32 (2.54 to 62.69)	17.67 (10.31 to 24.63)	17.45 (8.72 to 29.77)	-0.2 (-0.42 to 0.02)
**Tuvalu**	Severe anemia	0 (0 to 0)	0 (0 to 0)	13.99 (0.91 to 58.38)	1.3 (0.58 to 2.51)	0.86 (0.35 to 1.79)	-1.52 (-1.71 to -1.33)
**Uganda**	**Anemia**	61397 (44485 to 79721)	66758 (45750 to 89597)	0.09 (-0.26 to 0.56)	31.76 (24.62 to 39.27)	33.09 (24.15 to 41.81)	0.29 (0.08 to 0.49)
**Uganda**	Mild anemia	30487 (22315 to 38110)	36343 (26609 to 47725)	0.19 (-0.15 to 0.65)	15.75 (12.88 to 18.69)	18 (14.32 to 21.78)	0.74 (0.53 to 0.95)
**Uganda**	Moderate anemia	28408 (19500 to 39334)	28676 (15926 to 43621)	0.01 (-0.46 to 0.75)	14.71 (10.32 to 19.57)	14.23 (8.28 to 20.81)	-0.05 (-0.3 to 0.2)
**Uganda**	Severe anemia	2501 (1153 to 4656)	1739 (853 to 3035)	-0.3 (-0.71 to 0.77)	1.3 (0.6 to 2.41)	0.86 (0.43 to 1.51)	-1.68 (-2.03 to -1.32)
**Ukraine**	**Anemia**	394 (154 to 820)	1665 (639 to 3453)	3.23 (0.63 to 8.76)	19.63 (11.61 to 29.83)	34.4 (16.23 to 55.73)	1.55 (1.36 to 1.73)
**Ukraine**	Mild anemia	294 (117 to 603)	1266 (489 to 2549)	3.3 (0.67 to 8.47)	14.66 (8.86 to 21.6)	26.14 (12.39 to 41.11)	1.68 (1.51 to 1.86)
**Ukraine**	Moderate anemia	96 (33 to 222)	388 (132 to 896)	3.05 (0.42 to 10.7)	4.77 (2.37 to 8.48)	8.02 (3.5 to 15.76)	1.22 (1.01 to 1.43)
**Ukraine**	Severe anemia	4 (1 to 11)	12 (3 to 33)	1.8 (-0.28 to 9.13)	0.21 (0.08 to 0.46)	0.24 (0.07 to 0.59)	-0.88 (-1.35 to -0.41)
**United Arab**	**Anemia**	3	56	19.79	39.86	48.85	-0.48

**Emirates**		(1 to 10)	(3 to 305)	(0.66 to 83.36)	(27.44 to 62.32)	(24.74 to 76.65)	(-1.47 to 0.52)
**United Arab Emirates**	Mild anemia	2 (0 to 6)	36 (2 to 201)	21.3 (0.77 to 90.71)	24.12 (16.33 to 36.81)	31.5 (16.81 to 49.52)	0.22 (-0.51 to 0.95)
**United Arab Emirates**	Moderat e anemia	1 (0 to 4)	19 (1 to 101)	17.93 (0.35 to 79.2)	14.84 (9.29 to 24.8)	16.72 (7.58 to 27.78)	-1.73 (-3.41 to -0.03)
**United Arab Emirates**	Severe anemia	0 (0 to 0)	1 (0 to 4)	10.62 (-0.3 to 58.07)	0.9 (0.37 to 1.81)	0.62 (0.21 to 1.32)	-1.7 (-2.36 to -1.02)
**United Kingdom**	**Anemia**	537 (258 to 986)	1073 (657 to 1622)	1 (0.16 to 2.66)	21.58 (15.26 to 28.04)	26.93 (18.11 to 35.96)	-0.08 (-0.6 to 0.45)
**United Kingdom**	Mild anemia	427 (207 to 783)	839 (521 to 1260)	0.97 (0.15 to 2.57)	17.17 (12.43 to 22.18)	21.08 (14.22 to 28.66)	-0.16 (-0.69 to 0.36)
**United Kingdom**	Moderat e anemia	107 (45 to 213)	226 (124 to 375)	1.12 (0.06 to 3.98)	4.29 (2.44 to 6.51)	5.68 (3.32 to 8.35)	0.21 (-0.32 to 0.74)
**United Kingdom**	Severe anemia	3 (1 to 7)	7 (2 to 16)	1.27 (-0.4 to 8.45)	0.12 (0.04 to 0.26)	0.18 (0.06 to 0.39)	0.88 (0.25 to 1.5)
**United Republic of Tanzania**	**Anemia**	151003 (120070 to 182284)	111718 (85634 to 138151)	-0.26 (-0.43 to -0.03)	67.99 (59.64 to 76.45)	61.89 (53.33 to 67.72)	-0.18 (-0.42 to 0.07)
**United Republic of Tanzania**	Mild anemia	50011 (38410 to 62170)	52114 (39487 to 64698)	0.04 (-0.23 to 0.44)	22.5 (19.19 to 26.07)	28.86 (25.19 to 32.59)	1.08 (0.85 to 1.3)
**United Republic of Tanzania**	Moderat e anemia	92083 (71931 to 113141)	55832 (41394 to 71371)	-0.39 (-0.56 to -0.17)	41.47 (35.32 to 47.52)	30.94 (25.11 to 35.25)	-0.9 (-1.14 to -0.66)
**United Republic of Tanzania**	Severe anemia	8909 (5865 to 12625)	3772 (2197 to 5902)	-0.58 (-0.78 to -0.22)	4.02 (2.7 to 5.62)	2.09 (1.28 to 3.14)	-2.25 (-2.49 to -2.02)
**United States of America**	**Anemia**	21999 (10855 to 38222)	12891 (7131 to 20506)	-0.41 (-0.63 to -0.05)	24.16 (19.79 to 27.66)	34.46 (23.32 to 42.18)	0.3 (-0.04 to 0.65)
**United States of America**	Mild anemia	16766 (8291 to 28807)	9860 (5547 to 15313)	-0.41 (-0.63 to -0.06)	18.42 (15.44 to 20.98)	26.37 (18.46 to 31.58)	0.35 (0.02 to 0.68)
**United States of America**	Moderat e anemia	5120 (2405 to 9544)	2950 (1483 to 5192)	-0.42 (-0.7 to 0.12)	5.62 (3.96 to 7.47)	7.87 (4.57 to 11.41)	0.13 (-0.25 to 0.51)
**United States of America**	Severe anemia	112 (44 to	81 (28 to	-0.28 (-0.76 to	0.12 (0.07 to	0.21 (0.08 to	1 (0.52 to

		239)	174)	1.01)	0.21)	0.45)	1.47)
**United States Virgin Islands**	**Anemia**	7 (4 to 11)	11 (8 to 16)	0.69 (0.14 to 1.55)	48.57 (42.08 to 54.6)	61.83 (56.93 to 66.89)	0.78 (0.62 to 0.93)
**United States Virgin Islands**	Mild anemia	4 (2 to 7)	7 (5 to 10)	0.73 (0.11 to 1.7)	30.32 (25.07 to 35.98)	39.41 (32.82 to 46.21)	0.94 (0.78 to 1.11)
**United States Virgin Islands**	Moderate anemia	2 (1 to 4)	4 (2 to 6)	0.64 (0.01 to 1.62)	17.47 (13.68 to 21.52)	21.66 (15.74 to 27.01)	0.53 (0.36 to 0.69)
**United States Virgin Islands**	Severe anemia	0 (0 to 0)	0 (0 to 0)	0.29 (-0.52 to 2.35)	0.79 (0.38 to 1.46)	0.77 (0.32 to 1.55)	-0.43 (-0.59 to -0.26)
**Uruguay**	**Anemia**	77 (27 to 169)	320 (160 to 544)	3.18 (1.31 to 7.39)	27.33 (22.16 to 33.03)	36.59 (28.7 to 44.38)	1.24 (1.09 to 1.39)
**Uruguay**	Mild anemia	57 (20 to 129)	248 (127 to 424)	3.35 (1.34 to 7.76)	20.27 (16.01 to 24.94)	28.32 (22.07 to 34.38)	1.38 (1.23 to 1.53)
**Uruguay**	Moderate anemia	19 (7 to 42)	70 (32 to 127)	2.71 (0.96 to 6.86)	6.81 (4.78 to 9.09)	8.06 (5.06 to 11.23)	0.85 (0.69 to 1.01)
**Uruguay**	Severe anemia	1 (0 to 2)	2 (1 to 4)	1.57 (-0.11 to 6.46)	0.25 (0.12 to 0.43)	0.2 (0.08 to 0.41)	-0.48 (-0.63 to -0.33)
**Uzbekistan**	**Anemia**	90 (48 to 150)	951 (524 to 1426)	9.56 (5.88 to 17.78)	62.58 (57.17 to 67.14)	60.09 (55.99 to 63.67)	0.34 (0.03 to 0.65)
**Uzbekistan**	Mild anemia	41 (21 to 69)	500 (265 to 769)	11.12 (6.3 to 21.67)	28.67 (24.52 to 34.04)	31.63 (25.92 to 37.56)	0.54 (0.19 to 0.88)
**Uzbekistan**	Moderate anemia	46 (24 to 78)	431 (225 to 686)	8.38 (4.62 to 16.43)	31.95 (27.22 to 35.65)	27.23 (21.8 to 31.9)	0.22 (-0.1 to 0.54)
**Uzbekistan**	Severe anemia	3 (1 to 6)	19 (7 to 44)	5.88 (1.13 to 20.32)	1.96 (1 to 3.19)	1.22 (0.53 to 2.44)	-1.12 (-1.39 to -0.85)
**Vanuatu**	**Anemia**	4 (1 to 26)	48 (4 to 322)	10.57 (0.9 to 75.88)	67.3 (42.39 to 86.68)	61.65 (37.61 to 84.95)	-0.16 (-0.35 to 0.03)
**Vanuatu**	Mild anemia	2 (0 to 11)	24 (2 to 143)	12.84 (1.4 to 91.37)	27.4 (18.72 to 35.52)	32.25 (20.13 to 47.14)	0.61 (0.38 to 0.85)
**Vanuatu**	Moderate anemia	2 (0 to 14)	23 (1 to 160)	9.25 (0.51 to 66.33)	36.26 (21.57 to 47.99)	27.09 (13.97 to 42.03)	-0.77 (-0.94 to -0.6)
**Vanuatu**	Severe	0	2	6.65	3.63	2.31	-1.61

	anemia	(0 to 1)	(0 to 11)	(0.08 to 55.88)	(1.59 to 6.5)	(0.78 to 5.17)	(-1.8 to -1.42)
**Venezuela (Bolivarian Republic of)**	**Anemia**	260 (184 to 368)	2178 (982 to 3687)	7.38 (3.53 to 11.85)	26.56 (22.9 to 30.62)	28.17 (16.93 to 35.05)	0.29 (-0.18 to 0.75)
**Venezuela (Bolivarian Republic of)**	Mild anemia	165 (112 to 235)	1538 (748 to 2636)	8.32 (4.1 to 13.56)	16.88 (13.73 to 20.39)	19.9 (12.39 to 25.21)	0.67 (0.18 to 1.16)
**Venezuela (Bolivarian Republic of)**	Moderate anemia	89 (59 to 130)	614 (244 to 1130)	5.87 (2.26 to 10.34)	9.14 (7.06 to 11.24)	7.94 (4.17 to 11.48)	-0.45 (-0.89 to -0.01)
**Venezuela (Bolivarian Republic of)**	Severe anemia	5 (2 to 10)	25 (8 to 60)	3.81 (0.82 to 10.31)	0.53 (0.28 to 0.93)	0.33 (0.13 to 0.64)	-1.66 (-1.95 to -1.37)
**Viet Nam**	**Anemia**	1230 (968 to 1549)	6550 (3970 to 10200)	4.32 (2.52 to 7.13)	31.88 (28.48 to 35.31)	21.83 (17.09 to 27.18)	-1.25 (-1.39 to -1.11)
**Viet Nam**	Mild anemia	776 (558 to 1008)	4765 (2729 to 7628)	5.14 (2.89 to 8.84)	20.11 (16.02 to 23.7)	15.87 (11.8 to 20.94)	-0.78 (-0.9 to -0.67)
**Viet Nam**	Moderate anemia	408 (298 to 531)	1724 (819 to 2988)	3.23 (1.18 to 6.19)	10.56 (8.45 to 12.47)	5.75 (3.36 to 8.31)	-1.81 (-1.96 to -1.66)
**Viet Nam**	Severe anemia	46 (13 to 188)	61 (23 to 136)	0.32 (-0.79 to 5.32)	1.2 (0.35 to 4.92)	0.2 (0.09 to 0.42)	-7.45 (-8.57 to -6.31)
**Yemen**	**Anemia**	88 (26 to 357)	828 (80 to 4435)	8.45 (0.15 to 54.64)	42.19 (30.72 to 61.11)	45.03 (25.92 to 71.81)	0.08 (-0.06 to 0.23)
**Yemen**	Mild anemia	37 (11 to 133)	342 (30 to 1799)	8.24 (0.08 to 53.18)	17.99 (13.64 to 24.37)	19.77 (11.82 to 35.84)	-0.21 (-0.46 to 0.04)
**Yemen**	Moderate anemia	44 (12 to 180)	434 (26 to 2618)	8.86 (-0.13 to 61.98)	21.04 (14.25 to 32.51)	22.44 (8.44 to 41.08)	0.33 (0.19 to 0.47)
**Yemen**	Severe anemia	7 (1 to 28)	52 (4 to 289)	6.83 (-0.24 to 46.94)	3.16 (1.66 to 5.55)	2.82 (1.14 to 5.09)	-0.13 (-0.28 to 0.03)
**Zambia**	**Anemia**	45562 (32770 to 58511)	81193 (63598 to 100611)	0.78 (0.27 to 1.57)	42.97 (34.16 to 52.24)	50.96 (42.59 to 59.44)	1.14 (0.83 to 1.45)
**Zambia**	Mild anemia	19421 (13661 to 25036)	35441 (27384 to 44964)	0.82 (0.29 to 1.68)	18.27 (14.95 to 22.24)	22.23 (18.65 to 26.45)	1.02 (0.73 to 1.31)
**Zambia**	Moderate anemia	23475 (16311 to 31158)	40854 (30621 to 52674)	0.74 (0.16 to 1.63)	22.17 (15.78 to 28.6)	25.65 (19.88 to 31.21)	1.15 (0.82 to 1.49)

**Zambia**	Severe anemia	2667 (1449 to 4396)	4898 (3291 to 7023)	0.84 (-0.06 to 2.68)	2.53 (1.39 to 4.32)	3.08 (2.14 to 4.49)	1.87 (1.41 to 2.34)
**Zimbabwe**	**Anemia**	58841 (18958 to 100757)	80752 (62813 to 97757)	0.37 (-0.21 to 2.98)	68.77 (36.69 to 84.21)	53.61 (44.57 to 58.43)	1.04 (0.18 to 1.9)
**Zimbabwe**	Mild anemia	34141 (10569 to 59574)	44987 (34853 to 55589)	0.32 (-0.28 to 3.2)	38.99 (24.71 to 49.41)	29.85 (25.64 to 33.55)	0.27 (-0.31 to 0.86)
**Zimbabwe**	Moderate anemia	23704 (8544 to 41547)	33807 (25322 to 42591)	0.43 (-0.23 to 2.95)	28.56 (10.79 to 41.3)	22.46 (17.12 to 26.1)	2.14 (0.78 to 3.51)
**Zimbabwe**	Severe anemia	995 (318 to 2101)	1959 (1240 to 2938)	0.97 (-0.18 to 5.98)	1.22 (0.33 to 2.34)	1.3 (0.85 to 1.89)	3.93 (2.31 to 5.57)

Data in parentheses are 95% uncertainty intervals for cases and prevalence, and 95% confidence intervals for EAPC.

Abbreviations: EAPC, estimated annual percentage change; NA, not available.
